# COVID-19 Vaccine Effectiveness Studies against Symptomatic and Severe Outcomes during the Omicron Period in Four Countries in the Eastern Mediterranean Region

**DOI:** 10.3390/vaccines12080906

**Published:** 2024-08-10

**Authors:** Manuela Runge, Zahra Karimian, Mehrnaz Kheirandish, Giulio Borghi, Natalie Wodniak, Kamal Fahmy, Carsten Mantel, Thomas Cherian, Zeinab Nabil Ahmed Said, Farid Najafi, Fatima Thneibat, Zia Ul-Haq, Sheraz Fazid, Iman Ibrahim Salama, Fatemeh Khosravi Shadmani, Ahmad Alrawashdeh, Shadrokh Sirous, Saverio Bellizzi, Amira Ahmed, Michael Lukwiya, Arash Rashidian, on behalf of the Consortium of Authors

**Affiliations:** 1 MMGH Consulting, 8049 Zurich, Switzerlandcheriant@mmglobalhealth.org (T.C.); 2 Division of Science, Information and Dissemination, WHO Regional Office for the Eastern Mediterranean, Cairo 11371, Egyptkheirandishm@who.int (M.K.); 3 Heidelberg Institute of Global Health, Heidelberg University Hospital, 69120 Heidelberg, Germany; 4 Division of Communicable Diseases, WHO Regional Office for the Eastern Mediterranean, Cairo 11516, Egypt; 5 Department of Medical Microbiology and Immunology, Faculty of Medicine (for Girls), Al-Azhar University, Cairo 11651, Egypt; 6 Research Center for Environmental Determinants of Health (RCEDH), Health Institute, Kermanshah University of Medical Sciences, Kermanshah 6713954658, Iranfatemeh.khosravi@kums.ac.ir (F.K.S.); 7 Jordan Ministry of Health, Amman 11118, Jordan; 8 Institute of Public Health and Social Sciences, Khyber Medical University, Peshawar 25100, Pakistansheraz.iph@kmu.edu.pk (S.F.); 9 Institute of Health and Wellbeing, University of Glasgow, Glasgow G12 8QQ, UK; 10 Department of Community Medicine Research, National Research Centre, Cairo 12622, Egypt; salamaiman@yahoo.com; 11 Department of Allied Medical Sciences, Jordan University of Science and Technology, Amman 3030, Jordan; 12 WHO Country Office for Iran, Tehran 8453193445, Iran; 13 WHO Country Office for Jordan, Amman 11181, Jordan; bellizzis@who.int; 14 WHO Country Office for Egypt, Cairo 11516, Egypt; 15 WHO Country Office for Pakistan, Islamabad P.O. Box 1013 44000, Pakistan

**Keywords:** SARS-CoV-2, COVID-19, vaccine effectiveness, Omicron, eastern Mediterranean region

## Abstract

Vaccine effectiveness (VE) studies provide real-world evidence to monitor vaccine performance and inform policy. The WHO Regional Office for the Eastern Mediterranean supported a regional study to assess the VE of COVID-19 vaccines against different clinical outcomes in four countries between June 2021 and August 2023. Health worker cohort studies were conducted in 2707 health workers in Egypt and Pakistan, of whom 171 experienced symptomatic laboratory-confirmed SARS-CoV-2 infection. Test-negative design case–control studies were conducted in Iran and Jordan in 4017 severe acute respiratory infection (SARI) patients (2347 controls and 1670 cases) during the Omicron variant dominant period. VE estimates were calculated for each study and pooled by study design for several vaccine types (BBIBP-CorV, AZD1222, BNT162b2, and mRNA-1273, among others). Among health workers, VE against symptomatic infection of a complete primary series could only be computed compared to partial vaccination, suggesting a benefit of providing an additional dose of mRNA vaccines (VE: 88.9%, 95%CI: 15.3–98.6%), while results were inconclusive for other vaccine products. Among SARI patients, VE against hospitalization of a complete primary series with any vaccine compared to non-vaccinated was 20.9% (95%CI: 4.5–34.5%). Effectiveness estimates for individual vaccines, booster doses, and secondary outcomes (intensive care unit admission and death) were inconclusive. Future VE studies will need to address challenges in both design and analysis when conducted late during a pandemic and will be able to utilize the strengthened capacities in countries.

## 1. Introduction

SARS-CoV-2 vaccines have been crucial in preventing severe COVID-19 disease and mortality, following their introduction in early 2021 [1,2]. The World Health Organization (WHO) supported the development [3] and global distribution [4] of COVID-19 vaccines as well as national vaccination campaigns in resource-constrained and emergency settings [5]. The WHO also developed technical guidance and supported the conduct of vaccine effectiveness studies [5,6,7,8].

COVID-19 vaccines that used different platforms are in use globally, including inactivated vaccines such as PiCoVacc (Coronavac^®^, Sinovac Biotech, Beijing, China), BBIBP-CorV (Covilo^®^, Sinopharm/BIBP, Wuhan, China), and BBV152 (Covaxin^®^, Bharat Biotech, Hyderabad, India); adenovirus vector vaccines such as AZD1222 (Vaxzevria^®^, AstraZeneca-Oxford, Oxford, UK), Gam-COVID-Vac (Sputnik V^®^, Gamaleya Institute, Moscow, Russia), AD5-nCOV (Convidecia^®^, CanSino, Tianjin, China), and Ad26.COV2.S (Janssen, Beerse, Belgium); and mRNA vaccines such as BNT162b2 (Comirnaty^®^, Pfizer/BioNTech, New York, NY, USA) and mRNA-1273 (Spikevax^®^, Moderna, Cambridge, MA, USA) [9]. Since the emergence of the Omicron SARS-CoV-2 variant in November 2021, decreased protection of primary series vaccination has been observed against infection and symptomatic disease compared to previous virus variants [10,11]. This decline has been attributed to a reduction in neutralization by vaccine-induced antibodies, underscoring the necessity for booster doses and leading to the development of vaccines adapted to variant strains [12,13,14].

The growing number of SARS-CoV-2 variants, combined with the multiplicity of vaccines [15,16] and waning immunity, contributes to a constantly evolving disease epidemiology. Hence, it is vital to gather data and evidence of vaccine effectiveness (VE) for the different vaccine products in use to inform policy recommendations and vaccination strategies.

In the WHO Eastern Mediterranean Region (EMR), results of over 20 VE studies in seven of the 22 countries had been published by 2023, most of them during pre-Omicron periods [17]. To strengthen the evidence on VE as well as to enhance the capacity of the countries to organize and carry out COVID-19 VE studies, the WHO Regional Office for the Eastern Mediterranean (WHO EMRO) supported two health worker cohort studies in Egypt and Pakistan and two test-negative design (TND) case–control studies in the Islamic Republic of Iran (Iran) and Jordan. These studies had been selected through an open call for proposals [5]. Across these countries, four to five transmission waves were recorded between January 2020 and May 2022, followed by a smaller wave in autumn 2022. As of November 2023, the cumulative number of locally reported COVID-19 cases was highest in Iran, followed by Jordan, Pakistan, and Egypt [18]. COVID-19 vaccination started in January/February 2021 [18] with over eleven vaccine products finally in use in the EMR [5]. As of November 2023, the coverage with a complete primary series in the total population was reported to be 41% in Egypt, 45% in Jordan, 60% in Pakistan, and 70% in Iran [19].

Using data from the four studies, VE was calculated for partial vaccination, the primary vaccination series, and the first booster dose for each study and pooled by study design. For the cohort studies, VE against laboratory-confirmed symptomatic infections among health workers was estimated. For the TND studies, VE against laboratory-confirmed infection leading to hospitalization, ICU admission, and death among SARI patients was estimated.

## 2. Materials and Methods

This study report follows the extended Strengthening the Reporting of Observational Studies in Epidemiology (STROBE-RECORD) statement (File S1) [20]. Ethical clearance was obtained by the study Principal Investigators from the relevant ethical review boards in the respective countries. Additionally, ethical clearance was secured from the WHO’s Eastern Mediterranean Research Ethics Review Committee.

### 2.1. Study Design

The cohort studies included any category of staff working at the study hospitals. The enrolment of health workers started in November 2021 in Pakistan (3 hospitals) and in June 2022 in Egypt (5 hospitals). The follow-up duration was twelve to thirteen months with fortnightly visits during which clinical and laboratory information was collected from health workers who reported symptoms according to the WHO COVID-19 case definition [21].

The TND case–control studies were conducted among hospitalized patients meeting the WHO SARI case definition of fever ≥38 °C and cough within ten days prior to hospitalization [22]. The studies spanned over twelve to thirteen months and included patients admitted as of May 2021 in Iran in 163 hospitals, and as of May 2022 in Jordan in 4 hospitals. The analysis was constrained to SARI patients admitted during the Omicron-dominant period, starting from 1 January 2022. Data were collected from patients aged twelve years and above, with Jordan’s study also including patients aged 5–11 years enrolled between February and June 2023. Cases and controls were matched based on time of hospital admission (within two weeks) in Iran, while no matching was carried out in Jordan (Table 1).

### 2.2. Data Collection

Data collection tools were adapted from WHO Guidance documents for each study design [6,7], as described in File S1.1. Data were entered, cleaned, and stored on the REDCap v14.1.2 platform (Nashville, Tennessee, USA) [23,24]. In the two cohort studies, data were collected at enrolment and at fortnightly follow-up visits and included clinical and laboratory information among health workers with symptoms of COVID-19. Health workers were tested for SARS-CoV-2 infection at enrolment and during follow-up visits if they reported relevant symptoms. Nasopharyngeal and oropharyngeal swabs were collected from symptomatic health workers and tested for SARS-CoV-2 using real-time quantitative reverse transcriptase polymerase chain reaction (qRT-PCR). In addition, self-reported qRT-PCR positive tests during the follow-up were included.

In the Iran study, two national databases were used to extract patient demographic information and clinical data during hospitalization (Medical Care Monitoring Centre) and data on COVID-19 vaccination (national immunization monitoring platform). In Jordan, demographic and hospital information were extracted from electronic hospital records and the national SARI database, and vaccination records were linked via an electronic COVID-19 vaccination platform. In Jordan, additional interviews with patients, their families, or physicians were conducted to complement electronic data. All patients were tested for SARS-CoV-2 using qRT-PCR within 48 h of hospital admission. Individuals testing positive for SARS-CoV-2 were considered cases, and individuals testing negative were considered controls.

### 2.3. Definitions

Partial vaccination was defined as the receipt of a single dose of a two-dose primary series; a complete primary series was defined as the receipt of two doses of any vaccine except for Ad26.COV2.S and Ad5-nCoV, for which a single dose constituted a primary series. Booster vaccination was defined as the receipt of one or more additional doses after the primary series. The receipt of a vaccine was counted if the dose was received ≥14 days prior to enrolment or each follow-up visit (cohort studies) or illness onset date (TND studies). In the cohort studies, the primary outcome was symptomatic laboratory-confirmed SARS-CoV-2 infection; no secondary outcomes occurred which could have been recorded. In the TND studies, the primary outcome was hospital admission, while secondary outcomes were ICU admission and in-hospital death (File S2, Table S2.1).

In the cohort studies, person-time at risk started at enrolment and ended with the final follow-up. The first thirteen days after the receipt of a new vaccine dose and the first 90 days after a laboratory-confirmed SARS-CoV-2 infection were excluded from the at-risk period. It was assumed that no symptomatic infections or new vaccinations happened during missed follow-up periods, unless such events were reported to study personnel at the next follow-up.

### 2.4. Quality Assurance and Technical Support for Field Studies

The technical support provided for the conduct of the studies has previously been described [5]. Technical support for the data management and cleaning of the study data included systematic and version-controlled data inspection and edits in REDCap. Additional data inspection was performed in R 4.3.2 (Vienna, Austria) [25] across all four studies between October and November 2023, until all remaining data inconsistencies were resolved. A harmonized analysis plan was generated from the individual study plans to facilitate the comparison of national study results and regional analyses by study design.

### 2.5. Statistical Analysis

The statistical analysis for the regional study included adjustments for covariates as described below. The VE was calculated for each study and pooled by study design following WHO guidelines on COVID-19 VE evaluations [26]. In this analysis, VE refers to absolute VE, using the unvaccinated as a comparator group, if not otherwise stated. Relative VE (rVE) was calculated for the cohort studies, using partially vaccinated subjects as the comparator group. Crude and adjusted VE estimates were calculated for partial and complete primary series and booster vaccination per vaccine product or platform, as well as for all vaccines combined. Subgroup analysis by time since vaccination and age was performed if there was a sample size of ≥5 individuals per subgroup. Crude analyses of study-specific and pooled analyses included a random effect at the hospital level for Egypt, Pakistan, and Jordan and at the province level for Iran. For multivariable analysis, potential confounders were selected a priori (age, sex, and for TND studies, calendar week) and by using a change-in-estimate [27] threshold of ten percentage points. The same confounders were used for assessing absolute and relative VE. A complete case analysis was conducted, and study participants with missing data for exposure, outcome, or covariates were excluded. Statistical analysis was performed in R [25].

#### 2.5.1. Cohort Studies

In the cohort studies, VE was estimated using Cox regression, computed as (1 − hazard ratio) ∗ 100%. Vaccination status was included as a time-varying exposure. The final multivariable models were adjusted for age, sex, and hospital site. No other variables met the inclusion criteria for sample size or from the change-in-estimate approach. Due to the low numbers of unvaccinated individuals in Pakistan (n = 4, 0.3%), rVE was calculated to allow for a comparison across both cohort studies. Absolute VE estimates for the Egypt study are reported in the Supplementary File (File S2, Section 4.3). Sensitivity analysis was performed censoring all participants who had missed at least three consecutive follow-up visits.

#### 2.5.2. TND Studies

In the TND studies, VE was estimated using logistic regression, computed as (1 − odds ratio) ∗ 100%. Case–control classification was defined as the dependent variable and vaccination status as the independent variable. To assess secondary outcomes (ICU admission, death), the dataset was filtered for patients for which the outcomes were recorded. Multivariable models were adjusted for age group (12–44, 45–64, and 65+ years), sex, and week of illness onset. In the analysis for Jordan, additional covariates derived from the change-in-estimate evaluation were number of comorbidities, smoking history, and type of care or support received at home (care status); no variables met the change-in-estimate criteria for Iran. Stratified analysis was conducted by time between the last vaccine dose and illness onset (<90, 90–179, and ≥180 days) and for SARI patients ≥65 years of age by vaccine product.

## 3. Results

### 3.1. Description of Study Population

#### 3.1.1. Cohort Studies

A total of 2963 health workers were invited to participate (1257 [42.4%] in Egypt and 1706 [57.6%] in Pakistan). Among these, 2707 health workers completed the first follow-up and were included in the analysis (1235 [45.6%] in Egypt and 1472 [54.4%] in Pakistan). In the Egypt study, the median follow-up time was 336 days; 174 participants missed at least one follow-up visit, and 453 health workers dropped out prematurely, with 283 only missing the final follow-up visit. In the Pakistan study, the median follow-up time was 295 days; 20 participants missed at least one follow-up visit, and 1464 participants dropped out prematurely, with 1072 only missing the final follow-up visit (File S2, Tables S2.4 and S2.5).

Throughout the follow-up period, a total of 2267 symptomatic events were recorded in both studies combined (86.3% of which were from Pakistan). Of those with reported symptoms, 1548 (68.3%) were tested for SARS-CoV-2, the testing rate being 72.8% in Pakistan and 39.4% in Egypt. In total, 171 symptomatic laboratory-confirmed COVID-19 patients were documented (89.5% occurring in Pakistan). Reasons for missed tests in Egypt included logistical problems and participants’ refusal to undergo nasopharyngeal swabbing, while specific reasons were unknown for Pakistan.

**Figure 1:**
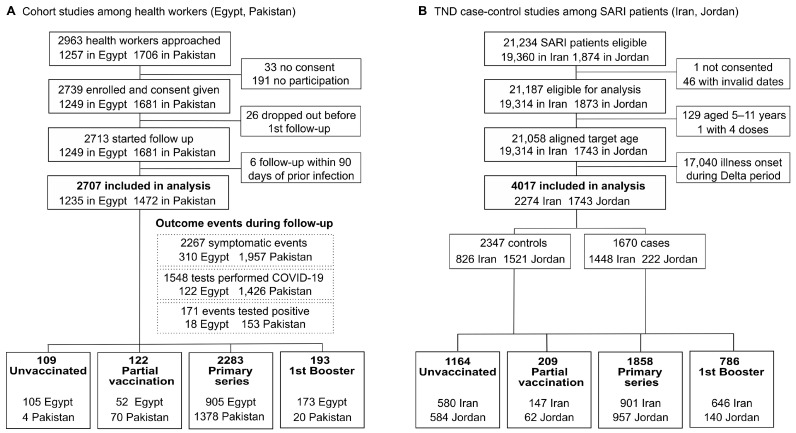
Sample size flowchart for the (**A**) cohort studies and (**B**) TND studies. Vaccination status in cohort studies shown at start of follow-up.

**Figure 2:**
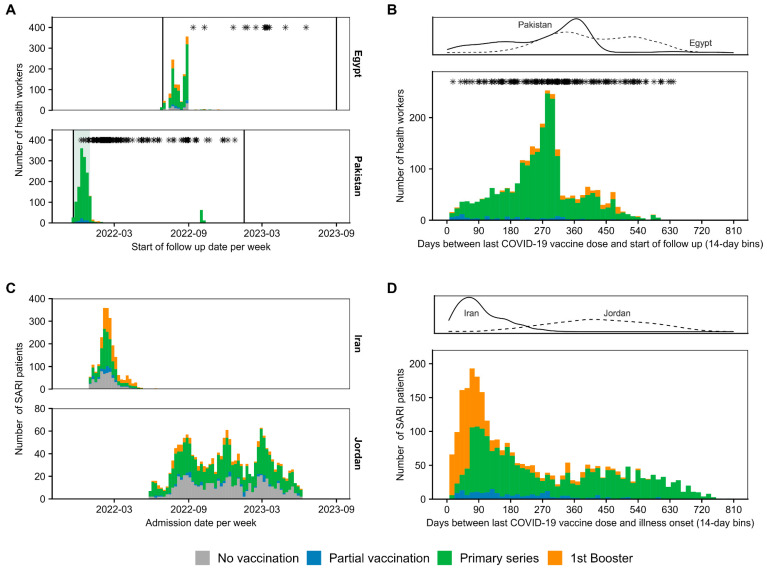
Study population by vaccination status and time since vaccination over time. (**A**) Number of health workers in the two cohort studies by start of follow-up. The two vertical lines indicate start and end of study period. The grey shaded area indicates the pre-Omicron-dominant period. (**B**) Days since last vaccination and start of follow-up in the pooled cohort dataset (bottom) and corresponding density plot (top). The star symbol indicates outcome events during follow-up. (**C**) SARI patients in the two TND studies by admission date and vaccination status. (**D**) Days since vaccination and illness onset date in pooled TND studies (bottom) and corresponding density plot (top).

At the start of the follow-up period, the majority of health workers in Egypt and nearly all health workers in Pakistan had completed the primary vaccination series (73.3% and 93.6%, respectively) and some health workers had received a booster dose (14.0% in Egypt, 1.3% in Pakistan) (Figure 1A, File S2, Table S2.2). During follow-up, one health worker in Egypt received a booster, and 146 doses were administered to 138 health workers in Pakistan (22 doses contributing to primary series vaccinations, and 124 booster doses). The start of the follow-up of participants by vaccination status is shown in Figure 2A.

#### 3.1.2. TND Studies

A total of 21,234 SARI patients were eligible for inclusion (19,360 in Iran and 1874 in Jordan). After data cleaning, including alignment to vaccination status definition (File S2, Table S2.9), the restriction of the analysis to the Omicron period, and the exclusion of individuals aged <12 years, 2274 SARI patients from Iran and 1743 patients from Jordan were included in the analysis. In total, 1670 patients tested positive for SARS-CoV-2 (86.7% from Iran), and 2347 patients tested negative (64.8% from Jordan). Within studies in Iran, 63.7% of patients included in the analysis met the WHO SARI case definition, whereas in Jordan, only 12.7% were cases. Across both studies, 29.0% were unvaccinated, 5.2% were partially vaccinated, 46.2% had completed the primary series, and 19.6% had received a first booster dose. In Iran, fewer study participants were unvaccinated or had completed the primary series while a much higher proportion had received the booster dose than in Jordan (25.5% vs. 33.5%, 6.5% vs. 3.6% and 39.6% vs. 54.9%, 28.4% vs. 8.0% for unvaccinated, partially vaccinated, primary series, and booster dose in Iran and Jordan, respectively) (Figure 1B, File S2, Tables S2.11 and S2.13). The admission dates of enrolled SARI patients by vaccination status are shown in Figure 2C.

### 3.2. Description and Comparison of Study Populations

#### 3.2.1. Cohort Studies

Most participants were between 28 and 45 years of age, with more health workers above 50 years in Egypt than in Pakistan (23.5% vs. 6.7%). There was a higher proportion of female participants in Egypt than in Pakistan (55.7% vs. 25.1%). The majority of participants in both studies had either a secondary school or a university degree. One-fifth were medical doctors, and the remaining participants included nursing personnel, paramedical staff, and ‘other professions. Of all participants in both studies, 76.8% reported not having had a SARS-CoV-2 infection earlier. Among vaccinated health workers, more than half were vaccinated with BBIBP-CorV (57.4% overall; 41.1% in Egypt; 70.0% in Pakistan), 14% with either PiCoVacc (14.2% overall, 7.2% in Egypt; 19.6% in Pakistan) or AZD1222 (14.9% overall, 33.2% in Egypt; 0.8% in Pakistan), and 5.0% had received heterologous schedules (9.4% in Egypt; 1.6% in Pakistan), among other vaccine types with lower frequency (File S2, Table S2.2). Health workers had received their last vaccine dose on average eight months before the start of follow-up (maximum 20 months in both studies), with a slightly longer time since vaccination in Egypt (Figure 2B). Additional baseline characteristics per study compared by participants’ vaccination status are provided in File S2, Tables S2.3–S2.5.

#### 3.2.2. TND Studies

The patient’s ages ranged between 12 and 99 years; most were between 43 and 74 years old (Table S2.10). The interval between the last vaccine dose and illness onset varied between studies, with an average of 3.4 and 14.6 months (maximum 12 and 27.5 months) in Iran and Jordan, respectively (Figure 2D). Among those who were vaccinated, in the pooled dataset, 60.1% were vaccinated with BBIBP-CorV (75% of them from Iran), 20.6% were vaccinated with BNT162b2 (all from Jordan), 13.0% were vaccinated with AZD1222, and 6.3% received ‘other’ vaccines (the latter two mainly in Iran) (Table S2.10). Additional baseline characteristics per study by case–control group and by vaccination status are available in the Supplementary File (File S2, Tables S2.10–15).

### 3.3. Vaccine Effectiveness

#### 3.3.1. Absolute VE against Laboratory-Confirmed Symptomatic Infection: Cohort Studies

In Egypt, the adjusted VE against lab-confirmed symptomatic infection following the primary series was 66.1% (95%CI: −53.0% to 92.5%) for AZD1222, 63.3% (95%CI: −60.1% to 91.6%) for BBIBP-CorV, and 57.0% (−Inf to 95.5%) for PiCoVacc (File S2, Figure S2.1). Sensitivity analysis, censoring participants who had missed at least three consecutive follow-up visits, showed no major changes, and VE estimates remained inconclusive (File S2, Figure S2.2 and Table S2.6).

#### 3.3.2. Relative VE against Laboratory-Confirmed Symptomatic Infection: Cohort Studies

In Egypt, the unadjusted rVE against lab-confirmed symptomatic infection following the primary series of AZD1222 was 84.9% (95%CI: 17.5% to 97.2%). After adjusting for potential confounders, rVE was statistically non-significant (rVE: 82.7%, 95%CI: −2.4% to 97.1%). In Pakistan, rVE point estimates were consistently close to or below zero. In the pooled dataset, mRNA vaccines, (BNT162b2 and mRNA-1273) showed the added benefit of a complete primary series over partial vaccination (rVE: 88.9% 95%CI: 15.3% to 98.6%).

**Figure 3:**
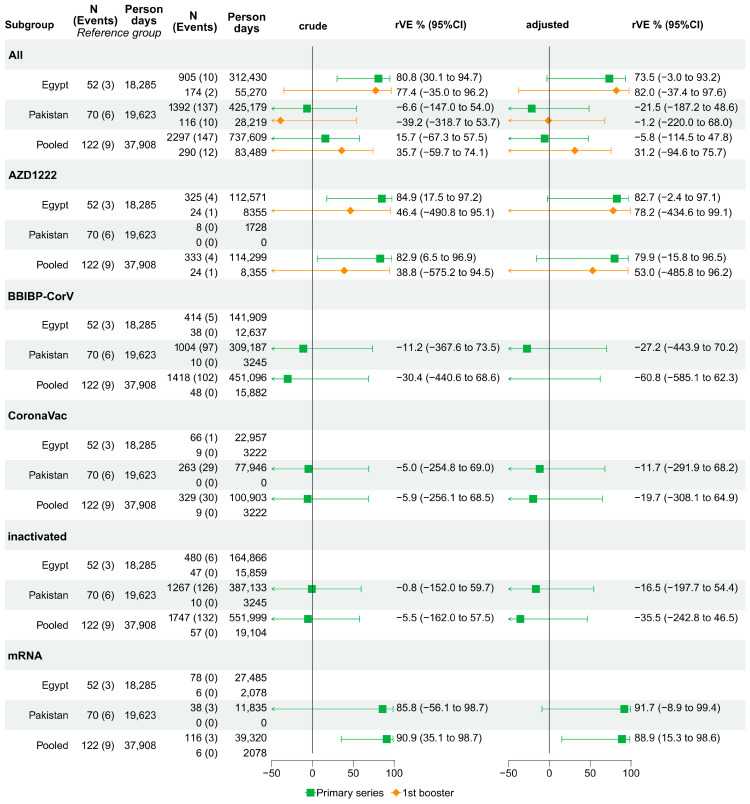
VE of a primary series and a booster compared to partial vaccination against lab-confirmed symptomatic COVID-19 infection among health workers compared to partial vaccination (reference). Blank VE estimates indicate insufficient data to be computed.

The booster doses showed mixed and inconclusive rVE estimates (rVE_Egypt_: 82% 95%CI: −37.4% to 97.6%, rVE_Pakistan_: −1.2%, 95%CI: −220.0% to 68.0%, rVE_pooled_: 32.2%, 95%CI: −94.6% to 75.7%) (Figure 3).

In subgroup analysis, the rVE of the primary series showed added protection among those previously infected with SARS-CoV-2 in Egypt (rVE: 96.3, 95%CI: 39.2% to 99.8%), as did the rVE among those vaccinated more than 180 days ago (rVE: 74.9%, 95%CI: 2.9% to 93.5%) (File S2, Figures S2.3 and S2.4 for Pakistan and pooled dataset). When data from participants who missed three consecutive follow-up visits were censored in the sensitivity analysis of the Egypt data (three consecutive missed follow-up visits had not been recorded in Pakistan), the rVE remained significant (rVE_previousCOVID-19_: 96.2%, 95%CI: 38.0% to 99.8%; rVE_tsincevacc180_: 77.0%, 95%CI: 8.8% to 94.2%). Additionally, in the overall cohort in Egypt, primary series vaccination with any vaccine and with AZD1222 showed some protection (rVE_any_vaccine_: 75.3%, 95%CI: 2.1% to 93.8%. rVEAZD1222: 85.4%, 95%CI: 7.7% to 97.7%) (File S2, Figure S2.6, Table S2.7). When using 60 days (instead of 90) since previous infection before participants contributed to person-time at risk, the results did not change substantially (File S2, Figure S2.7, Table S2.8). 

#### 3.3.3. Absolute VE against Hospitalization: SARI TND Studies

The adjusted study-specific VE against hospitalization following partial vaccination with any vaccine product showed higher protection in Iran than in Jordan and was only statistically significant in Iran (VE_Iran_: 37.2%, 95%CI: 7.9% to 57.2%, VE_Jordan_: 20.3%, 95%CI: −79.6 to 64.6%). The pooled estimate was in the same range as that observed in Iran (VE: 32.9%, 95%CI: 5.0% to 52.7%). When assessing vaccine product-specific VEs, partial vaccination with BBIBP-CorV showed moderate protection in Iran (VE_Iran_BBIBP-CorV:_ 41.4%, 95%CI: 9.4% to 62.1%).

**Figure 4:**
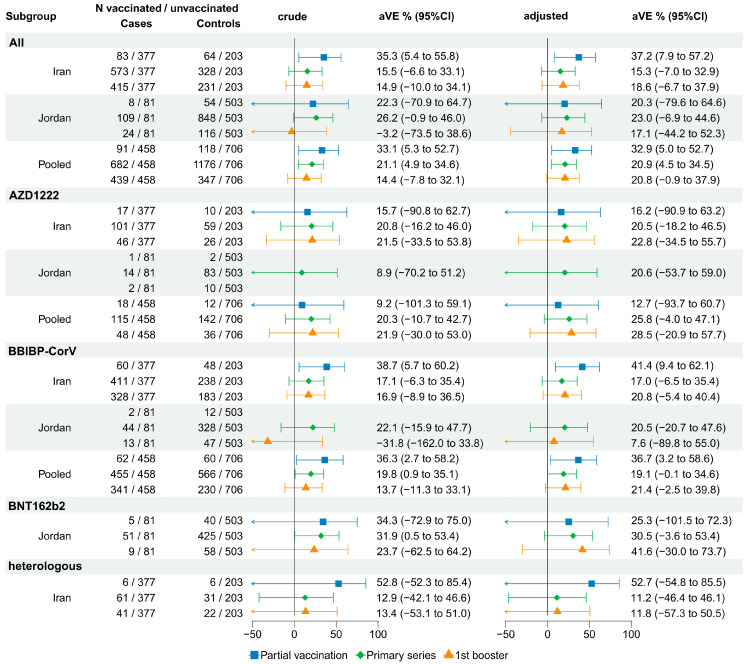
VE of a primary series and a booster compared to no vaccination against hospitalization among SARI patients. Blank VE fields indicate insufficient data to be computed.

The VE of a complete primary series with any vaccine product was non-significant in both Iran (VE_Iran:_ 15.3%, 95%CI: −7.0% to 32.9%) and Jordan (VE_Jordan_: 18.6%, 95%CI: −6.7 to 37.9%). The pooled VE estimate showed a low level of protection (VE: 20.9% 95%CI: 4.5% to 34.5%). Primary series vaccination with AZD1222 was inconclusive in both studies (VE_Iran_: 20.5%, 95%CI: −18.2% to 46.5%, VE_Jordan:_ 20.6%, 95%CI −53.7% to 59.0%) (Figure 4).

In the stratified analysis by time since vaccination (File S2, Figures S2.10–11), primary series vaccination with BBIBP-CorV showed a moderate VE of 33.0% (95%CI: 4.7% to 52.9%) in Iran among those vaccinated within 90 days prior to illness onset, and within 90 to 180 days (VE: 53.9%, 95%CI: 9.2% to 76.5%). In the pooled dataset, primary series vaccination with AZD1222 showed moderate protection with vaccination occurring 90–180 days prior to illness onset (VE: 42.9%, 95%CI: 4.0% to 66.0%), while primary series vaccination with BBIBP-CorV showed protection for patients vaccinated >180 days prior to illness onset (VE: 27.4%, 95%CI 2.8% to 45.7%). In addition, the VE of primary series vaccination >180 days prior to illness onset with ‘other’ vaccines, attributable mostly to Gam-COVID-Vac in Iran, was estimated to be 60.5% (95%CI: 2.2% to 84.0%).

Protection against hospitalization from a booster dose was statistically significant only in the pooled dataset among patients vaccinated with BBIBP-CorV within 90 days prior to illness onset (VE_pooled_BBIBP-CorV_: 28.1%, 3.5% to 46.4%).

When the pooled analysis was restricted to patients aged ≥65 years, VE was slightly lower for primary series vaccination with AZD1222 (VE 21.0% 95%CI: −23.4 to 45.9%), and much lower following a booster dose with the same vaccine (VE 6.9% 95%CI: −73.7% to 50.1%). The trend in decreased effect sizes by vaccination status was stronger for vaccination with BBIBP-CorV with an estimated VE of 16.9% (95%CI: −16.3% to 40.6%) for the primary series and −1.5% (95%CI: −44.3% to 28.6%) for a booster dose (File S2, Figure S2.12, Table S2.18).

#### 3.3.4. Absolute VE against ICU Admission and/or Death: SARI TND Studies

**Figure 5:**
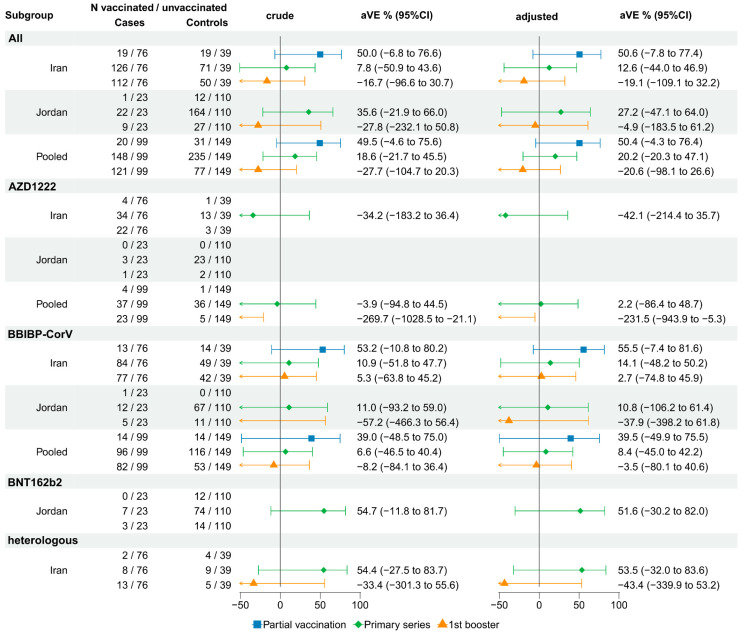
VE of a primary series and a booster compared to no vaccination against ICU admission and/or death among SARI patients in the TND studies. Blank VE estimates indicate insufficient data to be computed.

The study-specific VE against ICU admission and/or death in Iran showed a decreasing trend in effect size for the BBIBP-CorV vaccine with an increasing number of doses, although all estimates were non-significant (VE_partial_ 55.5%, 95%CI −7.4% to 81.6%; VE_primary_ 14.1%: 95%CI −48.2% to 50.2%; VE_booster_ 2.7%: 95%CI −74.8% to 45.9%). The VE of a booster dose with the BBIBP-CorV vaccine was close to zero or negative across the three datasets assessed, and non-significant (VE_Iran_ 2.7%, 95%CI −74.8% to 45.9%; VE_Jordan_ −37.9%, 95%CI −398.2% to 61.8%; VE_pooled_ −3.5%: 95%CI −80.1% to 40.6%) (Figure 5). VE estimates when both outcomes, ICU admission and death, were assessed separately showed similar trends and were statistically non-significant (File S2, Figures S2.8 and S2.9), with similar findings in subgroup analysis (File S2, Figure S2.13, Table S2.19).

## 4. Discussion

Between 2021 and 2023, WHO EMRO provided coordinated support to four country research teams to assess the VE of COVID-19 vaccines against symptomatic infection, hospitalization, ICU admission, and in-hospital death. In Egypt and Pakistan, two cohort studies were conducted including a total of 2707 health workers. Primary series vaccination with mRNA vaccines was found to be effective against laboratory-confirmed symptomatic infections in the pooled analysis, while the separate studies yielded mostly low and inconclusive VE estimates across the subgroups evaluated (vaccine type, previous infection status, and time since vaccinations). In Iran and Jordan, TND case–control studies were conducted, including a total of 4017 hospitalized SARI patients during the Omicron-dominant period. Partial vaccination with BBIBP-CorV and primary series vaccination with any vaccine product showed some protective effectiveness against SARI hospitalization. Primary series vaccination with AZD1222 also showed a protective effect against hospitalization for individuals vaccinated 90–180 days before illness onset. Significant protection against ICU admission and or death could not be demonstrated. These findings, which differ from results of studies conducted in the region earlier during the COVID-19 pandemic, indicate a growing difficulty in obtaining reliable vaccine estimates later in the pandemic, particularly during the Omicron-dominant period.

Low or even negative VEs are likely attributable to a multiplicity of factors [2] such as evolving viral variants, increasing immunity following natural infection, and waning immunity with increasing time since vaccination. At the time the studies were conducted, SARS-CoV-2 seropositivity rates in the region were already very high [28] and most study participants likely benefited from infection- or vaccine-induced immunity. The consequent depletion of susceptible individuals among the unvaccinated group likely contributed to the low observed VEs [29]. No data on SARS-CoV-2 seropositivity were available in the study regions to allow for adjustment in the analysis. Additionally, due to challenges and variations in data collection between the TND studies, analyses could not be stratified by previous COVID-19 infection. Even if such data had been available, given that most infections were asymptomatic and, hence, undetectable, such analyses would have been challenging. In addition, with over twelve months between vaccination and illness in most patients in Jordan’s TND study and over six months in many study participants in the other studies, we would expect VE to have waned [30].

Other studies in the EMR showed varying, although often higher, VE against symptomatic and severe outcomes across different virus variant periods [17]. A health worker cohort study in Egypt in 2021 evaluated the VE of the BBIBP-CorV vaccine against symptomatic lab-confirmed SARS-CoV-2 infection and reported a significant VE of 67% [31]. A similarly high absolute VE, however, was not replicated in the Egypt study presented here. Notable differences between the two studies include longer time since vaccination in the present study and different circulating virus variants (Delta in the former, Omicron in the latter). The low VE estimates in the cohort studies may also be attributed to the reduced effectiveness of inactivated virus vaccines during the Omicron period, as indicated in previous studies [32]. Relative VE measures only quantify additional benefits relative to pre-existing protection and are not comparable to other studies, in particular when using partial vaccination as a comparator [33]. Given the high seropositive levels and likely mix of natural and vaccine-induced immunity, the VE estimates are likely not indicative of the added benefit of completing the primary series compared to partial vaccination.

In Iran, a previous TND study had been conducted in one province in 2021 [34], when the Alpha and Delta virus variants were dominant, with an estimated VE against hospitalization of 85%. In the present studies in Iran and Jordan, similar VE estimates were not observed, likely due to a difference in time since vaccination and the emergence of variants against which the vaccines were less effective. During the later Omicron-dominant pandemic period, SARS-CoV-2 infections may also often have been a coincidental finding in persons initially admitted for another illness rather than for COVID-19, hence causing a higher likelihood of severe disease or death [10], which would further reduce the observed VE in hospitalized patients [35].

Other regional studies conducted during the Omicron-dominant period also reported VE estimates of less than 50%. These include studies in the WHO regions of the Americas [36], Europe [37], and Africa [38]. Similar findings were made in other global studies [11,39]. Results from Latin America show a highly protective VE for the pre-Omicron period, which was lower during the Omicron period and decreased by time since vaccination, with inconclusive and negative VE after 180 days since vaccination [36]. In a European study, mixed VE estimates were found, with AZD1222 not showing a conclusive VE for primary series vaccination while being associated with a significant VE for the booster dose during the first 180 days after vaccination [37].

Some limitations in the reported studies are worth highlighting. The cohort studies had relatively low sample sizes, limiting the ability to adequately adjust for confounding. Incomplete testing rates further reduced the low number of events, which may have introduced selection bias. Health workers included in the cohort studies are a special group and VEs could be confounded by differential rates of vaccination or SARS-CoV-2 exposure. Those with a higher risk of exposure due to direct contact with COVID-19 patients may have been prioritized for vaccination. In the analysis, it was not possible to adjust for risk of exposure due to the small numbers.

In the TND studies, cases and controls should ideally have been matched by age, and although post hoc adjustments for age did not significantly change the VE estimates, any differences might have been masked by the wide confidence intervals around the VE estimates. Additionally, the misclassification of cases and controls from imperfect tests or differential delays between illness onset and date of testing cannot be excluded.

The pooled analysis of the data combined for each study design aimed to address the smaller sample sizes of the individual studies. However, the large heterogeneity between studies did not increase the precision of the VE estimates. In the cohort studies, unequal numbers in the outcome (with the majority of cases in the vaccinated having occurred in Pakistan) and exposure categories (with only four unvaccinated health workers recorded in Pakistan), together with the differential start of enrolment contributed to heterogeneity. Heterogeneity between the TND studies included different case–control ratios, and differences in the time between vaccination and illness onset (which was much longer in patients in Jordan), and in the vaccine products used. Stratification often resulted in only a single country included in a subgroup (e.g., BNT162b2 only appearing in Jordan for the TND studies). File S3 provides a summary of the technical implementation challenges encountered and the resulting lessons learned.

Overall, many of these challenges and limitations might be attributable to the timing of the studies and the altered epidemiological context compared to the first year of the pandemic. Therefore, the VE estimates from these four studies might not be reliable on their own and should be considered as part of a broader collection of VE studies in the region over various time periods.

Despite these challenges, the studies collectively contributed to the broader pool of VE data in the region and can be utilized in regional analyses to enhance the understanding of COVID-19 VE during different periods of a pandemic. VE in a pandemic situation is expected to change over time because of the depletion of susceptibles, the evolution of the pathogen and waning immunity. Hence, the timing of VE studies is critical to obtain interpretable VE estimates that indicate the true performance of a vaccine. Future studies may aim to obtain VE from a wider range of primary studies. Ultimately, improved pandemic preparedness including preparedness for conducting VE studies, as well as readiness in utilizing existing networks or surveillance systems should allow for more effective data collection and the generation of valid VE estimates.

The studies have important implications for the enhancement of the research ecosystem and national capacities in low- and middle-income countries for the conduct of VE studies in response to key threats and future pandemics [5,8]. The timely conduct of such studies requires long-term investment for example in establishing and enabling sentinel surveillance sites to conduct relevant studies. The benefits of such an approach are likely to extend beyond VE studies alone.

## 5. Conclusions

The mostly inconclusive results of the four studies with wide confidence intervals demonstrate the increasing challenge of obtaining reliable VE estimates late during the COVID-19 pandemic in the Omicron dominant period. The challenges encountered in both study implementation and analysis underscore the importance of regional collaboration and flexibility to adapt to context-specific needs while minimizing deviations from standardized protocols. Importantly, the coordinated WHO support for conducting these studies enhanced the national capacity for VE studies during future pandemics and large disease outbreaks. The research experience, in addition to the work on ‘Solidarity’ randomized clinical trials, will further inform WHO support for large-scale national studies [28,40,41], while country research capacities can be utilized for the continuous assessment of new vaccines in support of country decision-making.

## Figures and Tables

**Table 1:** Overview of study characteristics by country.

Study Design	Cohort Studies		TND Studies		
Country	Egypt	Pakistan	Iran		Jordan
Study period	06/2022–08/2023	11/2021–12/2022 ^1^	05/2021–12/2021 ^2^	01/2022–06/2022	06/2022–06/2023
Number of provinces	3	1	8	8	4
Number of hospitals	5	3	159	100	4
Number of individuals included in analysis	1235	1472	17,040 ^2^	2274	1873
Vaccines used in study	BBIBP-CorV, PiCoVacc, BNT162b2, mRNA-1273 ^§^, AZD1222, Other ^§^	BBIBP-CorV, PiCoVacc, BNT162b2 ^§^, mRNA-1273 ^§^, AZD1222 ^§^, Gam-COVID-Vac ^§^, Other ^§^	BBIBP-CorV, AZD1222, Gam-COVID-Vac ^§^, Other ^3^		BBIBP-CorV, BNT162b2, AZD1222, Gam-COVID-Vac ^§^

^1^ Follow-up was conducted for 487 participants for two months during the Delta variant period; ^2^ Listed for completeness, as excluded from the analysis for Omicron period. ^3^ Other vaccines were combined into one category during data collection and included vaccines produced in Iran: (BBV152, COVIran Barekat, Mivac, PastoCovac, COVAX-19, and Noora); ^§^ Used by ≤5% of the study population (1st dose).

## Data Availability

The datasets presented in this article are not readily available/accessible due to additional authorization requirements from the participating countries’ PIs who shared health data for their respective national studies with the WHO. Requests to access any datasets or codes/scripts used for data analysis should be directed to the corresponding author.

## Supplementary Materials

The following supporting information can be downloaded at: https://www.mdpi.com/article/10.3390/vaccines12080906/s1, File S1: STROBE/RECORD checklist, File S2: Methodological details and additional results, File S3: Challenges and lessons learned, File S4: Consortium of Authors.
