# Supplementary material for: COVID-19 Vaccine Effectiveness Studies against Symptomatic and Severe Outcomes during the Omicron Period in Four Countries in the Eastern Mediterranean Region
Source: Vaccines (Basel). 2024 Aug 10;12(8):906. doi: 10.3390/vaccines12080906 (PMC11360574; doi:10.3390/vaccines12080906)
Supplement: Supplementary file 1 [file vaccines-12-00906-s001.zip › File S1 - STROBE.pdf]

COVID-19 vaccine effectiveness against symptomatic and severe outcomes during the Omicron period in four countries in the Eastern Mediterranean Region

Table S1.1: Checklist of STROBE/RECORD[1] items reported in the manuscript and supplement

	Item No.	STROBE items	RECORD items	Location in manuscript where items are reported
**TITLE AND ABSTRACT**				
**Title and abstract**	**1**	**a)** Indicate the study's design with a commonly used term in the title or the abstract<br><br>**b)** Provide in the abstract an informative and balanced summary of what was done and what was found	**RECORD 1.1:** The type of data used should be specified in the title or abstract. When possible, the name of the databases used should be included.<br><br>**RECORD 1.2:** If applicable, the geographic region and timeframe within which the study took place should be reported in the title or abstract.<br><br>**RECORD 1.3:** If linkage between databases was conducted for the study, this should be clearly stated in the title or abstract.	The type of data by study design is mentioned in the abstract<br><br>The geographic region is mentioned in the title and specific countries in the abstract<br><br>Databases used described in methods section 2.1 Study design, population and data sources, applicable to TND studies
**INTRODUCTION**				
**Background rationale**	**2**	Explain the scientific background and rationale for the investigation being reported		Described in the introduction

[1] https://www.record-statement.org/ .

**Objectives**	**3**	State specific objectives, including any prespecified hypotheses		Research objectives stated in last paragraph of the introduction
**METHODS**				
**Study Design**	**4**	Present key elements of study design early in the paper		Study design mentioned in abstract and key characteristics described in methods under 2.1 Study design, population and data sources
**Setting**	**5**	Describe the setting, locations, and relevant dates, including periods of recruitment, exposure, follow-up, and data collection		Described in methods under 2.1 Study design, population and data sources and Table 1
**Participants**	**6**	**a)** Cohort study - Give the eligibility criteria, and the sources and methods of selection of participants. Describe methods of follow-up<br><br>Case-control study - Give the eligibility criteria, and the sources and methods of case ascertainment and control selection. Give the rationale for the choice of cases and controls Cross-sectional study - Give the eligibility criteria, and the sources and methods of selection of participants	**RECORD 6.1:** The methods of study population selection (such as codes or algorithms used to identify subjects) should be listed in detail. If this is not possible, an explanation should be provided.<br><br>**RECORD 6.2:** Any validation studies of the codes or algorithms used to select the population should be referenced. If validation was conducted for this study and not published elsewhere, detailed methods and results should be provided.	Eligibility criteria mentioned in 2.1 Study design, population and data sources<br><br>SARI case definition specified in methods.<br><br>The codes used for the selection of SARI patients followed the WHO-EURO guidance document for SARI TND studies[2], as listed in the Annex 2 of that document.

[2] Estimating COVID-19 vaccine effectiveness against severe acute respiratory infections (SARI) hospitalisations associated with laboratory-confirmed SARS-CoV-2. An evaluation using the test-negative design: guidance Document. Copenhagen: WHO Regional Office for Europe; 2021. Licence: CC BY-NC-SA 3.0 IGO.

		**b)** Cohort study - For matched studies, give matching criteria and number of exposed and unexposed<br><br>Case-control study - For matched studies, give matching criteria and the number of controls per case	**RECORD 6.3**: If the study involved linkage of databases, consider use of a flow diagram or other graphical display to demonstrate the data linkage process, including the number of individuals with linked data at each stage.	Details of data linkage are provided in the supplementary files, File S2.
**Variables**	**7**	Clearly define all outcomes, exposures, predictors, potential confounders, and effect modifiers. Give diagnostic criteria, if applicable.	**RECORD 7.1:** A complete list of codes and algorithms used to classify exposures, outcomes, confounders, and effect modifiers should be provided. If these cannot be reported, an explanation should be provided.	Definition of outcome and exposure provided in methods 2.2. "Definition of exposure and outcomes" and Supplementary File 2 Table S2.1.<br><br>Selection of potential confounders are described in "2.3. Statistical analysis"
**Data sources/ measurement**	**8**	For each variable of interest, give sources of data and details of methods of assessment (measurement).<br>Describe comparability of assessment methods if there is more than one group		
**Bias**	**9**	Describe any efforts to address potential sources of bias		Selection of potential confounders are described in "2.3. Statistical analysis"<br><br>The section also includes sensitivity analysis performed to assess influence of bias

**Study size**	**10**	Explain how the study size was arrived at		Mentioned in Supplement File S2, Section 1
**Quantitative variables**	**11**	Explain how quantitative variables were handled in the analyses. If applicable, describe which groupings were chosen, and why		Mentioned in Supplement File S2, Section 3
**Statistical methods**	**12**	**a)** Describe all statistical methods, including those used to control for confounding<br><br>**b)** Describe any methods used to examine subgroups and interactions<br><br>**c)** Explain how missing data were addressed<br><br>**d)** Cohort study - If applicable, explain how loss to follow-up was addressed<br><br>Case-control study - If applicable, explain how matching of cases and controls was addressed<br>Cross-sectional study - If applicable, describe analytical methods taking account of sampling strategy<br><br>**e)** Describe any sensitivity analyses		Described in "2.3. Statistical analysis"<br>With further details provided in Supplement File S2 Section ,<br><br>d)<br>for cohort studies described in "2.2. Definition of exposure and outcomes" paragraph 3<br>and "2.3. Data access and statistical analysis" paragraph 4<br><br>for TND studies described in "2.1. Study design, population and data sources" paragraph 3<br><br>e) sensitivity analysis performed mentioned in "2.3. Data access and statistical analysis" paragraph 5 and 6"

**Data access and cleaning methods**			**RECORD 12.1:** Authors should describe the extent to which the investigators had access to the database population used to create the study population.	Described in “2.3. Data access and statistical analysis” paragraph 1
			**RECORD 12.2:** Authors should provide information on the data cleaning methods used in the study.	Described in “2.3. Data access and statistical analysis” paragraph 1
**Linkage**			**RECORD 12.3**: State whether the study included person-level, institutional-level, or other data linkage across two or more databases. The methods of linkage and methods of linkage quality evaluation should be provided.	Details of data linkage are provided in the supplementary files, File S2.
**Participants**	**13**	**a)** Report the numbers of individuals at each stage of the study (e.g., numbers potentially eligible, examined for eligibility, confirmed eligible, included in the study, completing follow-up, and analysed)<br><br>**b)** Give reasons for nonparticipation at each stage.<br><br>**c)** Consider use of a flow diagram	**RECORD 13.1**: Describe in detail the selection of the persons included in the study (i.e., study population selection) including filtering based on data quality, data availability and linkage. The selection of included persons can be described in the text and/or by means of the study flow diagram.	Described in “Description of studies and sample sizes” paragraph 1,2,<br><br>c) see Figure 1
**Descriptive data**	**14**	**a)** Give characteristics of study participants (e.g., demographic, clinical, social) and information on		Provided in section “3.2. Description of study population” and File S1 “baseline

		exposures and potential confounders<br><br>**b)** Indicate the number of participants with missing data for each variable of interest<br><br>**c)** Cohort study - summarise follow-up time (e.g., average and total amount)		characteristics tables", which for cohort studies include c)
**Outcome data**	**15**	Cohort study - Report numbers of outcome events or summary measures over time<br>Case-control study - Report numbers in each exposure category, or summary measures of exposure<br>Cross-sectional study - Report numbers of outcome events or summary measures		Provided in "3.2. Description of study population" Figure 2.
**Main results**	**16**	**a)** Give unadjusted estimates and, if applicable, confounder adjusted estimates and their precision (e.g., 95% confidence interval). Make clear which confounders were adjusted for and why they were included<br><br>**b)** Report category boundaries when continuous variables were categorized		Crude and adjusted estimates provided in result figures 3,4,5.<br><br>Categorization of variables described in methods section "2.3. Data access and statistical analysis".<br><br>c) not applicable.

		**c)** If relevant, consider translating estimates of relative risk into absolute risk for a meaningful time period		
**Other analyses**	**17**	Report other analyses done—e.g., analyses of subgroups and interactions, and sensitivity analyses		Sensitivity and subgroup analysis performed mentioned in "2.3. Data access and statistical analysis" paragraph 5 and 6". Results presented in supplement File S1.
**Key results**	**18**	Summarise key results with reference to study objectives		Summarized in abstract and first paragraph of the discussion section.
**Limitations**	**19**	Discuss limitations of the study, taking into account sources of potential bias or imprecision. Discuss both direction and magnitude of any potential bias	**RECORD 19.1:** Discuss the implications of using data that were not created or collected to answer the specific research question(s). Include discussion of misclassification bias, unmeasured confounding, missing data, and changing eligibility over time, as they pertain to the study being reported.	Provided in discussion paragraphs 2,3,4,5
**Interpretation**	**20**	Give a cautious overall interpretation of results considering objectives, limitations, multiplicity of analyses, results from similar studies, and other relevant evidence		Provided in discussion paragraphs 1-8

Generalisability	**21**	Discuss the generalisability (external validity) of the study results		Addressed in discussion paragraphs 6
Funding	**22**	Give the source of funding and the role of the funders for the present study and, if applicable, for the original study on which the present article is based		Provided in designated declarations under "Funding"
Accessibility of protocol, raw data, and programming code			**RECORD 22.1:** Authors should provide information on how to access any supplemental information such as the study protocol, raw data, or programming code.	Provided in designated declarations under "Data Availability Statement"
