# Supplementary material for: COVID-19 Vaccine Effectiveness Studies against Symptomatic and Severe Outcomes during the Omicron Period in Four Countries in the Eastern Mediterranean Region
Source: Vaccines (Basel). 2024 Aug 10;12(8):906. doi: 10.3390/vaccines12080906 (PMC11360574; doi:10.3390/vaccines12080906)
Supplement: Supplementary file 1 [file vaccines-12-00906-s001.zip › File S2 - Additional methods and results.pdf]

COVID-19 vaccine effectiveness against symptomatic and severe outcomes during the Omicron period in four countries in the Eastern Mediterranean Region

Supplementary File S2: Methodological details and additional results

Contents

List of figures 3

List of tables 3

1 Sample size calculation and data collection 4

1.1 HW Cohort studies 4

1.2 SARI TND case control studies 5

2 Definitions 6

3 Statistical analysis 7

3.1 Covariate selection – HW cohort 7

3.2 Covariate selection – TND studies 8

4 Additional results from the cohort studies 10

4.1 Baseline characteristics 10

4.2 Missed follow ups 19

4.3 Results for absolute VE in Egypt's cohort study 20

4.3.1 Subgroup analysis 20

4.3.2 Sensitivity analysis 20

4.4 Subgroup analysis: estimated rVE in cohort studies from Egypt and Pakistan 22

4.5 Sensitivity analysis: estimated rVE in cohort studies from Egypt and Pakistan 25

Egypt 25

Pakistan 26

5 Additional results from the TND studies 27

5.1 Baseline characteristics 27

Iran 29

Jordan 34

Pooled 40

5.2 VE results separate for ICU admission and death 44

5.3 Subgroup analysis results in TND studies 46

List of figures

Figure S2.1: aVE results of primary series vaccination compared to unvaccinated in Egypt................20
Figure S2.2: Sensitivity analysis: aVE for partial vaccination, primary series and booster compared to unvaccinated in Egypt. 21
Figure S2.3: rVE by vaccination status and subgroups estimated from Egypt's study..........................22
Figure S2.4: rVE by vaccination status and subgroups estimated from Pakistan's study 23
Figure S2.5: rVE by vaccination status and subgroups estimated from pooled cohort data 24
Figure S2.6: Sensitivity analysis: rVE for partial vaccination, primary series and booster compared to unvaccinated in Egypt. 25
Figure S2.6: Sensitivity analysis: rVE for partial vaccination, primary series and booster compared to unvaccinated in Pakistan...26
Figure S2.7: Absolute VE against ICU admission among SARI patients in the TND studies. Blank VEs indicate insufficient data to be computed. 44
Figure S2.8: Absolute VE against death among SARI patients in the TND studies. Blank VEs indicate insufficient data to be computed..45
Figure S2.9: Adjusted VE against hospitalization by time since vaccination using pooled data from the two TND studies in Iran and Jordan. 46
Figure S2.10: Adjusted VE against ICU admission or death (in-hospital-mortality), by time since vaccination using pooled data from the two TND studies in Iran and Jordan. 48
Figure S2.11: Adjusted VE against hospitalization by age group , all above 11 years, and age 65 years and older, using pooled data from the two TND studies in Iran and Jordan. Age group 5-11 only applicable to Jordan...49
Figure S2.12: Adjusted VE against ICU admission and or death (in-hospital mortality), by age group , all above 11 years, and age 65 years and older, using pooled data from the two TND studies in Iran and Jordan. Age group 5-11 only applicable to Jordan...51

List of tables

Table S2.1: Definition of exposure and outcomes according to study design. 6
Table S2.2: Baseline characteristics at start of follow-up by country and pooled for cohort studies in Egypt and Pakistan 10
***Table S2.3:** Baseline characteristics at the start of follow up among participants in pooled cohort data by vaccination status*...12
Table S2.4: Baseline characteristics at the start of follow up among participants in Egypt's study by vaccination status...15
Table S2.5: Baseline characteristics at the start of follow up among participants in Pakistan's study by vaccination status...17
Table S2.6: Sensitivity analysis: aVE for partial vaccination, primary series and booster compared to unvaccinated in Egypt. 21
Table S2.7: Sensitivity analysis with 60 days (instead of 90) since previous infection before contributing to person-time at risk. Summary rVE results of primary series vaccination compared to partial vaccination in Egypt 25
Table S2.8: Sensitivity analysis with 60 days (instead of 90) since previous infection before contributing to person-time at risk. Summary rVE results of primary series vaccination compared to partial vaccination in Pakistan...26
Table S2.9: Adjustment in vaccination status for Iran and Jordan 27

Table S2.10: Baseline characteristics of SARI patients included in the TND studies compared across studies and combined 27
Table S2.11: Baseline characteristics of SARI patients included in the TND studies in Iran by case control group 29
Table S2.12: Baseline characteristics of SARI patients included in the TND studies in Iran by vaccination status 32
Table S2.13: Baseline characteristics of SARI patients included in the TND studies in Jordan by case control group 34
Table S2.14: Baseline characteristics of SARI patients included in the TND studies in Jordan by vaccination status 38
Table S2.15: Baseline characteristics of SARI patients included in the TND studies in Jordan by case control group 40
Table S2.16: Adjusted VE against hospitalization by time since vaccination using pooled data from the TND studies 46
Table S2.17: VE against ICU admission/death by time since vaccination using pooled data from the TND studies 48
Table S2.18: Adjusted VE against hospitalization by age group , all above 11 years, and age 65 years and older, using pooled data from the two TND studies in Iran and Jordan. Age group 5-11 only applicable to Jordan. 50
Table S2.19: Adjusted VE against ICU admission and or death (in-hospital mortality), by age group , all above 11 years, and age 65 years and older, using pooled data from the two TND studies in Iran and Jordan. Age group 5-11 only applicable to Jordan. 51

1 Sample size calculation and data collection

1.1 HW Cohort studies

Sample size calculation

Sample size calculation followed the recommended process of Table 2 in the WHO guidelines, considering incidence of SARS-CoV-2 infection among unvaccinated participants during a one-year study. In Egypt's study it was assumed that 20% of unvaccinated people would be infected with SARS-CoV-2 over a period of 12 months (Yearly rate-unvaccinated = 0.2), the expected vaccine effectiveness (VE) was 60%, and the expected coverage was 90%. Under these assumptions, the calculated sample size was 1,006 participants, increasing to 1,250 when considering a 20% lost follow up rate. The study in Pakistan assumed the same coverage, but and higher VE of 70%, and a lower prevalence among unvaccinated people (5%, yearly rate-unvaccinated=0.1). The calculated sample size was 1,356, increasing to 1,627 after accounting for a 20% lost follow up rate.

Sampling strategy

In Egypt's study a random sample of health workers was recruited within selected hospitals. The project team visited all hospital departments, and draw a random sample from each department. This was done according to the number of health workers in each department and their acceptance to participate in the study. In Pakistan's study a two staged proportionate random sampling for selection of the study participants was employed. In stage 1, a total of 3 tertiary care hospitals were selected from the total 12 followed by random sampling to select the required number of health care workers

from these hospitals. The number of healthcare workers by different cadres were selected on proportionate basis in each of the hospital.

Data collection
Hospital vaccine registries were used to collect and triangulate information about vaccination history for the participants enrolled. Personal information was collected through four different questionnaires, adapted from the WHO Guidance document[1]: i) an enrolment questionnaire collecting socio-demographic information, health status, vaccination history, occurrence of previous SARS-CoV-2 infections, and work-related information; ii) a biweekly follow-up questionnaire, collecting information on new COVID-19 symptoms, and changes in vaccination status, exposure to COVID-19 cases, and in work-related activities since last follow-up; iii) a virology questionnaire collecting the type of virological test performed, test timing (enrolment, symptoms development, or follow-up), result, and date of testing; and a serology questionnaire for COVID-19 tests performed during the study including at enrolment.

1.2 SARI TND case control studies

Severe Acute Respiratory Infection (SARI) case definition
Based on the WHO SARI case definition as follows:
- a hospitalised person (minimum of 24 hours stay) with acute respiratory infection, with
- a history of fever or measured fever of ≥ 38 C°
- and cough
- with symptom onset within the last 10 days.

Sample size calculation
In Iran's study, the sample size calculation considered a VE of 50%, and a 60% COVID-19 vaccine coverage among controls. Aiming for 5% precision, the minimum sample size is estimated as 12,688 cases and 12,688 controls, which was increased by 20%, resulting in a total sample size of 30,450. The case-control ratio was considered to be 1:1. The number of cases and controls in each university were selected proportional to the size of the total population covered by each university. Eligible cases and controls were included from the selected university until the predefined sample size was reached.

In Jordan's study, the case-control ratio was considered to be 1:2 and sample size was calculated assuming a VE of 70%, a 60% vaccination coverage, and that 33% of SARI cases would have COVID-19. Using a desired precision of ±10%, the estimated sample size was approximately 1,000 cases and 2,000 controls.

Data collection
In Iran's study, two national databases were used in Iran, extracting patient demographic information and clinical data during hospitalization (Medical Care Monitoring Centre) and COVID-19 vaccination data from the national immunization monitoring platform (Integrated Health Record System, known as 'SIB' in Iran).

[1] WHO. Cohort study to measure COVID-19 vaccine effectiveness among health workers in WHO European Region: Guidance Document. Copenhagen: WHO Regional Office for Europe; 2021.

In Jordan's study, demographic and hospital course information was extracted from electronic hospital records ("Hakeem" program) and the national SARI database, and vaccination records were linked via an electronic COVID-19 vaccination platform. In Jordan's study, additional interviews with patients, their families, or physicians were conducted to complement the electronic data.

Record linkage

In Iran's study, the two national databases were linked by participating provinces using national IDs. Two workshops were held by the central study team to ensure consistency in the linking process.

In Jordan's study, the study ID was assigned a unique Redcap ID when being entered on the platform, and was not linked electronically to any other ID. However, in some sites, the hospital ID was linked to the Redcap ID in a hospital logbook to collect additional data if needed. Since the study population were SARI patients, the SARI ID was linked with the Redcap ID in a laboratory logbook to collect data on laboratory results and being able to enter the lab data on redcap.

2 Definitions

Table S2.1: *Definition of exposure and outcomes according to study design.*

Variable		Definition
**Exposure - Vaccination**	Unvaccinated	Individuals who did not receive any dose of COVID-19 vaccine at enrolment or during follow up in the cohort studies or before illness onset in the TND studies.
	Partially vaccinated	Individuals who received one dose of vaccine (based on a two-dose vaccine) at least 14 days before enrolment, follow-up, or event date in the cohort studies, or at least 14 days before illness onset in the TND studies.
	Primary series (complete vaccination)	Individuals who received completed the primary series of two doses of vaccine (based on a two-dose vaccine) or one dose for Ad26.COV2.S or AD5-nCOV vaccine since at least 14 days before enrolment, follow-up, or event date in the cohort studies or at least 14 days before illness onset in the TND studies.
	Booster dose	Individuals who received one additional dose after the primary series at least 14 days before enrolment, follow-up, or event date or at least 14 days before illness onset in the TND studies. For the purposes of this analysis, individuals were considered partially, fully, or booster vaccinated if the most recent dose of vaccine was received at least 14 days before symptom onset.
**Outcome**	Symptomatic lab-confirmed SARS-CoV-2 infection	**Cohort study:** SARS-CoV-2 infection confirmed by RT-PCR test (reported or done by the research team) in any participant who reported symptoms compatible with COVID-19 (WHO case definition[2]) during follow up.
	Hospital admission	**Cohort study:** SARS-CoV-2 infection confirmed by RT-PCR test (reported or done by the research team) in any participant who reported to have been admitted to the hospital for that episode of disease.<br>**TND study:** a patient hospitalized with severe acute respiratory illness (SARI) symptoms, with a respiratory sample positive for SARS-CoV-2 by RT-PCR, either within 48 hours of hospital admission or documented within 14 days prior to hospital admission.

[2] WHO COVID-19: case definitions: updated in Public health surveillance for COVID-19, 22 July 2022

	ICU admission	**Cohort study:** SARS-CoV-2 infection confirmed by RT-PCR test (reported or done by the research team) in any participant who reported to have been admitted to the ICU for that episode of disease.<br>**TND study:** defined as admission to the ICU among SARI patients (including both cases and controls).
	Death (in-hospital mortality)	**Cohort study:** in-hospital death following hospitalization due to SARS-CoV-2 infection confirmed by RT-PCR (reported or done by the research team).<br>**TND study:** defined as deaths in hospital among SARI patients (including both cases and controls).

Person time at risk (cohort studies)

Person-time at risk was calculated starting either at enrolment or 90 days after reported symptom onset if HWs had a laboratory-confirmed SARS-CoV-2 infection prior to or at enrolment. The duration of person-time ended either at the end of the study or at last follow-up. The first thirteen days after receipt of a new vaccination dose (whether a primary series or booster dose) and the first 90 days after a laboratory-confirmed SARS-CoV-2 infection were excluded from the time at risk. In case of a missed follow-up visit, participants were assumed to not have had events or vaccination during the missing period if they did not report either of these events when asked by the research teams at their next follow-up visit

3 Statistical analysis

VE in the cohort studies was estimated using Cox regression analysis, computed as (1 - hazard ratio) * 100 and in the TND studies was estimated using logistic regression analysis, computed as (1 - odds ratio) * 100. Crude and adjusted analyses were performed, including hospital site (Egypt, Pakistan, Jordan) and province (Iran) as a random effect in both.

In addition to hospital site, multivariable models were adjusted based on a priori knowledge of confounders: age group and sex, and for the TND studies, calendar time (week of illness onset, adjusted as a continuous variable). Other potential confounders were assessed using the change-in-estimate approach for each country's full dataset; if adjusted VE differed from crude VE by more than 10% for any variable (threshold based on[3]), the variable was considered for inclusion into multivariable models. Similar variables (e.g., individual comorbidities vs. the cumulative number of comorbidities) identified using the change-in-estimate approach were assessed, and one of the appropriate variables was included in multivariable models. In order to be included in multivariable models for subset analyses (e.g., for each vaccine type), the covariates were required to have a sample size of at least five for each subgroup. A complete case analysis was conducted, hence patients with missing data for either exposure, outcome or covariates were excluded from the analysis.

3.1 Covariate selection – HW cohort

In Egypt, the approach described above led to considering close contact with a confirmed COVID-19 case and smoking as additional covariates in the adjusted model. However, due to convergence problems related to the low number of events (less than five) in some categories of each variable, no additional covariates beside age, sex, and hospital site could be included.

[3] WHO. Evaluation of COVID-19 vaccine effectiveness. 2021.

In Pakistan, the number of people living in the household was considered as an additional covariate, however due to a large proportion of participants with missing information, including 30 with a recorded lab-confirmed SARS-CoV-2 infection, the variable was not included in the final model adjustments.

The full list of variables assessed for inclusion into the multivariable models is shown below, the first named category in categorical variables used as the comparator:

- age as continuous variable,
- age groups: <30, 30-40, 40-50, >50
- sex: Female, Male
- body mass index (BMI): <25, 25-29, 30-39, ≥40;
- education: none, primary, secondary, university;
- smoking habits: yes, no;
- number of comorbidities: 1, 2, ≥3, based on reported diabetes, cardiovascular diseases, hypertension, immune deficiency, lung disease, asthma, cancer, renal disease, liver disease, rheumatic disease;
- type of HW: medical doctor, nursing or midwifery personnel, paramedical, other;
- number of people living in the household: <3, 3-6, 6-9, ≥10;
- in the past 14 days, frequency of contact with patients or people affected by COVID-19: 0, 1-5, 6-10, ≥10;
- less than one meter contact with patients or people affected by COVID-19: yes, no;
- in the past 14 days, frequency of participation in indoor events: 0, 1-2, ≥3;
- in the past 14 days, frequency of public transportation use: 0, 1-2, ≥3;
- frequency of mask wearing: always, often, sometimes, rarely, never;
- frequency of hand hygiene practice: most of the time/always, occasionally/rarely, never;
- frequency of IPC standard precaution adoption: most of the time/always, occasionally/rarely, never.

3.2 Covariate selection – TND studies

No additional variables were identified for adjustment for Iran, and comorbidities, smoking status, and patient care status were identified as additional covariates for Jordan.

The following covariates were included per study in adjusted models:

- **Iran**: age group, sex, number of week of symptom onset since study start (‘week’)
- **Jordan**: age group, sex, ‘week’, number of comorbidities, smoking, patient care status
- **Pooled**: age group, sex, ‘week’

The full list of variables assessed for inclusion into the multivariable models is shown below, the first named category in categorical variables used as the comparator:

- age group, categorized into <12, 12-17, 18-44, 44-54, 64+ years
- sex (female, male),
- calendar time (week of illness onset, adjusted as a continuous variable),

- comorbidity (yes/no) for: heart disease, hypertension, cancer, renal disease, liver disease, lung disease, asthma, diabetes, immunological disease, neurological disease, rheumatological disease, anemia, obesity,
- cumulative number of comorbidities (0, 1, 2+),
- smoking history (no never, former smoker, yes currently),
- health care worker status (yes/no),
- number of hospital visits in the previous year,
- previous hospitalization for COVID-19 (yes/no),
- pregnant (yes/no),
- patient care status (independent, home care, assisted living)

4 Additional results from the cohort studies

4.1 Baseline characteristics

Table S2.2: *Baseline characteristics at start of follow-up by country and pooled for cohort studies in Egypt and Pakistan*

		Study country		
**Variable**	**Overall**, N = 2,707[1]	**Egypt**, N = 1,235[1]	**Pakistan**, N = 1,472[1]	**p-value**[2]
**Age (years) - Median (IQR)**	35.0 (28.0, 45.0)	40.0 (30.0, 49.0)	32.0 (27.0, 39.0)	**<0.001**
**Age group**				**<0.001**
<30	805 (29.7%)	264 (21.4%)	541 (36.8%)	
30-39	923 (34.1%)	345 (27.9%)	578 (39.3%)	
40-49	591 (21.8%)	336 (27.2%)	255 (17.3%)	
50-59	363 (13.4%)	265 (21.5%)	98 (6.7%)	
60+	25 (0.9%)	25 (2.0%)	0 (0.0%)	
**Sex**				**<0.001**
Female	1,058 (39.1%)	688 (55.7%)	370 (25.1%)	
Male	1,649 (60.9%)	547 (44.3%)	1,102 (74.9%)	
**Highest level of education**				**<0.001**
None	43 (1.6%)	0 (0.0%)	43 (2.9%)	
Primary	268 (9.9%)	183 (14.8%)	85 (5.8%)	
Secondary	1,141 (42.1%)	773 (62.6%)	368 (25.0%)	
University	1,244 (46.0%)	279 (22.6%)	965 (65.6%)	
Prefer not to answer	11 (0.4%)	0 (0.0%)	11 (0.7%)	
**Smoking history**				**<0.001**
No, I never smoked	2,425 (89.6%)	1,089 (88.2%)	1,336 (90.8%)	
I smoke smoking more than one year ago	96 (3.5%)	76 (6.2%)	20 (1.4%)	
Yes, I currently smoke	186 (6.9%)	70 (5.7%)	116 (7.9%)	
**BMI**				**<0.001**
<25	1,066 (39.4%)	270 (21.9%)	796 (54.1%)	
25-29	1,010 (37.3%)	531 (43.0%)	479 (32.5%)	
30-39	584 (21.6%)	394 (31.9%)	190 (12.9%)	
>=40	47 (1.7%)	40 (3.2%)	7 (0.5%)	
**Regularly taking medication**	419 (15.5%)	302 (24.5%)	117 (7.9%)	**<0.001**
(Missing)	1	1	0	
**Healthcare worker**				**<0.001**
MD	573 (21.2%)	156 (12.6%)	417 (28.3%)	
Nursing/midwifery personnel	977 (36.1%)	523 (42.3%)	454 (30.8%)	
Other	904 (33.4%)	436 (35.3%)	468 (31.8%)	
Paramedical	253 (9.3%)	120 (9.7%)	133 (9.0%)	
**Total number of lab-confirmed symptomatic infections during follow-up**				**<0.001**
0	2,541 (93.9%)	1,217 (98.5%)	1,324 (89.9%)	
1	161 (5.9%)	18 (1.5%)	143 (9.7%)	
2	5 (0.2%)	0 (0.0%)	5 (0.3%)	
**Previous COVID-19 infection**				**<0.001**
0	2,078 (76.8%)	866 (70.1%)	1,212 (82.3%)	

Variable	Overall, N = 2,707[1]	Study country<br>Egypt, N = 1,235[1]	Pakistan, N = 1,472[1]	p-value[2]
1	629 (23.2%)	369 (29.9%)	260 (17.7%)	
**Vaccination status at start of follow up**				**<0.001**
Unvaccinated	109 (4.0%)	105 (8.5%)	4 (0.3%)	
Partial	122 (4.5%)	52 (4.2%)	70 (4.8%)	
Primary series	2,283 (84.3%)	905 (73.3%)	1,378 (93.6%)	
Booster	193 (7.1%)	173 (14.0%)	20 (1.4%)	
**Vaccination status at end of follow up**				**<0.001**
Unvaccinated	109 (4.0%)	105 (8.5%)	4 (0.3%)	
Partial	109 (4.0%)	52 (4.2%)	57 (3.9%)	
Primary series	2,198 (81.2%)	904 (73.2%)	1,294 (87.9%)	
Booster	291 (10.7%)	174 (14.1%)	117 (7.9%)	
**Type of COVID-19 vaccine (all three doses) at start of follow up**				
AZD1222	387 (14.9%)	375 (33.2%)	12 (0.8%)	
BBIBP-CorV	1,491 (57.4%)	464 (41.1%)	1,027 (70.0%)	
AD5-nCOV	59 (2.3%)	0 (0.0%)	59 (4.0%)	
PiCoVacc	368 (14.2%)	81 (7.2%)	287 (19.6%)	
Gam-Covid-Vac	17 (0.7%)	0 (0.0%)	17 (1.2%)	
Ad26.COV2.S	12 (0.5%)	12 (1.1%)	0 (0.0%)	
mRNA-1273	43 (1.7%)	11 (1.0%)	32 (2.2%)	
BNT162b2	89 (3.4%)	81 (7.2%)	8 (0.5%)	
Heterologous	129 (5.0%)	106 (9.4%)	23 (1.6%)	
Unknown	3 (0.1%)	0 (0.0%)	3 (0.2%)	
(Missing)	109	105	4	
**Days between most recent vaccination and illness onset**				**<0.001**
<90	280 (10.8%)	71 (6.3%)	209 (14.2%)	
90-180	408 (15.7%)	133 (11.8%)	275 (18.7%)	
>180	1,910 (73.5%)	926 (81.9%)	984 (67.0%)	
(Missing)	109	105	4	
**Hospital site**				**<0.001**
Alhussein Hospital	300 (11.1%)	300 (24.3%)	0 (0.0%)	
Alzahraa Hospital	294 (10.9%)	294 (23.8%)	0 (0.0%)	
Assiut Hospital	150 (5.5%)	150 (12.1%)	0 (0.0%)	
Bab Alsharia Hospital	290 (10.7%)	290 (23.5%)	0 (0.0%)	
Damiatta Hospital	201 (7.4%)	201 (16.3%)	0 (0.0%)	
Kohat Hospital	395 (14.6%)	0 (0.0%)	395 (26.8%)	
Mardan Hospital	569 (21.0%)	0 (0.0%)	569 (38.7%)	
Swat Hospital	508 (18.8%)	0 (0.0%)	508 (34.5%)	
**Total amount of follow-up time (days)**	350.0 (336.0, 355.0)	349.0 (336.0, 351.0)	353.0 (343.0, 361.0)	**<0.001**
**Number of fortnightly follow-up visits primary seriesd**	24.0 (21.0, 24.0)	24.0 (23.0, 26.0)	24.0 (19.0, 24.0)	**<0.001**

Table S2.3: *Baseline characteristics at the start of follow up among participants in pooled cohort data by vaccination status*

Variable	Overall, N = 2,707[1]	Vaccination status				p-value[2]
		Unvaccinated, N = 109[1]	Partial, N = 122[1]	Primary series, N = 2,283[1]	Booster, N = 193[1]	
**Age (years)**						**<0.001**
Median (IQR)	35.0 (28.0, 45.0)	36.0 (30.0, 44.0)	29.0 (25.0, 33.0)	35.0 (28.0, 44.0)	42.0 (32.0, 51.0)	
**Age group**						
<30	805 (29.7%)	23 (21.1%)	65 (53.3%)	683 (29.9%)	34 (17.6%)	
30-39	923 (34.1%)	49 (45.0%)	43 (35.2%)	781 (34.2%)	50 (25.9%)	
40-49	591 (21.8%)	20 (18.3%)	9 (7.4%)	507 (22.2%)	55 (28.5%)	
50-59	363 (13.4%)	15 (13.8%)	5 (4.1%)	296 (13.0%)	47 (24.4%)	
60+	25 (0.9%)	2 (1.8%)	0 (0.0%)	16 (0.7%)	7 (3.6%)	
**Sex**						**<0.001**
Female	1,058 (39.1%)	63 (57.8%)	46 (37.7%)	855 (37.5%)	94 (48.7%)	
Male	1,649 (60.9%)	46 (42.2%)	76 (62.3%)	1,428 (62.5%)	99 (51.3%)	
**Highest level of education**						
None	43 (1.6%)	0 (0.0%)	1 (0.8%)	42 (1.8%)	0 (0.0%)	
Primary	268 (9.9%)	10 (9.2%)	16 (13.1%)	214 (9.4%)	28 (14.5%)	
Secondary	1,141 (42.1%)	68 (62.4%)	45 (36.9%)	912 (39.9%)	116 (60.1%)	
University	1,244 (46.0%)	31 (28.4%)	58 (47.5%)	1,106 (48.4%)	49 (25.4%)	
Prefer not to answer	11 (0.4%)	0 (0.0%)	2 (1.6%)	9 (0.4%)	0 (0.0%)	
**Smoking history**						
No, I never smoked	2,425 (89.6%)	94 (86.2%)	113 (92.6%)	2,046 (89.6%)	172 (89.1%)	
I smoke smoking more than one year ago	96 (3.5%)	12 (11.0%)	4 (3.3%)	70 (3.1%)	10 (5.2%)	
Yes, I currently smoke	186 (6.9%)	3 (2.8%)	5 (4.1%)	167 (7.3%)	11 (5.7%)	
**BMI**						
<25	1,066 (39.4%)	24 (22.0%)	55 (45.1%)	936 (41.0%)	51 (26.4%)	
25-29	1,010 (37.3%)	53 (48.6%)	46 (37.7%)	838 (36.7%)	73 (37.8%)	
30-39	584 (21.6%)	30 (27.5%)	18 (14.8%)	472 (20.7%)	64 (33.2%)	
>=40	47 (1.7%)	2 (1.8%)	3 (2.5%)	37 (1.6%)	5 (2.6%)	
**Regularly taking medication**	419 (15.5%)	21 (19.3%)	10 (8.3%)	338 (14.8%)	50 (25.9%)	**<0.001**
(Missing/ Not applicable)	1	0	1	0	0	
**Healthcare worker**						**0.001**
MD	573 (21.2%)	22 (20.2%)	37 (30.3%)	482 (21.1%)	32 (16.6%)	
Nursing/midwifery personnel	977 (36.1%)	57 (52.3%)	39 (32.0%)	797 (34.9%)	84 (43.5%)	
Other	904 (33.4%)	25 (22.9%)	37 (30.3%)	781 (34.2%)	61 (31.6%)	
Paramedical	253 (9.3%)	5 (4.6%)	9 (7.4%)	223 (9.8%)	16 (8.3%)	

Variable	Overall, N = 2,707[1]	Vaccination status<br>Unvaccinated, N = 109[1]	Partial, N = 122[1]	Primary series, N = 2,283[1]	Booster, N = 193[1]	p-value[2]
**Total number of lab-confirmed symptomatic infections during follow-up**						0.14
0	2,541 (93.9%)	106 (97.2%)	111 (91.0%)	2,138 (93.6%)	186 (96.4%)	
1	161 (5.9%)	3 (2.8%)	11 (9.0%)	141 (6.2%)	6 (3.1%)	
2	5 (0.2%)	0 (0.0%)	0 (0.0%)	4 (0.2%)	1 (0.5%)	
**Previous COVID-19 infection**						**<0.001**
0	2,078 (76.8%)	5 (4.6%)	100 (82.0%)	1,833 (80.3%)	140 (72.5%)	
1	629 (23.2%)	104 (95.4%)	22 (18.0%)	450 (19.7%)	53 (27.5%)	
**Vaccination status at end of follow up**						
Unvaccinated	109 (4.0%)	109 (100.0%)	0 (0.0%)	0 (0.0%)	0 (0.0%)	
Partial	109 (4.0%)	0 (0.0%)	109 (89.3%)	0 (0.0%)	0 (0.0%)	
Primary series	2,198 (81.2%)	0 (0.0%)	13 (10.7%)	2,185 (95.7%)	0 (0.0%)	
Booster	291 (10.7%)	0 (0.0%)	0 (0.0%)	98 (4.3%)	193 (100.0%)	
**Type of COVID-19 vaccine (all three doses) at start of follow up**						
AZD1222	387 (14.9%)	0 (0.0%)	31 (25.4%)	332 (14.5%)	24 (12.4%)	
BBIBP-CorV	1,491 (57.4%)	0 (0.0%)	36 (29.5%)	1,413 (61.9%)	42 (21.8%)	
AD5-nCOV	59 (2.3%)	0 (0.0%)	0 (0.0%)	58 (2.5%)	1 (0.5%)	
PiCoVacc	368 (14.2%)	0 (0.0%)	32 (26.2%)	327 (14.3%)	9 (4.7%)	
Gam-Covid-Vac	17 (0.7%)	0 (0.0%)	9 (7.4%)	8 (0.4%)	0 (0.0%)	
Ad26.COV2.S	12 (0.5%)	0 (0.0%)	0 (0.0%)	12 (0.5%)	0 (0.0%)	
mRNA-1273	43 (1.7%)	0 (0.0%)	6 (4.9%)	36 (1.6%)	1 (0.5%)	
BNT162b2	89 (3.4%)	0 (0.0%)	8 (6.6%)	76 (3.3%)	5 (2.6%)	
Heterologous	129 (5.0%)	0 (0.0%)	0 (0.0%)	18 (0.8%)	111 (57.5%)	
Unknown	3 (0.1%)	0 (0.0%)	0 (0.0%)	3 (0.1%)	0 (0.0%)	
(Missing/ Not applicable)	109	109	0	0	0	
**Days between most recent vaccination and illness onset**						
<90	280 (10.8%)	0 (0.0%)	39 (32.0%)	185 (8.1%)	56 (29.0%)	
90-180	408 (15.7%)	0 (0.0%)	25 (20.5%)	323 (14.1%)	60 (31.1%)	
>180	1,910 (73.5%)	0 (0.0%)	58 (47.5%)	1,775 (77.7%)	77 (39.9%)	
(Missing/ Not applicable)	109	109	0	0	0	
**Hospital site**						**<0.001**
Alhussein Hospital	300 (11.1%)	50 (45.9%)	5 (4.1%)	167 (7.3%)	78 (40.4%)	
Alzahraa Hospital	294 (10.9%)	1 (0.9%)	8 (6.6%)	275 (12.0%)	10 (5.2%)	

Variable	Overall, N = 2,707[1]	Vaccination status<br>Unvaccinated, N = 109[1]	Partial, N = 122[1]	Primary series, N = 2,283[1]	Booster, N = 193[1]	p-value[2]
Assiut Hospital	150 (5.5%)	2 (1.8%)	20 (16.4%)	108 (4.7%)	20 (10.4%)	
Bab Alsharia Hospital	290 (10.7%)	49 (45.0%)	8 (6.6%)	184 (8.1%)	49 (25.4%)	
Damiatta Hospital	201 (7.4%)	3 (2.8%)	11 (9.0%)	171 (7.5%)	16 (8.3%)	
Kohat Hospital	395 (14.6%)	3 (2.8%)	20 (16.4%)	371 (16.3%)	1 (0.5%)	
Mardan Hospital	569 (21.0%)	1 (0.9%)	30 (24.6%)	527 (23.1%)	11 (5.7%)	
Swat Hospital	508 (18.8%)	0 (0.0%)	20 (16.4%)	480 (21.0%)	8 (4.1%)	
**Total amount of follow-up time (days)**						**<0.001**
Median (IQR)	350.0 (336.0, 355.0)	336.0 (290.0, 336.0)	351.0 (347.0, 358.0)	351.0 (338.0, 356.0)	336.0 (317.0, 349.0)	
**Number of fortnightly follow-up visits completed**						0.57
Median (IQR)	24.0 (21.0, 24.0)	24.0 (21.0, 25.0)	24.0 (23.0, 24.0)	24.0 (22.0, 24.0)	24.0 (20.0, 26.0)	
**Study country**						**<0.001**
Egypt	1,235 (45.6%)	105 (96.3%)	52 (42.6%)	905 (39.6%)	173 (89.6%)	
Pakistan	1,472 (54.4%)	4 (3.7%)	70 (57.4%)	1,378 (60.4%)	20 (10.4%)	

Table S2.4: *Baseline characteristics at the start of follow up among participants in Egypt's study by vaccination status*

Variable	Overall, N = 1,235[1]	Vaccination status				p-value[2]
		Unvaccinated, N = 105[1]	Partial, N = 52[1]	Primary series, N = 905[1]	Booster, N = 173[1]	
**Age (years)**						<0.001
Median (IQR)	40.0 (30.0, 49.0)	36.0 (31.0, 44.0)	30.5 (27.0, 37.0)	41.0 (31.0, 49.0)	43.0 (34.0, 51.0)	
**Age group**						
<30	264 (21.4%)	20 (19.0%)	23 (44.2%)	191 (21.1%)	30 (17.3%)	
30-39	345 (27.9%)	49 (46.7%)	20 (38.5%)	235 (26.0%)	41 (23.7%)	
40-49	336 (27.2%)	19 (18.1%)	5 (9.6%)	261 (28.8%)	51 (29.5%)	
50-59	265 (21.5%)	15 (14.3%)	4 (7.7%)	202 (22.3%)	44 (25.4%)	
60+	25 (2.0%)	2 (1.9%)	0 (0.0%)	16 (1.8%)	7 (4.0%)	
**Sex**						0.26
Female	688 (55.7%)	61 (58.1%)	23 (44.2%)	513 (56.7%)	91 (52.6%)	
Male	547 (44.3%)	44 (41.9%)	29 (55.8%)	392 (43.3%)	82 (47.4%)	
**Highest level of education**						
None	0 (0.0%)	0 (0.0%)	0 (0.0%)	0 (0.0%)	0 (0.0%)	
Primary	183 (14.8%)	10 (9.5%)	10 (19.2%)	135 (14.9%)	28 (16.2%)	
Secondary	773 (62.6%)	68 (64.8%)	26 (50.0%)	570 (63.0%)	109 (63.0%)	
University	279 (22.6%)	27 (25.7%)	16 (30.8%)	200 (22.1%)	36 (20.8%)	
Prefer not to answer	0 (0.0%)	0 (0.0%)	0 (0.0%)	0 (0.0%)	0 (0.0%)	
**Smoking history**						
No, I never smoked	1,089 (88.2%)	90 (85.7%)	46 (88.5%)	796 (88.0%)	157 (90.8%)	
I smoke smoking more than one year ago	76 (6.2%)	12 (11.4%)	4 (7.7%)	51 (5.6%)	9 (5.2%)	
Yes, I currently smoke	70 (5.7%)	3 (2.9%)	2 (3.8%)	58 (6.4%)	7 (4.0%)	
**BMI**						
<25	270 (21.9%)	21 (20.0%)	12 (23.1%)	199 (22.0%)	38 (22.0%)	
25-29	531 (43.0%)	52 (49.5%)	25 (48.1%)	387 (42.8%)	67 (38.7%)	
30-39	394 (31.9%)	30 (28.6%)	12 (23.1%)	289 (31.9%)	63 (36.4%)	
>=40	40 (3.2%)	2 (1.9%)	3 (5.8%)	30 (3.3%)	5 (2.9%)	
**Regularly taking medication**	302 (24.5%)	21 (20.0%)	6 (11.8%)	228 (25.2%)	47 (27.2%)	0.089
(Missing/ Not applicable)	1	0	1	0	0	
**Healthcare worker**						**<0.001**
MD	156 (12.6%)	18 (17.1%)	15 (28.8%)	101 (11.2%)	22 (12.7%)	
Nursing/midwifery personnel	523 (42.3%)	57 (54.3%)	19 (36.5%)	366 (40.4%)	81 (46.8%)	
Other	436 (35.3%)	25 (23.8%)	15 (28.8%)	342 (37.8%)	54 (31.2%)	
Paramedical	120 (9.7%)	5 (4.8%)	3 (5.8%)	96 (10.6%)	16 (9.2%)	

Variable	Overall, N = 1,235[1]	Vaccination status				p-value[2]
		Unvaccinated, N = 105[1]	Partial, N = 52[1]	Primary series, N = 905[1]	Booster, N = 173[1]	
**Total number of lab-confirmed symptomatic infections during follow-up**	18 (1.5%)	3 (2.9%)	3 (5.8%)	10 (1.1%)	2 (1.2%)	0.033
**Previous COVID-19 infection**						**<0.001**
0	866 (70.1%)	1 (1.0%)	44 (84.6%)	694 (76.7%)	127 (73.4%)	
1	369 (29.9%)	104 (99.0%)	8 (15.4%)	211 (23.3%)	46 (26.6%)	
**Vaccination status at end of follow up**						
Unvaccinated	105 (8.5%)	105 (100.0%)	0 (0.0%)	0 (0.0%)	0 (0.0%)	
Partial	52 (4.2%)	0 (0.0%)	52 (100.0%)	0 (0.0%)	0 (0.0%)	
Primary series	904 (73.2%)	0 (0.0%)	0 (0.0%)	904 (99.9%)	0 (0.0%)	
Booster	174 (14.1%)	0 (0.0%)	0 (0.0%)	1 (0.1%)	173 (100.0%)	
**Type of COVID-19 vaccine (all three doses) at start of follow up**						
AZD1222	375 (33.2%)	0 (0.0%)	26 (50.0%)	325 (35.9%)	24 (13.9%)	
BBIBP-CorV	464 (41.1%)	0 (0.0%)	12 (23.1%)	414 (45.7%)	38 (22.0%)	
AD5-nCOV	0 (0.0%)	0 (0.0%)	0 (0.0%)	0 (0.0%)	0 (0.0%)	
PiCoVacc	81 (7.2%)	0 (0.0%)	6 (11.5%)	66 (7.3%)	9 (5.2%)	
Gam-Covid-Vac	0 (0.0%)	0 (0.0%)	0 (0.0%)	0 (0.0%)	0 (0.0%)	
Ad26.COV2.S	12 (1.1%)	0 (0.0%)	0 (0.0%)	12 (1.3%)	0 (0.0%)	
mRNA-1273	11 (1.0%)	0 (0.0%)	3 (5.8%)	7 (0.8%)	1 (0.6%)	
BNT162b2	81 (7.2%)	0 (0.0%)	5 (9.6%)	71 (7.8%)	5 (2.9%)	
Heterologous	106 (9.4%)	0 (0.0%)	0 (0.0%)	10 (1.1%)	96 (55.5%)	
Unknown	0 (0.0%)	0 (0.0%)	0 (0.0%)	0 (0.0%)	0 (0.0%)	
(Missing/ Not applicable)	105	105	0	0	0	
**Days between most recent vaccination and illness onset**						
<90	71 (6.3%)	0 (0.0%)	2 (3.8%)	13 (1.4%)	56 (32.4%)	
90-180	133 (11.8%)	0 (0.0%)	5 (9.6%)	69 (7.6%)	59 (34.1%)	
>180	926 (81.9%)	0 (0.0%)	45 (86.5%)	823 (90.9%)	58 (33.5%)	
(Missing/ Not applicable)	105	105	0	0	0	
**Hospital site**						**<0.001**
Alhussein Hospital	300 (24.3%)	50 (47.6%)	5 (9.6%)	167 (18.5%)	78 (45.1%)	
Alzahraa Hospital	294 (23.8%)	1 (1.0%)	8 (15.4%)	275 (30.4%)	10 (5.8%)	
Assiut Hospital	150 (12.1%)	2 (1.9%)	20 (38.5%)	108 (11.9%)	20 (11.6%)	
Bab Alsharia Hospital	290 (23.5%)	49 (46.7%)	8 (15.4%)	184 (20.3%)	49 (28.3%)	
Damiatta Hospital	201 (16.3%)	3 (2.9%)	11 (21.2%)	171 (18.9%)	16 (9.2%)	

Variable	Overall, N = 1,235[1]	Vaccination status				p-value[2]
		Unvaccinated, N = 105[1]	Partial, N = 52[1]	Primary series, N = 905[1]	Booster, N = 173[1]	
**Total amount of follow-up time (days)**						**<0.001**
Median (IQR)	349.0 (336.0, 351.0)	336.0 (290.0, 336.0)	350.0 (343.0, 351.0)	350.0 (336.0, 351.0)	336.0 (317.0, 349.0)	
**Number of fortnightly follow-up visits completed**						**<0.001**
Median (IQR)	24.0 (23.0, 26.0)	24.0 (21.0, 25.0)	24.5 (24.0, 26.0)	25.0 (24.0, 26.0)	24.0 (21.0, 26.0)	
**Study country**						
Egypt	1,235 (100.0%)	105 (100.0%)	52 (100.0%)	905 (100.0%)	173 (100.0%)	

Table S2.5: *Baseline characteristics at the start of follow up among participants in Pakistan's study by vaccination status*

Variable	Overall, N = 1,472[1]	Vaccination status				p-value[2]
		Unvaccinated, N = 4[1]	Partial, N = 70[1]	Primary series, N = 1,378[1]	Booster, N = 20[1]	
**Age (years)**						<0.001
Median (IQR)	32.0 (27.0, 39.0)	25.0 (23.5, 30.5)	28.0 (25.0, 32.0)	32.0 (28.0, 39.0)	33.0 (30.0, 42.3)	
**Age group**						
<30	541 (36.8%)	3 (75.0%)	42 (60.0%)	492 (35.7%)	4 (20.0%)	
30-39	578 (39.3%)	0 (0.0%)	23 (32.9%)	546 (39.6%)	9 (45.0%)	
40-49	255 (17.3%)	1 (25.0%)	4 (5.7%)	246 (17.9%)	4 (20.0%)	
50-59	98 (6.7%)	0 (0.0%)	1 (1.4%)	94 (6.8%)	3 (15.0%)	
60+	0 (0.0%)	0 (0.0%)	0 (0.0%)	0 (0.0%)	0 (0.0%)	
**Sex**						0.18
Female	370 (25.1%)	2 (50.0%)	23 (32.9%)	342 (24.8%)	3 (15.0%)	
Male	1,102 (74.9%)	2 (50.0%)	47 (67.1%)	1,036 (75.2%)	17 (85.0%)	
**Highest level of education**						
None	43 (2.9%)	0 (0.0%)	1 (1.4%)	42 (3.0%)	0 (0.0%)	
Primary	85 (5.8%)	0 (0.0%)	6 (8.6%)	79 (5.7%)	0 (0.0%)	
Secondary	368 (25.0%)	0 (0.0%)	19 (27.1%)	342 (24.8%)	7 (35.0%)	
University	965 (65.6%)	4 (100.0%)	42 (60.0%)	906 (65.7%)	13 (65.0%)	
Prefer not to answer	11 (0.7%)	0 (0.0%)	2 (2.9%)	9 (0.7%)	0 (0.0%)	
**Smoking history**						0.15
No, I never smoked	1,336 (90.8%)	4 (100.0%)	67 (95.7%)	1,250 (90.7%)	15 (75.0%)	
I smoke smoking more than one year ago	20 (1.4%)	0 (0.0%)	0 (0.0%)	19 (1.4%)	1 (5.0%)	

Variable	Overall, N = 1,472[1]	Vaccination status Unvaccinated, N = 4[1]	Partial, N = 70[1]	Primary series, N = 1,378[1]	Booster, N = 20[1]	p-value[2]
Yes, I currently smoke	116 (7.9%)	0 (0.0%)	3 (4.3%)	109 (7.9%)	4 (20.0%)	
**BMI**						
<25	796 (54.1%)	3 (75.0%)	43 (61.4%)	737 (53.5%)	13 (65.0%)	
25-29	479 (32.5%)	1 (25.0%)	21 (30.0%)	451 (32.7%)	6 (30.0%)	
30-39	190 (12.9%)	0 (0.0%)	6 (8.6%)	183 (13.3%)	1 (5.0%)	
>=40	7 (0.5%)	0 (0.0%)	0 (0.0%)	7 (0.5%)	0 (0.0%)	
**Regularly taking medication**	117 (7.9%)	0 (0.0%)	4 (5.7%)	110 (8.0%)	3 (15.0%)	0.52
**Healthcare worker**						
MD	417 (28.3%)	4 (100.0%)	22 (31.4%)	381 (27.6%)	10 (50.0%)	
Nursing/midwifery personnel	454 (30.8%)	0 (0.0%)	20 (28.6%)	431 (31.3%)	3 (15.0%)	
Other	468 (31.8%)	0 (0.0%)	22 (31.4%)	439 (31.9%)	7 (35.0%)	
Paramedical	133 (9.0%)	0 (0.0%)	6 (8.6%)	127 (9.2%)	0 (0.0%)	
**Total number of lab-confirmed symptomatic infections during follow-up**						0.10
0	1,324 (89.9%)	4 (100.0%)	62 (88.6%)	1,243 (90.2%)	15 (75.0%)	
1	143 (9.7%)	0 (0.0%)	8 (11.4%)	131 (9.5%)	4 (20.0%)	
2	5 (0.3%)	0 (0.0%)	0 (0.0%)	4 (0.3%)	1 (5.0%)	
**Previous COVID-19 infection**						0.17
0	1,212 (82.3%)	4 (100.0%)	56 (80.0%)	1,139 (82.7%)	13 (65.0%)	
1	260 (17.7%)	0 (0.0%)	14 (20.0%)	239 (17.3%)	7 (35.0%)	
**Vaccination status at end of follow up**						
Unvaccinated	4 (0.3%)	4 (100.0%)	0 (0.0%)	0 (0.0%)	0 (0.0%)	
Partial	57 (3.9%)	0 (0.0%)	57 (81.4%)	0 (0.0%)	0 (0.0%)	
Primary series	1,294 (87.9%)	0 (0.0%)	13 (18.6%)	1,281 (93.0%)	0 (0.0%)	
Booster	117 (7.9%)	0 (0.0%)	0 (0.0%)	97 (7.0%)	20 (100.0%)	
**Type of COVID-19 vaccine (all three doses) at start of follow up**						
AZD1222	12 (0.8%)	0 (0.0%)	5 (7.1%)	7 (0.5%)	0 (0.0%)	
BBIBP-CorV	1,027 (70.0%)	0 (0.0%)	24 (34.3%)	999 (72.5%)	4 (20.0%)	
AD5-nCOV	59 (4.0%)	0 (0.0%)	0 (0.0%)	58 (4.2%)	1 (5.0%)	
PiCoVacc	287 (19.6%)	0 (0.0%)	26 (37.1%)	261 (18.9%)	0 (0.0%)	
Gam-Covid-Vac	17 (1.2%)	0 (0.0%)	9 (12.9%)	8 (0.6%)	0 (0.0%)	
Ad26.COV2.S	0 (0.0%)	0 (0.0%)	0 (0.0%)	0 (0.0%)	0 (0.0%)	
mRNA-1273	32 (2.2%)	0 (0.0%)	3 (4.3%)	29 (2.1%)	0 (0.0%)	
BNT162b2	8 (0.5%)	0 (0.0%)	3 (4.3%)	5 (0.4%)	0 (0.0%)	
Heterologous	23 (1.6%)	0 (0.0%)	0 (0.0%)	8 (0.6%)	15 (75.0%)	
Unknown	3 (0.2%)	0 (0.0%)	0 (0.0%)	3 (0.2%)	0 (0.0%)	

Variable	Overall, N = 1,472[1]	Vaccination status				p-value[2]
		Unvaccinated, N = 4[1]	Partial, N = 70[1]	Primary series, N = 1,378[1]	Booster, N = 20[1]	
(Missing/ Not applicable)	4	4	0	0	0	
**Days between most recent vaccination and illness onset**						<0.001
<90	209 (14.2%)	0 (0.0%)	37 (52.9%)	172 (12.5%)	0 (0.0%)	
90-180	275 (18.7%)	0 (0.0%)	20 (28.6%)	254 (18.4%)	1 (5.0%)	
>180	984 (67.0%)	0 (0.0%)	13 (18.6%)	952 (69.1%)	19 (95.0%)	
(Missing/ Not applicable)	4	4	0	0	0	
**Hospital site**						0.063
Kohat Hospital	395 (26.8%)	3 (75.0%)	20 (28.6%)	371 (26.9%)	1 (5.0%)	
Mardan Hospital	569 (38.7%)	1 (25.0%)	30 (42.9%)	527 (38.2%)	11 (55.0%)	
Swat Hospital	508 (34.5%)	0 (0.0%)	20 (28.6%)	480 (34.8%)	8 (40.0%)	
**Total amount of follow-up time (days)**						<0.001
Median (IQR)	353.0 (343.0, 361.0)	294.5 (239.5, 309.5)	354.0 (347.0, 363.5)	353.0 (344.0, 361.0)	338.0 (326.8, 346.8)	
**Number of fortnightly follow-up visits completed**						0.002
Median (IQR)	24.0 (19.0, 24.0)	20.0 (16.5, 21.0)	24.0 (20.5, 24.0)	24.0 (19.0, 24.0)	22.5 (18.0, 23.0)	
**Study country**						
Pakistan	1,472 (100.0%)	4 (100.0%)	70 (100.0%)	1,378 (100.0%)	20 (100.0%)	

4.2 Missed follow ups

Both the cohort studies included fortnightly follows-ups.

In Egypt's study, 174 participants had at least one partial lost to follow-up, for a total of 1105 missed follow-up points. Complete lost to follow-up before the 26th follow-up (52 weeks = 1 year) were recorded for 453 participants for a total of 3162 missed follow-up points. 283 participants with some sort of complete lost to follow-up, missed the just the last (25th) follow-up

In Pakistan's study, 20 participants had at least one partial lost to follow-up, for a total of 21 missed follow-up points. Complete lost to follow-up before the 26th follow-up (52 weeks = 1 year), were recorded for 1464 participants for a total of 6019 missed follow-up points. Most of the participants (1,072) with some sort of complete lost to follow-up, missed the just the last (25th) follow-up.

4.3 Results for absolute VE in Egypt's cohort study

4.3.1 Subgroup analysis

Absolute VE estimates from Egypt's study overall and by subgroup are shown below in Figure S2.1.

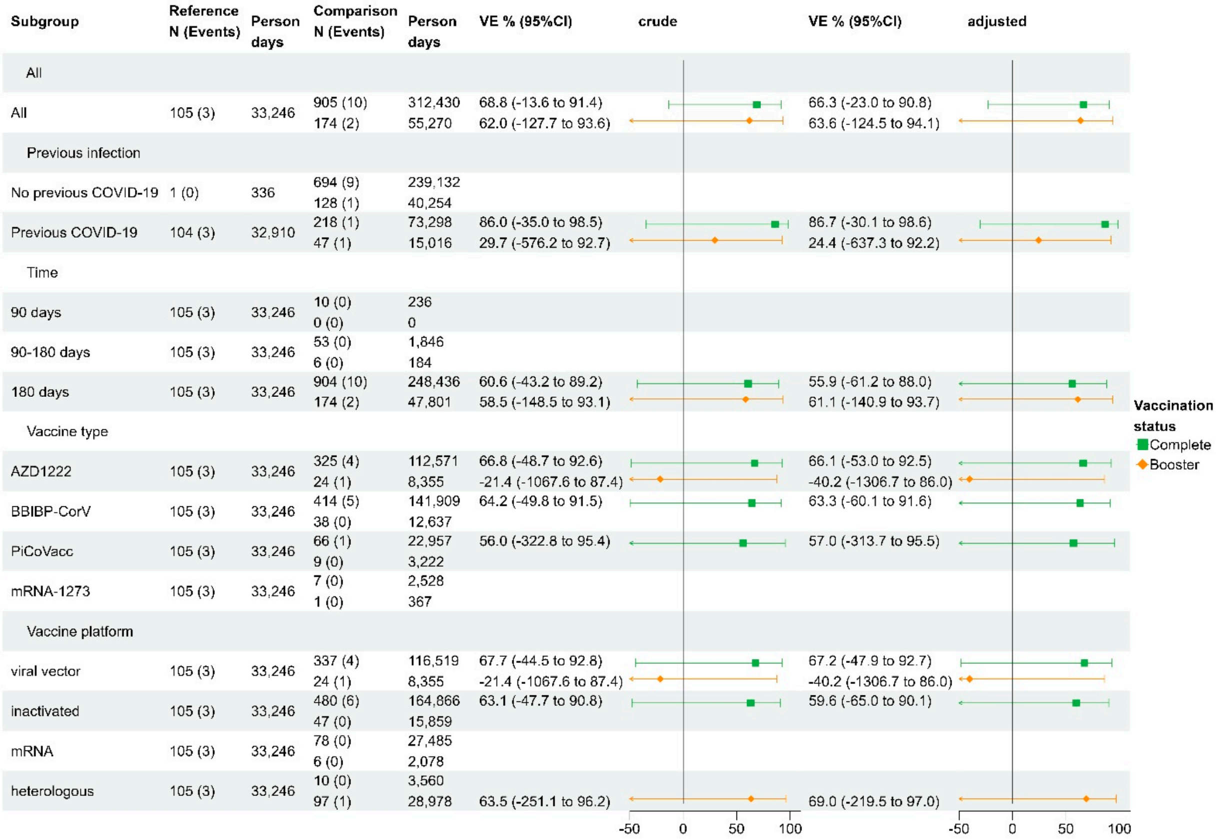

Subgroup	Reference N (Events)	Person days	Comparison N (Events)	Person days	VE % (95%CI) crude	VE % (95%CI) adjusted
**All**						
All	105 (3)	33,246	905 (10)	312,430	68.8 (-13.6 to 91.4)	66.3 (-23.0 to 90.8)
			174 (2)	55,270	62.0 (-127.7 to 93.6)	63.6 (-124.5 to 94.1)
**Previous infection**						
No previous COVID-19	1 (0)	336	694 (9)	239,132		
			128 (1)	40,254		
Previous COVID-19	104 (3)	32,910	218 (1)	73,298	86.0 (-35.0 to 98.5)	86.7 (-30.1 to 98.6)
			47 (1)	15,016	29.7 (-576.2 to 92.7)	24.4 (-637.3 to 92.2)
**Time**						
90 days	105 (3)	33,246	10 (0)	236		
			0 (0)	0		
90-180 days	105 (3)	33,246	53 (0)	1,846		
			6 (0)	184		
180 days	105 (3)	33,246	904 (10)	248,436	60.6 (-43.2 to 89.2)	55.9 (-61.2 to 88.0)
			174 (2)	47,801	58.5 (-148.5 to 93.1)	61.1 (-140.9 to 93.7)
**Vaccine type**						
AZD1222	105 (3)	33,246	325 (4)	112,571	66.8 (-48.7 to 92.6)	66.1 (-53.0 to 92.5)
			24 (1)	8,355	-21.4 (-1067.6 to 87.4)	-40.2 (-1306.7 to 86.0)
BBIBP-CorV	105 (3)	33,246	414 (5)	141,909	64.2 (-49.8 to 91.5)	63.3 (-60.1 to 91.6)
			38 (0)	12,637		
PiCoVacc	105 (3)	33,246	66 (1)	22,957	56.0 (-322.8 to 95.4)	57.0 (-313.7 to 95.5)
			9 (0)	3,222		
mRNA-1273	105 (3)	33,246	7 (0)	2,528		
			1 (0)	367		
**Vaccine platform**						
viral vector	105 (3)	33,246	337 (4)	116,519	67.7 (-44.5 to 92.8)	67.2 (-47.9 to 92.7)
			24 (1)	8,355	-21.4 (-1067.6 to 87.4)	-40.2 (-1306.7 to 86.0)
inactivated	105 (3)	33,246	480 (6)	164,866	63.1 (-47.7 to 90.8)	59.6 (-65.0 to 90.1)
			47 (0)	15,859		
mRNA	105 (3)	33,246	78 (0)	27,485		
			6 (0)	2,078		
heterologous	105 (3)	33,246	10 (0)	3,560		
			97 (1)	28,978	63.5 (-251.1 to 96.2)	69.0 (-219.5 to 97.0)

***Figure S2.1**: aVE results of primary series ('complete') vaccination or booster compared to unvaccinated in Egypt*

4.3.2 Sensitivity analysis

Absolute VE estimates from Egypt's study overall and by subgroup compared for different sensitivity analyses are shown below in Figure S2.2 and Table S2.6.

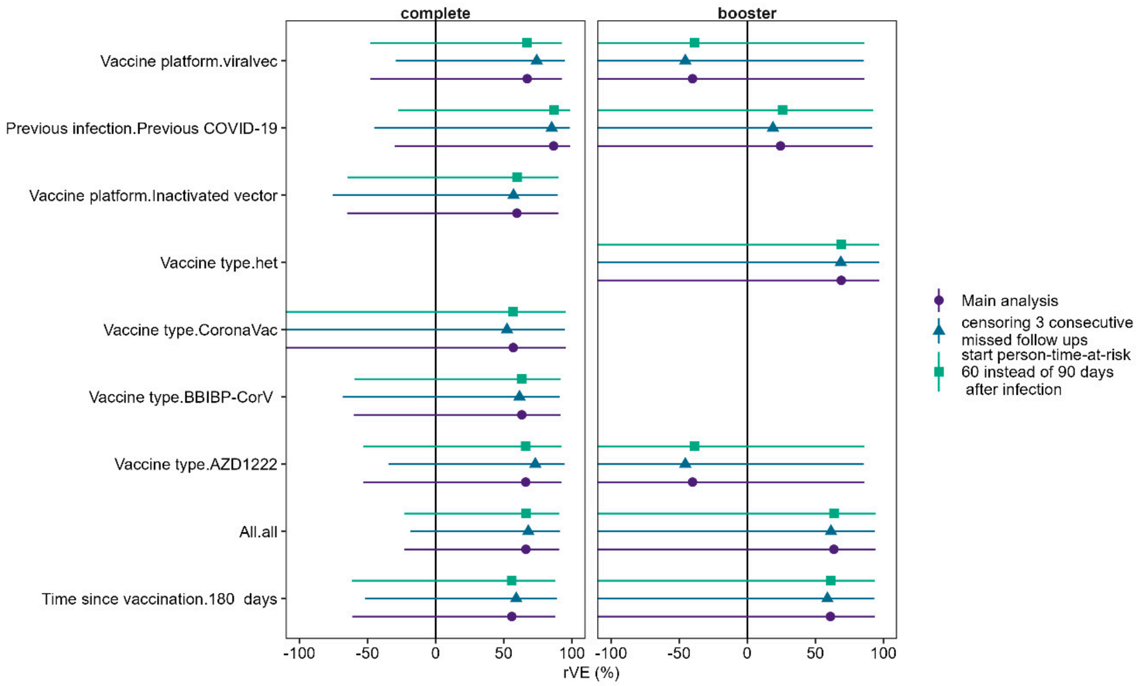

Figure S2.2: *Sensitivity analysis: aVE for partial vaccination, primary series ('complete') and booster compared to unvaccinated in Egypt.*

Table S2.6*: Sensitivity analysis: aVE for partial vaccination, primary series and booster compared to unvaccinated in Egypt.*

Subgroup		Vaccination status	Main analysis	Censoring three or more consequent lost to follow-up	60 days (instead of 90) since previous infection before contributing to person-time at risk
All	all	partial	-125.9 (-1164.2 to 59.6)	-127.7 (-1171.2 to 59.2)	-126.0 (-1165.2 to 59.6)
	all	primary series	66.3 (-23.0 to 90.8)	68.1 (-18.4 to 91.4)	66.3 (-22.9 to 90.8)
	all	booster	63.6 (-124.5 to 94.1)	61.5 (-138.1 to 93.8)	63.7 (-124.0 to 94.1)
Previous infection	Previous COVID-19	partial	-450.9 (-5584.3 to 46.6)	-465.2 (-5661.1 to 44.6)	-446.4 (-5572.1 to 47.4)
	Previous COVID-19	primary series	86.7 (-30.1 to 98.6)	85.2 (-45.1 to 98.5)	86.9 (-27.4 to 98.7)
	Previous COVID-19	booster	24.4 (-637.3 to 92.2)	18.9 (-695.1 to 91.7)	25.8 (-622.7 to 92.4)
Time since vaccination	180 days	partial	-174.5 (-1421.8 to 50.5)	-179.0 (-1443.4 to 49.6)	-174.5 (-1422.5 to 50.5)
	180 days	primary series	55.9 (-61.2 to 88.0)	59.2 (-51.8 to 89.0)	55.8 (-61.5 to 87.9)
	180 days	booster	61.1 (-140.9 to 93.7)	58.9 (-155.1 to 93.4)	61.2 (-140.2 to 93.7)
Vaccine type	AZD1222	partial	-272.0 (-2611.0 to 49.0)	-266.9 (-2549.7 to 49.2)	-273.6 (-2621.3 to 48.7)
	AZD1222	primary series	66.1 (-53.0 to 92.5)	73.2 (-34.7 to 94.7)	66.1 (-53.2 to 92.5)
	AZD1222	booster	-40.2 (-1306.7 to 86.0)	-45.5 (-1373.5 to 85.6)	-38.5 (-1287.8 to 86.2)
	BBIBP-CorV	primary series	63.3 (-60.1 to 91.6)	61.6 (-68.2 to 91.2)	63.4 (-59.6 to 91.6)
	CoronaVac	primary series	57.0 (-313.7 to 95.5)	52.4 (-357.6 to 95.0)	57.0 (-313.9 to 95.5)
	het	booster	69.0 (-219.5 to 97.0)	68.7 (-224.8 to 97.0)	69.0 (-219.4 to 97.0)

Subgroup		Vaccination status	Main analysis	Censoring three or more consequent lost to follow-up	60 days (instead of 90) since previous infection before contributing to person-time at risk
Vaccine platform	Inactivated vector	primary series	59.6 (-65.0 to 90.1)	57.2 (-75.6 to 89.6)	59.8 (-64.6 to 90.2)
	mRNA	partial	-497.7 (-7692.0 to 54.1)	-507.4 (-7744.3 to 53.0)	-493.1 (-7674.9 to 54.8)
	Viral vector	partial	-272.0 (-2611.0 to 49.0)	-266.9 (-2549.7 to 49.2)	-273.6 (-2621.3 to 48.7)
	Viral vector	primary series	67.2 (-47.9 to 92.7)	74.2 (-29.2 to 94.9)	67.2 (-48.1 to 92.7)
	Viral vector	booster	-40.2 (-1306.7 to 86.0)	-45.5 (-1373.5 to 85.6)	-38.5 (-1287.8 to 86.2)

4.4 Subgroup analysis: estimated rVE in cohort studies from Egypt and Pakistan

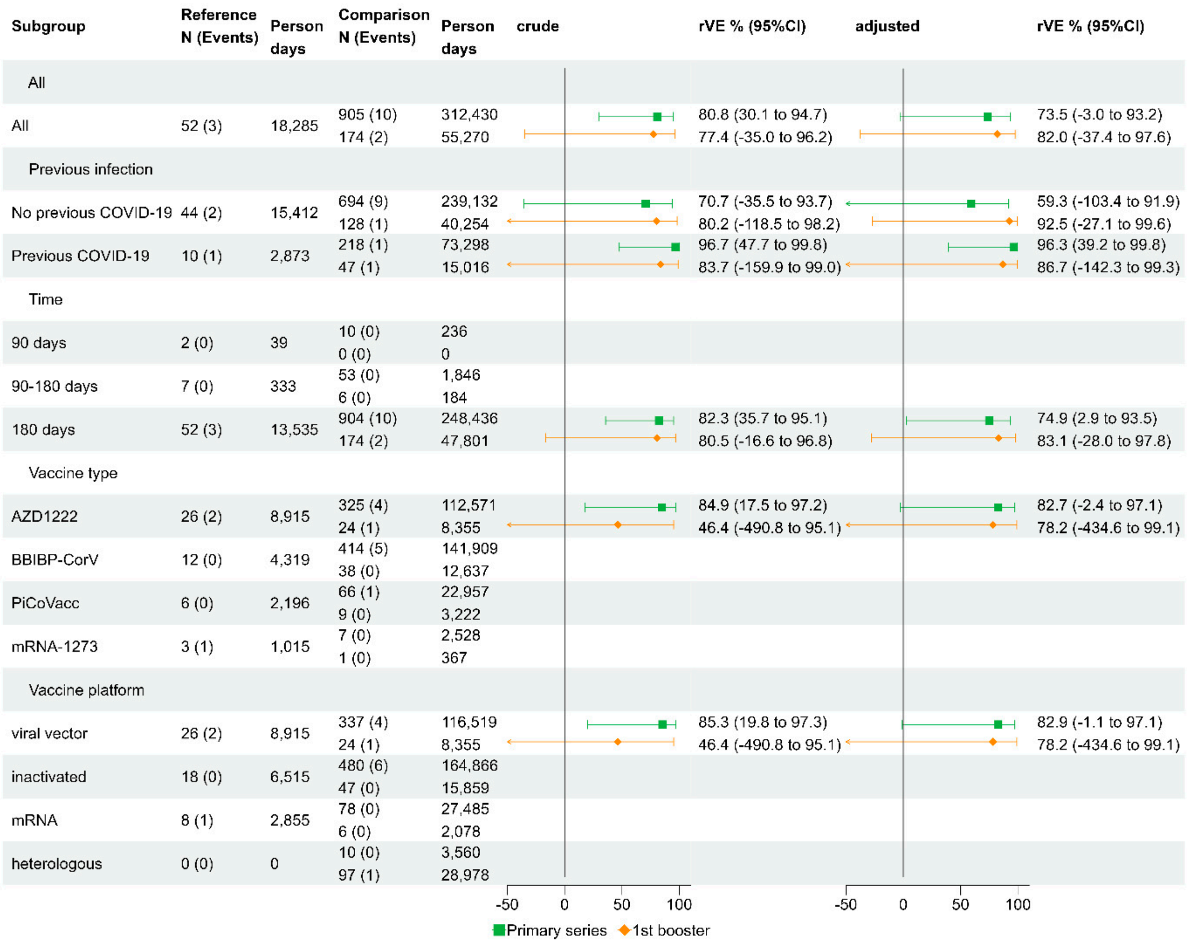

__Figure S2.3__: rVE by vaccination status and subgroups estimated from Egypt's study

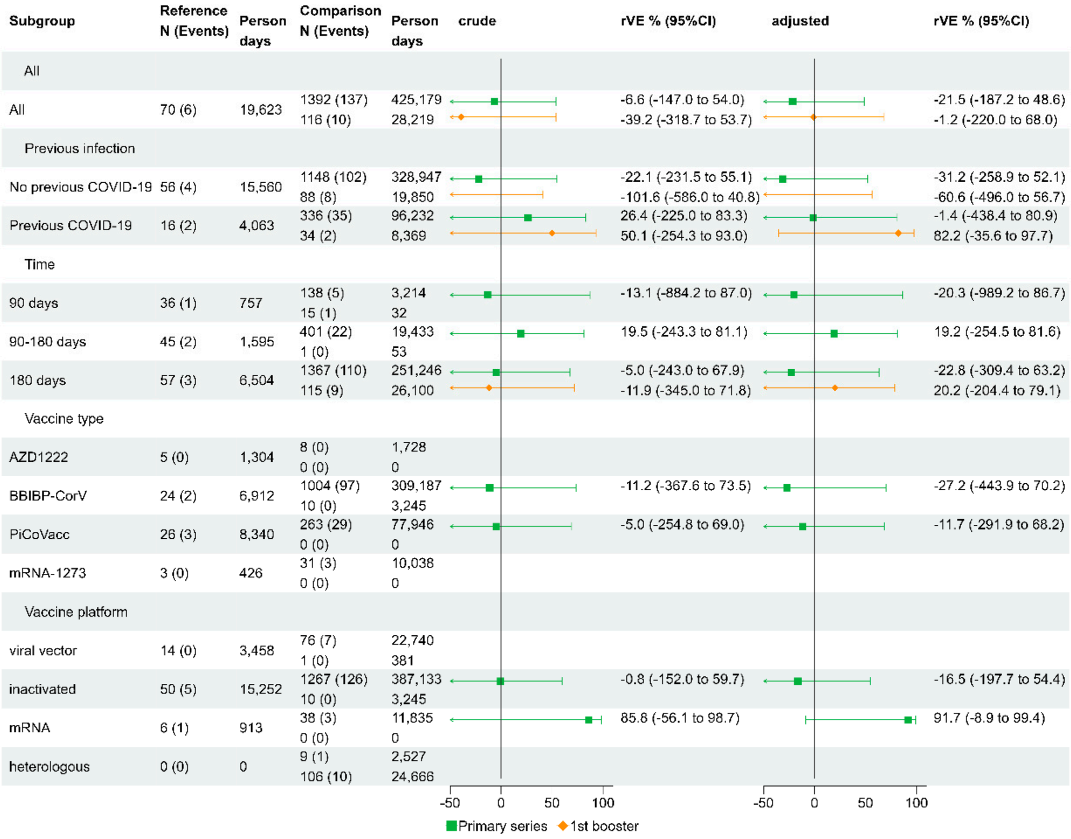

Subgroup	Reference N (Events)	Person days	Comparison N (Events)	Person days	crude	rVE % (95%CI)	adjusted	rVE % (95%CI)
All								
All	70 (6)	19,623	1392 (137)<br>116 (10)	425,179<br>28,219		-6.6 (-147.0 to 54.0)<br>-39.2 (-318.7 to 53.7)		-21.5 (-187.2 to 48.6)<br>-1.2 (-220.0 to 68.0)
Previous infection								
No previous COVID-19	56 (4)	15,560	1148 (102)<br>88 (8)	328,947<br>19,850		-22.1 (-231.5 to 55.1)<br>-101.6 (-586.0 to 40.8)		-31.2 (-258.9 to 52.1)<br>-60.6 (-496.0 to 56.7)
Previous COVID-19	16 (2)	4,063	336 (35)<br>34 (2)	96,232<br>8,369		26.4 (-225.0 to 83.3)<br>50.1 (-254.3 to 93.0)		-1.4 (-438.4 to 80.9)<br>82.2 (-35.6 to 97.7)
Time								
90 days	36 (1)	757	138 (5)<br>15 (1)	3,214<br>32		-13.1 (-884.2 to 87.0)		-20.3 (-989.2 to 86.7)
90-180 days	45 (2)	1,595	401 (22)<br>1 (0)	19,433<br>53		19.5 (-243.3 to 81.1)		19.2 (-254.5 to 81.6)
180 days	57 (3)	6,504	1367 (110)<br>115 (9)	251,246<br>26,100		-5.0 (-243.0 to 67.9)<br>-11.9 (-345.0 to 71.8)		-22.8 (-309.4 to 63.2)<br>20.2 (-204.4 to 79.1)
Vaccine type								
AZD1222	5 (0)	1,304	8 (0)<br>0 (0)	1,728<br>0				
BBIBP-CorV	24 (2)	6,912	1004 (97)<br>10 (0)	309,187<br>3,245		-11.2 (-367.6 to 73.5)		-27.2 (-443.9 to 70.2)
PiCoVacc	26 (3)	8,340	263 (29)<br>0 (0)	77,946<br>0		-5.0 (-254.8 to 69.0)		-11.7 (-291.9 to 68.2)
mRNA-1273	3 (0)	426	31 (3)<br>0 (0)	10,038<br>0				
Vaccine platform								
viral vector	14 (0)	3,458	76 (7)<br>1 (0)	22,740<br>381				
inactivated	50 (5)	15,252	1267 (126)<br>10 (0)	387,133<br>3,245		-0.8 (-152.0 to 59.7)		-16.5 (-197.7 to 54.4)
mRNA	6 (1)	913	38 (3)<br>0 (0)	11,835<br>0		85.8 (-56.1 to 98.7)		91.7 (-8.9 to 99.4)
heterologous	0 (0)	0	9 (1)<br>106 (10)	2,527<br>24,666				

Figure S2.4: *rVE by vaccination status and subgroups estimated from Pakistan's study*

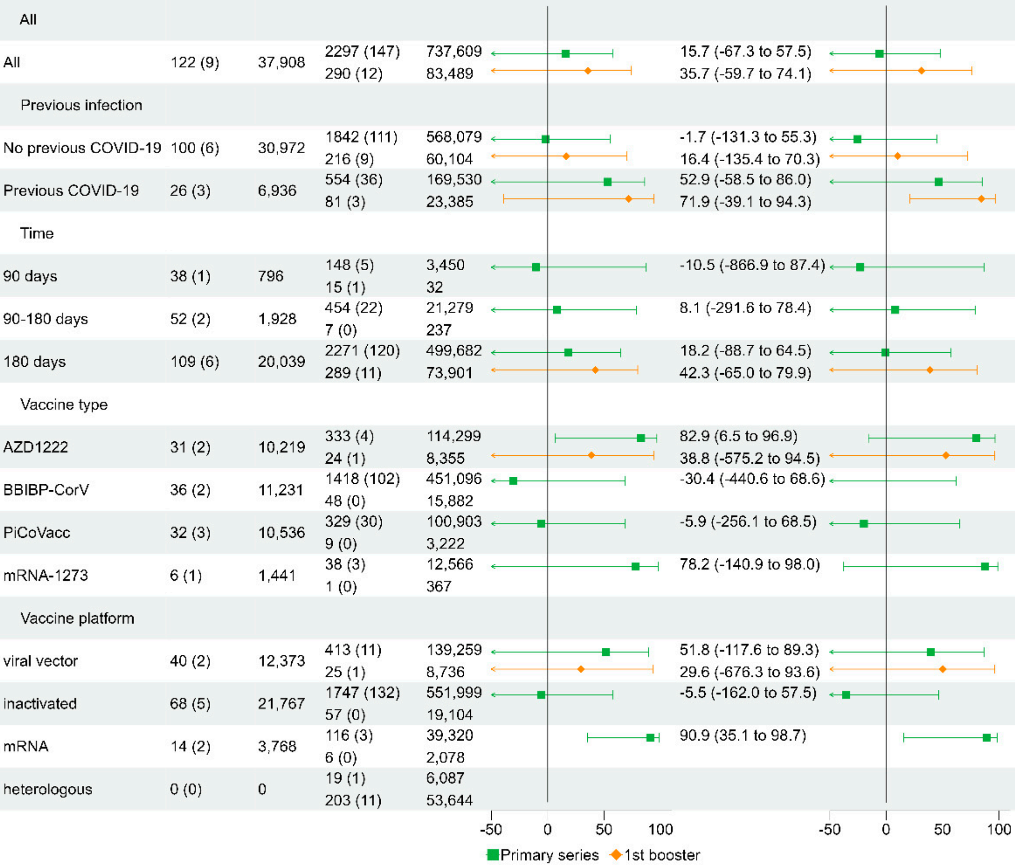

Subgroup	Reference N (Events)	Person days	Comparison N (Events)	Person days	crude rVE % (95%CI)	adjusted rVE % (95%CI)
**All**						
All	122 (9)	37,908	2297 (147)<br>290 (12)	737,609<br>83,489	15.7 (-67.3 to 57.5)<br>35.7 (-59.7 to 74.1)	-5.8 (-114.5 to 47.8)<br>31.2 (-94.6 to 75.7)
**Previous infection**						
No previous COVID-19	100 (6)	30,972	1842 (111)<br>216 (9)	568,079<br>60,104	-1.7 (-131.3 to 55.3)<br>16.4 (-135.4 to 70.3)	-25.7 (-188.2 to 45.2)<br>10.2 (-190.2 to 72.2)
Previous COVID-19	26 (3)	6,936	554 (36)<br>81 (3)	169,530<br>23,385	52.9 (-58.5 to 86.0)<br>71.9 (-39.1 to 94.3)	46.7 (-92.0 to 85.2)<br>84.3 (21.1 to 96.9)
**Time**						
90 days	38 (1)	796	148 (5)<br>15 (1)	3,450<br>32	-10.5 (-866.9 to 87.4)	-22.9 (-1029.4 to 86.6)
90-180 days	52 (2)	1,928	454 (22)<br>7 (0)	21,279<br>237	8.1 (-291.6 to 78.4)	8.1 (-306.7 to 79.2)
180 days	109 (6)	20,039	2271 (120)<br>289 (11)	499,682<br>73,901	18.2 (-88.7 to 64.5)<br>42.3 (-65.0 to 79.9)	-0.5 (-137.2 to 57.4)<br>39.0 (-90.6 to 80.4)
**Vaccine type**						
AZD1222	31 (2)	10,219	333 (4)<br>24 (1)	114,299<br>8,355	82.9 (6.5 to 96.9)<br>38.8 (-575.2 to 94.5)	79.9 (-15.8 to 96.5)<br>53.0 (-485.8 to 96.2)
BBIBP-CorV	36 (2)	11,231	1418 (102)<br>48 (0)	451,096<br>15,882	-30.4 (-440.6 to 68.6)	-60.8 (-585.1 to 62.3)
PiCoVacc	32 (3)	10,536	329 (30)<br>9 (0)	100,903<br>3,222	-5.9 (-256.1 to 68.5)	-19.7 (-308.1 to 64.9)
mRNA-1273	6 (1)	1,441	38 (3)<br>1 (0)	12,566<br>367	78.2 (-140.9 to 98.0)	87.6 (-38.0 to 98.9)
**Vaccine platform**						
viral vector	40 (2)	12,373	413 (11)<br>25 (1)	139,259<br>8,736	51.8 (-117.6 to 89.3)<br>29.6 (-676.3 to 93.6)	39.5 (-178.4 to 86.9)<br>50.3 (-533.5 to 96.1)
inactivated	68 (5)	21,767	1747 (132)<br>57 (0)	551,999<br>19,104	-5.5 (-162.0 to 57.5)	-35.5 (-242.8 to 46.5)
mRNA	14 (2)	3,768	116 (3)<br>6 (0)	39,320<br>2,078	90.9 (35.1 to 98.7)	88.9 (15.3 to 98.6)
heterologous	0 (0)	0	19 (1)<br>203 (11)	6,087<br>53,644		

Figure S2.5: *rVE by vaccination status and subgroups estimated from pooled cohort data*

4.5 Sensitivity analysis: estimated rVE in cohort studies from Egypt and Pakistan

Egypt

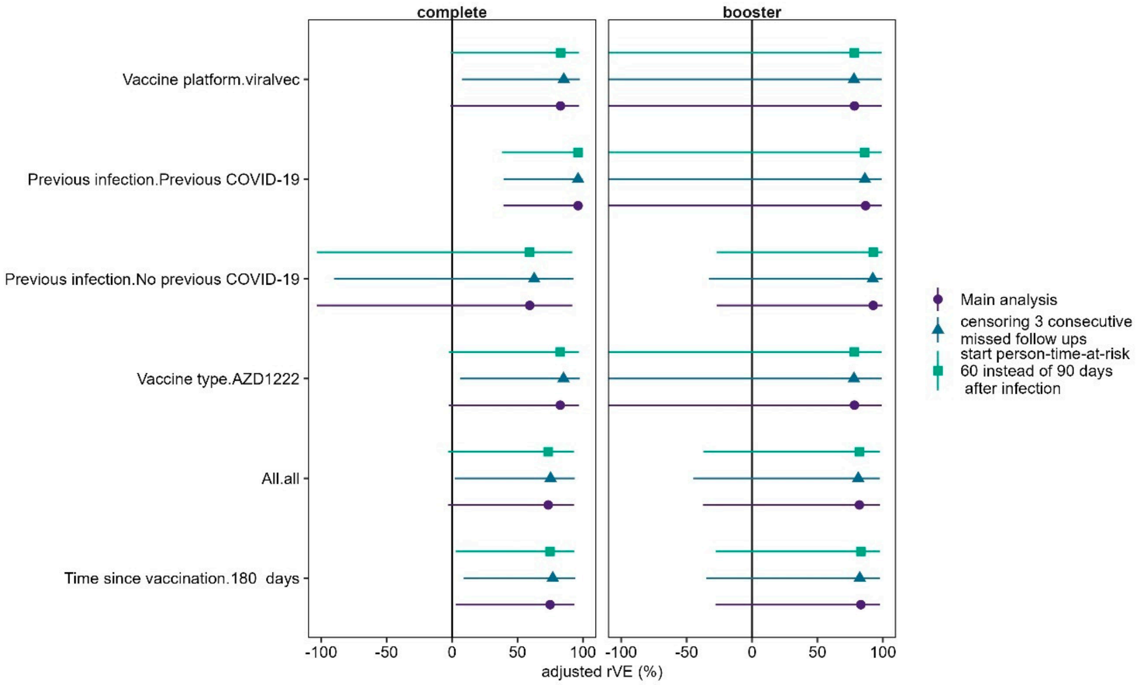

***Figure S2.6**: Sensitivity analysis: rVE for partial vaccination, primary series ('complete') and booster compared to unvaccinated in Egypt.*

***Table S2.7**: Sensitivity analysis with 60 days (instead of 90) since previous infection before contributing to person-time at risk. Summary rVE results of primary series vaccination compared to partial vaccination in Egypt*

**Subgroup**		**Vaccination status**	**Main analysis**	**censoring 3 consecutive missed follow ups**	**start person-time-at-risk 60 instead of 90 days after infection**
All	all	primary series	73.5 (-3.0 to 93.2)	**75.3 (2.1 to 93.8)**	73.5 (-3.0 to 93.2)
	all	booster	82.0 (-37.4 to 97.6)	81.1 (-44.9 to 97.5)	82.0 (-37.3 to 97.6)
Previous infection	No previous COVID-19	primary series	59.3 (-103.4 to 91.9)	62.8 (-90.2 to 92.7)	59.3 (-103.4 to 91.9)
	No previous COVID-19	booster	92.5 (-27.1 to 99.6)	92.2 (-32.9 to 99.5)	92.5 (-27.1 to 99.6)
	**Previous COVID-19**	**primary series**	**96.3 (39.2 to 99.8)**	**96.3 (39.3 to 99.8)**	**96.2 (38.0 to 99.8)**
	Previous COVID-19	booster	86.7 (-142.3 to 99.3)	86.2 (-150.2 to 99.2)	85.9 (-154.8 to 99.2)
Time since vaccination	**180 days**	**primary series**	**74.9 (2.9 to 93.5)**	**77.0 (8.8 to 94.2)**	**74.8 (2.6 to 93.5)**
	180 days	booster	83.1 (-28.0 to 97.8)	82.3 (-35.0 to 97.7)	83.1 (-27.9 to 97.8)
Vaccine platform	Viral vector	primary series	82.9 (-1.1 to 97.1)	**85.4 (7.7 to 97.7)**	82.9 (-1.0 to 97.1)

Subgroup		Vaccination status	Main analysis	censoring 3 consecutive missed follow ups	start person-time-at-risk 60 instead of 90 days after infection
	Viral vector	booster	78.2 (-434.6 to 99.1)	77.9 (-452.3 to 99.1)	78.2 (-434.6 to 99.1)
Vaccine type	AZD1222	primary series	82.7 (-2.4 to 97.1)	**85.2 (6.3 to 97.7)**	82.7 (-2.4 to 97.1)
	AZD1222	booster	78.2 (-434.6 to 99.1)	77.9 (-452.3 to 99.1)	78.2 (-434.6 to 99.1)

Pakistan

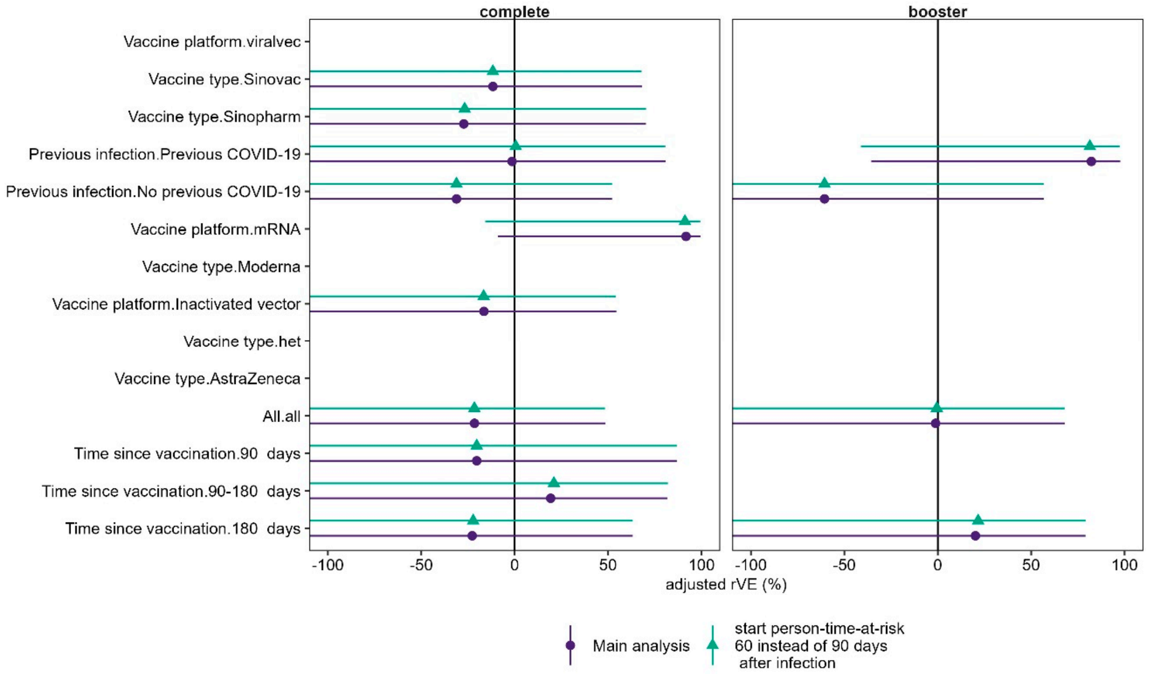

__Figure S2.7__: Sensitivity analysis: rVE for partial vaccination, primary series ('complete') and booster compared to unvaccinated in Pakistan.

__Table S2.8__: Sensitivity analysis with 60 days (instead of 90) since previous infection before contributing to person-time at risk. Summary rVE results of primary series vaccination compared to partial vaccination in Pakistan

Subgroup	Primary series		Booster dose	
	Main analysis	Start person-time-at-risk 60 instead of 90 days after infection	Main analysis	Start person-time-at-risk 60 instead of 90 days after infection
180 days	-22.8 (-309.4 to 63.2)	-22.2 (-305.4 to 63.1)	20.2 (-204.4 to 79.1)	21.6 (-194.6 to 79.1)
90 days	-20.3 (-989.2 to 86.7)	-20.3 (-989.2 to 86.7)	NA	NA
90-180 days	19.2 (-254.5 to 81.6)	21.0 (-246.8 to 82.0)	NA	NA
Inactivated vector	-16.5 (-197.7 to 54.4)	-16.6 (-196.8 to 54.1)	NA	NA
No previous COVID-19	-31.2 (-258.9 to 52.1)	-31.2 (-258.9 to 52.1)	-60.6 (-496.0 to 56.7)	-60.6 (-496.0 to 56.7)
Previous COVID-19	-1.4 (-438.4 to 80.9)	0.5 (-410.3 to 80.6)	82.2 (-35.6 to 97.7)	81.4 (-41.2 to 97.5)
BBIBP-CorV	-27.2 (-443.9 to 70.2)	-26.8 (-440.8 to 70.3)	NA	NA

Subgroup	Primary series		Booster dose	
	Main analysis	Start person-time-at-risk 60 instead of 90 days after infection	Main analysis	Start person-time-at-risk 60 instead of 90 days after infection
PiCoVacc	-11.7 (-291.9 to 68.2)	-11.7 (-289.1 to 67.9)	NA	NA
all	-21.5 (-187.2 to 48.6)	-21.6 (-186.0 to 48.3)	-1.2 (-220.0 to 68.0)	-0.5 (-215.2 to 68.0)
mRNA	91.7 (-8.9 to 99.4)	91.1 (-15.8 to 99.3)	NA	NA

5 Additional results from the TND studies

5.1 Baseline characteristics

Table S2.9: *Adjustment in vaccination status for Iran and Jordan*

		New vaccination status (14 days after last dose)			
Vaccination Status as in data	Number of doses	0	1	2	3
	**IRAN**				
	0	576			
	1	4	139		
	2		8	851	
	3			50	646
	**JORDAN**				
	0	584			
	1		61		
	2		1	957	
	3				140

Table S2.10*: Baseline characteristics of SARI patients included in the TND studies compared across studies and combined*

**Variable**	**Overall**[1]	**Study country** **Iran**, N = 2,274[1]	**Jordan**, N = 1,743[1]	**p-value**[2]
**Test positive**				<0.001
Cases	1,670 (41.6%)	1,448 (63.7%)	222 (12.7%)	
Control	2,347 (58.4%)	826 (36.3%)	1,521 (87.3%)	
**Age (years)**				<0.001
Median (IQR)	60.0 (43.0, 74.0)	63.0 (45.0, 75.0)	58.0 (41.0, 71.0)	
**Age group**				<0.001
12-17	98 (2.4%)	27 (1.2%)	71 (4.1%)	
18-44	958 (23.8%)	521 (22.9%)	437 (25.1%)	
45-64	1,250 (31.1%)	655 (28.8%)	595 (34.1%)	
65+	1,711 (42.6%)	1,071 (47.1%)	640 (36.7%)	
**Sex**				0.003
Female	2,102 (52.3%)	1,237 (54.4%)	865 (49.6%)	
Male	1,915 (47.7%)	1,037 (45.6%)	878 (50.4%)	

Variable	Overall[1]	Study country		p-value[2]
		**Iran**, N = 2,274[1]	**Jordan**, N = 1,743[1]	
**Pregnant**				<0.001
No	2,834 (70.6%)	2,202 (96.8%)	632 (36.3%)	
Not applicable	1,078 (26.8%)	0 (0.0%)	1,078 (61.8%)	
Unknown	1 (0.0%)	0 (0.0%)	1 (0.1%)	
Yes	104 (2.6%)	72 (3.2%)	32 (1.8%)	
**Health care worker**	42 (1.0%)	13 (0.6%)	29 (1.7%)	<0.001
**Smoking history**				<0.001
Never smoked	3,254 (86.3%)	2,206 (97.0%)	1,048 (70.1%)	
Current smoker	515 (13.7%)	68 (3.0%)	447 (29.9%)	
(Missing/ Not applicable)	248	0	248	
**Patient care status**				<0.001
at home, dependent on home support/care	281 (7.0%)	0 (0.0%)	281 (16.1%)	
at home, not dependent on support/care	1,456 (36.2%)	0 (0.0%)	1,456 (83.5%)	
Question not included/Missing	2,280 (56.8%)	2,274 (100.0%)	6 (0.3%)	
**Type of COVID-19 vaccine (all three doses)**				
AZD1222	371 (13.0%)	259 (15.3%)	112 (9.7%)	
BBIBP-CorV	1,714 (60.1%)	1,268 (74.9%)	446 (38.5%)	
BNT162b2	588 (20.6%)	0 (0.0%)	588 (50.7%)	
Gam-Covid-Vac	5 (0.2%)	0 (0.0%)	5 (0.4%)	
Heterologous	175 (6.1%)	167 (9.9%)	8 (0.7%)	
Other	0 (0.0%)	0 (0.0%)	0 (0.0%)	
Unknown	0 (0.0%)	0 (0.0%)	0 (0.0%)	
(Missing/ Not applicable)	1,164	580	584	
**Number of vaccine doses**				<0.001
0	1,164 (29.0%)	580 (25.5%)	584 (33.5%)	
1	209 (5.2%)	147 (6.5%)	62 (3.6%)	
2	1,858 (46.3%)	901 (39.6%)	957 (54.9%)	
3	786 (19.6%)	646 (28.4%)	140 (8.0%)	
**Number of doses (unadjusted)**				<0.001
0	1,160 (28.9%)	576 (25.3%)	584 (33.5%)	
1	204 (5.1%)	143 (6.3%)	61 (3.5%)	
2	1,817 (45.2%)	859 (37.8%)	958 (55.0%)	
3	836 (20.8%)	696 (30.6%)	140 (8.0%)	
**Days since vaccination and illness onset**				<0.001
Median (IQR)	171.1 (81.0, 404.1)	92.0 (58.0, 144.8)	450.0 (343.6, 550.6)	
(Missing/ Not applicable)	1,164	580	584	
**Days since vaccination and illness onset (group)**				<0.001
<90	828 (29.0%)	821 (48.5%)	7 (0.6%)	
90-<180	647 (22.7%)	617 (36.4%)	30 (2.6%)	
>=180	1,378 (48.3%)	256 (15.1%)	1,122 (96.8%)	
(Missing/ Not applicable)	1,164	580	584	
**Days between illness onset and test swab**				<0.001
Median (IQR)	4.0 (3.0, 6.0)	5.0 (3.0, 7.0)	3.0 (2.0, 4.0)	

Variable	Overall[1]	Study country<br>**Iran**, N = 2,274[1]	**Jordan**, N = 1,743[1]	p-value[2]
**Days between illness onset and test swab (group)**				<0.001
<2 days	899 (22.4%)	294 (12.9%)	605 (34.7%)	
>14 days	4 (0.1%)	0 (0.0%)	4 (0.2%)	
2-7 days	2,623 (65.3%)	1,565 (68.8%)	1,058 (60.7%)	
7-14 days	491 (12.2%)	415 (18.2%)	76 (4.4%)	
**Days between illness onset and admission**				<0.001
Median (IQR)	3.0 (2.0, 5.0)	4.0 (3.0, 7.0)	2.0 (1.0, 3.0)	
**Days between illness onset and admission (group)**				<0.001
<2 days	1,624 (40.4%)	366 (16.1%)	1,258 (72.2%)	
2-7 days	2,130 (53.0%)	1,665 (73.2%)	465 (26.7%)	
7-14 days	263 (6.5%)	243 (10.7%)	20 (1.1%)	
**Any underlying health condition**	2,471 (61.5%)	1,058 (46.5%)	1,413 (81.1%)	<0.001
**Number of comorbidities**				<0.001
0	1,571 (39.1%)	1,216 (53.5%)	355 (20.4%)	
1	984 (24.5%)	548 (24.1%)	436 (25.0%)	
2	1,462 (36.4%)	510 (22.4%)	952 (54.6%)	
**Required supplementary oxygen**	2,291 (57.0%)	1,308 (57.5%)	983 (56.4%)	0.48
**Mechanical ventilation**	186 (4.6%)	99 (4.4%)	87 (5.0%)	0.34
**ICU admission**	784 (19.5%)	443 (19.5%)	341 (19.6%)	0.95
**In-hospital death**				0.002
	3 (0.1%)	0 (0.0%)	3 (0.2%)	
No	3,713 (92.4%)	2,126 (93.5%)	1,587 (91.0%)	
Yes	301 (7.5%)	148 (6.5%)	153 (8.8%)	
**Outcome of hospital stay**				0.002
Discharged from hospital	3,713 (92.4%)	2,126 (93.5%)	1,587 (91.0%)	
Transferred	3 (0.1%)	0 (0.0%)	3 (0.2%)	
Died in hospital	301 (7.5%)	148 (6.5%)	153 (8.8%)	
**Study site**				<0.001
Chaharbakht	415 (10.3%)	415 (18.2%)	0 (0.0%)	
Fars	184 (4.6%)	184 (8.1%)	0 (0.0%)	
Gilan	365 (9.1%)	365 (16.1%)	0 (0.0%)	
Hamedan	275 (6.8%)	275 (12.1%)	0 (0.0%)	
Karak Hospital	364 (9.1%)	0 (0.0%)	364 (20.9%)	
Kermanshah	122 (3.0%)	122 (5.4%)	0 (0.0%)	
King Abdullah Hospital	827 (20.6%)	0 (0.0%)	827 (47.4%)	
Kurdistan	149 (3.7%)	149 (6.6%)	0 (0.0%)	
Mazandaran	549 (13.7%)	549 (24.1%)	0 (0.0%)	
Prince Hamza Hospital	305 (7.6%)	0 (0.0%)	305 (17.5%)	
Shahroud	215 (5.4%)	215 (9.5%)	0 (0.0%)	
Zarka Hospital	247 (6.1%)	0 (0.0%)	247 (14.2%)	

Iran

***Table S2.11**: Baseline characteristics of SARI patients included in the TND studies in Iran by case control group*

Variable	N	Overall, N = 2,274[1]	Group		p-value[2]
			Cases, N = 1,448[1]	Control, N = 826[1]	
**Age (years)**	2,274				0.60
Median (IQR)		63.0 (45.0, 75.0)	63.5 (45.0, 76.0)	62.0 (46.0, 75.0)	
**Age group**	2,274				0.29
12-17		27 (1.2%)	14 (1.0%)	13 (1.6%)	
18-44		521 (22.9%)	339 (23.4%)	182 (22.0%)	
45-64		655 (28.8%)	403 (27.8%)	252 (30.5%)	
65+		1,071 (47.1%)	692 (47.8%)	379 (45.9%)	
**Sex**	2,274				0.31
Female		1,237 (54.4%)	776 (53.6%)	461 (55.8%)	
Male		1,037 (45.6%)	672 (46.4%)	365 (44.2%)	
**Pregnant**	2,274				**<0.001**
No		2,202 (96.8%)	1,388 (95.9%)	814 (98.5%)	
Not applicable		0 (0.0%)	0 (0.0%)	0 (0.0%)	
Unknown		0 (0.0%)	0 (0.0%)	0 (0.0%)	
Yes		72 (3.2%)	60 (4.1%)	12 (1.5%)	
**Health care worker**	2,274	13 (0.6%)	10 (0.7%)	3 (0.4%)	0.40
**Smoking history**	2,274				0.27
Never smoked		2,206 (97.0%)	1,409 (97.3%)	797 (96.5%)	
Current smoker		68 (3.0%)	39 (2.7%)	29 (3.5%)	
**Type of COVID-19 vaccine (all three doses)**	1,694				0.93
AZD1222		259 (15.3%)	164 (15.3%)	95 (15.2%)	
BBIBP-CorV		1,268 (74.9%)	799 (74.6%)	469 (75.3%)	
BNT162b2		0 (0.0%)	0 (0.0%)	0 (0.0%)	
Gam-Covid-Vac		0 (0.0%)	0 (0.0%)	0 (0.0%)	
Heterologous		167 (9.9%)	108 (10.1%)	59 (9.5%)	
Other		0 (0.0%)	0 (0.0%)	0 (0.0%)	
Unknown		0 (0.0%)	0 (0.0%)	0 (0.0%)	
(Missing/ Not applicable)		580	377	203	
**Number of vaccine doses**	2,274				0.28
0		580 (25.5%)	377 (26.0%)	203 (24.6%)	
1		147 (6.5%)	83 (5.7%)	64 (7.7%)	
2		901 (39.6%)	573 (39.6%)	328 (39.7%)	
3		646 (28.4%)	415 (28.7%)	231 (28.0%)	
**Number of doses (unadjusted)**	2,274				0.21
0		576 (25.3%)	375 (25.9%)	201 (24.3%)	
1		143 (6.3%)	80 (5.5%)	63 (7.6%)	
2		859 (37.8%)	543 (37.5%)	316 (38.3%)	
3		696 (30.6%)	450 (31.1%)	246 (29.8%)	
**Days since vaccination and illness onset**	1,694				0.19
Median (IQR)		92.0 (58.0, 144.8)	93.1 (60.0, 145.1)	89.0 (54.0, 143.1)	
(Missing/ Not applicable)		580	377	203	
**Days since vaccination and illness onset (group)**	1,694				0.48

**Variable**	**N**	**Overall**, N = 2,274[1]	**Group**		**p-value**[2]
			**Cases**, N = 1,448[1]	**Control**, N = 826[1]	
<90		821 (48.5%)	508 (47.4%)	313 (50.2%)	
90-<180		617 (36.4%)	401 (37.4%)	216 (34.7%)	
>=180		256 (15.1%)	162 (15.1%)	94 (15.1%)	
(Missing/ Not applicable)		580	377	203	
**Days between illness onset and test swab**	2,274				0.005
Median (IQR)		5.0 (3.0, 7.0)	5.0 (3.0, 7.0)	5.0 (3.0, 7.0)	
**Days between illness onset and test swab (group)**	2,274				0.22
<2 days		294 (12.9%)	179 (12.4%)	115 (13.9%)	
>14 days		0 (0.0%)	0 (0.0%)	0 (0.0%)	
2-7 days		1,565 (68.8%)	991 (68.4%)	574 (69.5%)	
7-14 days		415 (18.2%)	278 (19.2%)	137 (16.6%)	
**Days between illness onset and admission**	2,274				**<0.001**
Median (IQR)		4.0 (3.0, 7.0)	5.0 (3.0, 7.0)	4.0 (3.0, 6.0)	
**Days between illness onset and admission (group)**	2,274				**<0.001**
<2 days		366 (16.1%)	198 (13.7%)	168 (20.3%)	
2-7 days		1,665 (73.2%)	1,081 (74.7%)	584 (70.7%)	
7-14 days		243 (10.7%)	169 (11.7%)	74 (9.0%)	
**Any underlying health condition**	2,274	1,058 (46.5%)	682 (47.1%)	376 (45.5%)	0.47
**Number of comorbidities**	2,274				0.53
0		1,216 (53.5%)	766 (52.9%)	450 (54.5%)	
1		548 (24.1%)	360 (24.9%)	188 (22.8%)	
2		510 (22.4%)	322 (22.2%)	188 (22.8%)	
**Required supplementary oxygen**	2,274	1,308 (57.5%)	855 (59.0%)	453 (54.8%)	0.051
**Mechanical ventilation**	2,274	99 (4.4%)	72 (5.0%)	27 (3.3%)	0.056
**ICU admission**	2,274	443 (19.5%)	288 (19.9%)	155 (18.8%)	0.51
**In-hospital death**	2,274				**0.042**
No		2,126 (93.5%)	1,342 (92.7%)	784 (94.9%)	
Yes		148 (6.5%)	106 (7.3%)	42 (5.1%)	
**Outcome of hospital stay**	2,274				**0.042**
Discharged from hospital		2,126 (93.5%)	1,342 (92.7%)	784 (94.9%)	
Transferred		0 (0.0%)	0 (0.0%)	0 (0.0%)	
Died in hospital		148 (6.5%)	106 (7.3%)	42 (5.1%)	
**Study site**	2,274				
Chaharbakht		415 (18.2%)	280 (19.3%)	135 (16.3%)	
Fars		184 (8.1%)	105 (7.3%)	79 (9.6%)	
Gilan		365 (16.1%)	218 (15.1%)	147 (17.8%)	
Hamedan		275 (12.1%)	153 (10.6%)	122 (14.8%)	
Karak Hospital		0 (0.0%)	0 (0.0%)	0 (0.0%)	
Kermanshah		122 (5.4%)	77 (5.3%)	45 (5.4%)	

Variable	N	Overall, N = 2,274[1]	Group		p-value[2]
			**Cases**, N = 1,448[1]	**Control**, N = 826[1]	
King Abdullah Hosp		0 (0.0%)	0 (0.0%)	0 (0.0%)	
Kurdistan		149 (6.6%)	80 (5.5%)	69 (8.4%)	
Mazandaran		549 (24.1%)	376 (26.0%)	173 (20.9%)	
Prince Hamza Hospital		0 (0.0%)	0 (0.0%)	0 (0.0%)	
Shahroud		215 (9.5%)	159 (11.0%)	56 (6.8%)	
Zarka Hospital		0 (0.0%)	0 (0.0%)	0 (0.0%)	

[1]n (%)

[2]Wilcoxon rank sum test; Pearson's Chi-squared test; Fisher's exact test

***Table S2.12**: Baseline characteristics of SARI patients included in the TND studies in Iran by vaccination status*

Variable	Overall, N = 2,274[1]	Vaccination status				p-value[2]
		**Unvaccinated** N = 580[1]	**Partial**, N = 147[1]	**Primary series**, N = 901[1]	**Booster**, N = 646[1]	
**testpositive**						0.28
Cases	1,448 (63.7%)	377 (65.0%)	83 (56.5%)	573 (63.6%)	415 (64.2%)	
Control	826 (36.3%)	203 (35.0%)	64 (43.5%)	328 (36.4%)	231 (35.8%)	
**Age (years)**						**<0.001**
Median (IQR)	63.0 (45.0, 75.0)	56.0 (39.0, 72.0)	60.0 (40.0, 75.0)	58.0 (42.0, 73.0)	70.0 (62.0, 79.0)	
**Age group**						
12-17	27 (1.2%)	8 (1.4%)	2 (1.4%)	17 (1.9%)	0 (0.0%)	
18-44	521 (22.9%)	183 (31.6%)	46 (31.3%)	248 (27.5%)	44 (6.8%)	
45-64	655 (28.8%)	172 (29.7%)	37 (25.2%)	277 (30.7%)	169 (26.2%)	
65+	1,071 (47.1%)	217 (37.4%)	62 (42.2%)	359 (39.8%)	433 (67.0%)	
**Sex**						**0.021**
Female	1,237 (54.4%)	344 (59.3%)	78 (53.1%)	489 (54.3%)	326 (50.5%)	
Male	1,037 (45.6%)	236 (40.7%)	69 (46.9%)	412 (45.7%)	320 (49.5%)	
**Pregnant**						**<0.001**
No	2,202 (96.8%)	549 (94.7%)	142 (96.6%)	867 (96.2%)	644 (99.7%)	
Not applicable	0 (0.0%)	0 (0.0%)	0 (0.0%)	0 (0.0%)	0 (0.0%)	
Unknown	0 (0.0%)	0 (0.0%)	0 (0.0%)	0 (0.0%)	0 (0.0%)	
Yes	72 (3.2%)	31 (5.3%)	5 (3.4%)	34 (3.8%)	2 (0.3%)	
**Health care worker**	13 (0.6%)	2 (0.3%)	1 (0.7%)	8 (0.9%)	2 (0.3%)	0.38
**Smoking history**						0.12
Never smoked	2,206 (97.0%)	568 (97.9%)	143 (97.3%)	877 (97.3%)	618 (95.7%)	
Current smoker	68 (3.0%)	12 (2.1%)	4 (2.7%)	24 (2.7%)	28 (4.3%)	
**Type of COVID-19 vaccine (all three doses)**						
AZD1222	259 (15.3%)	0 (0.0%)	27 (18.4%)	160 (17.8%)	72 (11.1%)	
BBIBP-CorV	1,268 (74.9%)	0 (0.0%)	108 (73.5%)	649 (72.0%)	511 (79.1%)	
BNT162b2	0 (0.0%)	0 (0.0%)	0 (0.0%)	0 (0.0%)	0 (0.0%)	
Gam-Covid-Vac	0 (0.0%)	0 (0.0%)	0 (0.0%)	0 (0.0%)	0 (0.0%)	
Heterologous	167 (9.9%)	0 (0.0%)	12 (8.2%)	92 (10.2%)	63 (9.8%)	
Other	0 (0.0%)	0 (0.0%)	0 (0.0%)	0 (0.0%)	0 (0.0%)	
Unknown	0 (0.0%)	0 (0.0%)	0 (0.0%)	0 (0.0%)	0 (0.0%)	

Variable	Overall, N = 2,274[1]	Vaccination status<br>Unvaccinated N = 580[1]	Partial, N = 147[1]	Primary series, N = 901[1]	Booster, N = 646[1]	p-value[2]
(Missing/ Not applicable)	580	580	0	0	0	
**Number of doses (unadjusted)**						**<0.001**
0	576 (25.3%)	576 (99.3%)	0 (0.0%)	0 (0.0%)	0 (0.0%)	
1	143 (6.3%)	4 (0.7%)	139 (94.6%)	0 (0.0%)	0 (0.0%)	
2	859 (37.8%)	0 (0.0%)	8 (5.4%)	851 (94.5%)	0 (0.0%)	
3	696 (30.6%)	0 (0.0%)	0 (0.0%)	50 (5.5%)	646 (100.0%)	
**Days since vaccination and illness onset**						**<0.001**
Median (IQR)	92.0 (58.0, 144.8)	NA (NA, NA)	125.0 (73.5, 201.6)	122.0 (86.0, 175.1)	57.0 (39.0, 82.0)	
(Missing/ Not applicable)	580	580	0	0	0	
**Days since vaccination and illness onset (group)**						
<90	821 (48.5%)	0 (0.0%)	47 (32.0%)	246 (27.3%)	528 (81.7%)	
90-<180	617 (36.4%)	0 (0.0%)	56 (38.1%)	445 (49.4%)	116 (18.0%)	
>=180	256 (15.1%)	0 (0.0%)	44 (29.9%)	210 (23.3%)	2 (0.3%)	
(Missing/ Not applicable)	580	580	0	0	0	
**Days between illness onset and test swab**						0.28
Median (IQR)	5.0 (3.0, 7.0)	5.0 (3.0, 7.0)	5.0 (3.0, 7.0)	5.0 (3.0, 7.0)	5.0 (3.0, 7.0)	
**Days between illness onset and test swab (group)**						
<2 days	294 (12.9%)	84 (14.5%)	18 (12.2%)	101 (11.2%)	91 (14.1%)	
>14 days	0 (0.0%)	0 (0.0%)	0 (0.0%)	0 (0.0%)	0 (0.0%)	
2-7 days	1,565 (68.8%)	401 (69.1%)	100 (68.0%)	625 (69.4%)	439 (68.0%)	
7-14 days	415 (18.2%)	95 (16.4%)	29 (19.7%)	175 (19.4%)	116 (18.0%)	
**Days between illness onset and admission**						0.55
Median (IQR)	4.0 (3.0, 7.0)	4.0 (3.0, 7.0)	4.0 (3.0, 7.0)	4.0 (3.0, 7.0)	4.0 (3.0, 6.0)	
**Days between illness onset and admission (group)**						0.33
<2 days	366 (16.1%)	107 (18.4%)	20 (13.6%)	128 (14.2%)	111 (17.2%)	
2-7 days	1,665 (73.2%)	407 (70.2%)	111 (75.5%)	676 (75.0%)	471 (72.9%)	
7-14 days	243 (10.7%)	66 (11.4%)	16 (10.9%)	97 (10.8%)	64 (9.9%)	

Variable	Overall, N = 2,274[1]	Vaccination status Unvaccinated N = 580[1]	Partial, N = 147[1]	Primary series, N = 901[1]	Booster, N = 646[1]	p-value[2]
**Any underlying health condition**	1,058 (46.5%)	241 (41.6%)	53 (36.1%)	405 (45.0%)	359 (55.6%)	**<0.001**
**Number of comorbidities**						**<0.001**
0	1,216 (53.5%)	339 (58.4%)	94 (63.9%)	496 (55.0%)	287 (44.4%)	
1	548 (24.1%)	149 (25.7%)	24 (16.3%)	204 (22.6%)	171 (26.5%)	
2	510 (22.4%)	92 (15.9%)	29 (19.7%)	201 (22.3%)	188 (29.1%)	
**Required supplementary oxygen**	1,308 (57.5%)	313 (54.0%)	87 (59.2%)	515 (57.2%)	393 (60.8%)	0.11
**Mechanical ventilation**	99 (4.4%)	31 (5.3%)	5 (3.4%)	34 (3.8%)	29 (4.5%)	0.49
**ICU admission**	443 (19.5%)	96 (16.6%)	31 (21.1%)	176 (19.5%)	140 (21.7%)	0.15
**In-hospital death**						**0.034**
No	2,126 (93.5%)	537 (92.6%)	132 (89.8%)	857 (95.1%)	600 (92.9%)	
Yes	148 (6.5%)	43 (7.4%)	15 (10.2%)	44 (4.9%)	46 (7.1%)	
**Outcome of hospital stay**						**0.034**
Discharged from hospital	2,126 (93.5%)	537 (92.6%)	132 (89.8%)	857 (95.1%)	600 (92.9%)	
Transferred	0 (0.0%)	0 (0.0%)	0 (0.0%)	0 (0.0%)	0 (0.0%)	
Died in hospital	148 (6.5%)	43 (7.4%)	15 (10.2%)	44 (4.9%)	46 (7.1%)	
**Study site**						NA
Chaharbakht	415 (18.2%)	92 (15.9%)	24 (16.3%)	164 (18.2%)	135 (20.9%)	
Fars	184 (8.1%)	130 (22.4%)	12 (8.2%)	31 (3.4%)	11 (1.7%)	
Gilan	365 (16.1%)	71 (12.2%)	27 (18.4%)	167 (18.5%)	100 (15.5%)	
Hamedan	275 (12.1%)	52 (9.0%)	16 (10.9%)	115 (12.8%)	92 (14.2%)	
Karak Hospital	0 (0.0%)	0 (0.0%)	0 (0.0%)	0 (0.0%)	0 (0.0%)	
Kermanshah	122 (5.4%)	28 (4.8%)	9 (6.1%)	47 (5.2%)	38 (5.9%)	
King Abdullah Hospital	0 (0.0%)	0 (0.0%)	0 (0.0%)	0 (0.0%)	0 (0.0%)	
Kurdistan	149 (6.6%)	53 (9.1%)	15 (10.2%)	58 (6.4%)	23 (3.6%)	
Mazandaran	549 (24.1%)	104 (17.9%)	31 (21.1%)	226 (25.1%)	188 (29.1%)	
Prince Hamza Hospital	0 (0.0%)	0 (0.0%)	0 (0.0%)	0 (0.0%)	0 (0.0%)	
Shahroud	215 (9.5%)	50 (8.6%)	13 (8.8%)	93 (10.3%)	59 (9.1%)	
Zarka Hospital	0 (0.0%)	0 (0.0%)	0 (0.0%)	0 (0.0%)	0 (0.0%)	

Jordan

***Table S2.13**: Baseline characteristics of SARI patients included in the TND studies in Jordan by case control group*

Variable	N	Overall, N = 1,743[1]	Group Cases, N = 222[1]	Control, N = 1,521[1]	p-value[2]
**Age (years)**	1,743				**<0.001**
Median (IQR)		58.0 (41.0, 71.0)	64.0 (48.3, 75.8)	57.0 (40.0, 71.0)	
**Age group**	1,743				**<0.001**

Variable	N	Overall, N = 1,743[1]	Group<br>Cases, N = 222[1]	Control, N = 1,521[1]	p-value[2]
12-17		71 (4.1%)	2 (0.9%)	69 (4.5%)	
18-44		437 (25.1%)	46 (20.7%)	391 (25.7%)	
45-64		595 (34.1%)	66 (29.7%)	529 (34.8%)	
65+		640 (36.7%)	108 (48.6%)	532 (35.0%)	
**Sex**	1,743				0.14
Female		865 (49.6%)	100 (45.0%)	765 (50.3%)	
Male		878 (50.4%)	122 (55.0%)	756 (49.7%)	
**Pregnant**	1,743				**0.046**
No		632 (36.3%)	63 (28.4%)	569 (37.4%)	
Not applicable		1,078 (61.8%)	155 (69.8%)	923 (60.7%)	
Unknown		1 (0.1%)	0 (0.0%)	1 (0.1%)	
Yes		32 (1.8%)	4 (1.8%)	28 (1.8%)	
**Health care worker**	1,743	29 (1.7%)	1 (0.5%)	28 (1.8%)	0.16
**Smoking history**	1,495				0.46
Never smoked		1,048 (70.1%)	124 (67.8%)	924 (70.4%)	
Current smoker		447 (29.9%)	59 (32.2%)	388 (29.6%)	
(Missing/ Not applicable)		248	39	209	
**Patient care status**	1,743				**<0.001**
		6 (0.3%)	0 (0.0%)	6 (0.4%)	
at home, dependent on home support/care		281 (16.1%)	59 (26.6%)	222 (14.6%)	
at home, not dependent on support/care		1,456 (83.5%)	163 (73.4%)	1,293 (85.0%)	
**Type of COVID-19 vaccine (all three doses)**	1,159				0.54
AZD1222		112 (9.7%)	17 (12.1%)	95 (9.3%)	
BBIBP-CorV		446 (38.5%)	59 (41.8%)	387 (38.0%)	
BNT162b2		588 (50.7%)	65 (46.1%)	523 (51.4%)	
Gam-Covid-Vac		5 (0.4%)	0 (0.0%)	5 (0.5%)	
Heterologous		8 (0.7%)	0 (0.0%)	8 (0.8%)	
Other		0 (0.0%)	0 (0.0%)	0 (0.0%)	
Unknown		0 (0.0%)	0 (0.0%)	0 (0.0%)	
(Missing/ Not applicable)		584	81	503	
**Number of vaccine doses**	1,743				0.20
0		584 (33.5%)	81 (36.5%)	503 (33.1%)	
1		62 (3.6%)	8 (3.6%)	54 (3.6%)	
2		957 (54.9%)	109 (49.1%)	848 (55.8%)	
3		140 (8.0%)	24 (10.8%)	116 (7.6%)	

Variable	N	Overall, N = 1,743[1]	Group<br>Cases, N = 222[1]	Control, N = 1,521[1]	p-value[2]
**Number of doses (unadjusted)**	1,743				0.21
0		584 (33.5%)	81 (36.5%)	503 (33.1%)	
1		61 (3.5%)	7 (3.2%)	54 (3.6%)	
2		958 (55.0%)	110 (49.5%)	848 (55.8%)	
3		140 (8.0%)	24 (10.8%)	116 (7.6%)	
**Days since vaccination and illness onset**	1,159				0.20
Median (IQR)		450.0 (343.6, 550.6)	422.1 (322.1, 549.1)	453.1 (346.3, 550.8)	
(Missing/ Not applicable)		584	81	503	
**Days since vaccination and illness onset (group)**	1,159				0.37
<90		7 (0.6%)	0 (0.0%)	7 (0.7%)	
90-<180		30 (2.6%)	6 (4.3%)	24 (2.4%)	
>=180		1,122 (96.8%)	135 (95.7%)	987 (97.0%)	
(Missing/ Not applicable)		584	81	503	
**Days between illness onset and test swab**	1,743				0.95
Median (IQR)		3.0 (2.0, 4.0)	3.0 (2.0, 4.0)	3.0 (2.0, 4.0)	
**Days between illness onset and test swab (group)**	1,743				0.83
<2 days		605 (34.7%)	81 (36.5%)	524 (34.5%)	
>14 days		4 (0.2%)	0 (0.0%)	4 (0.3%)	
2-7 days		1,058 (60.7%)	130 (58.6%)	928 (61.0%)	
7-14 days		76 (4.4%)	11 (5.0%)	65 (4.3%)	
**Days between illness onset and admission**	1,743				0.54
Median (IQR)		2.0 (1.0, 3.0)	2.0 (1.0, 3.0)	2.0 (1.0, 3.0)	
**Days between illness onset and admission (group)**	1,743				0.72
<2 days		1,258 (72.2%)	161 (72.5%)	1,097 (72.1%)	
2-7 days		465 (26.7%)	60 (27.0%)	405 (26.6%)	
7-14 days		20 (1.1%)	1 (0.5%)	19 (1.2%)	
**Any underlying health condition**	1,743	1,413 (81.1%)	185 (83.3%)	1,228 (80.7%)	0.36
**Number of comorbidities**	1,743				0.14
0		355 (20.4%)	40 (18.0%)	315 (20.7%)	

Variable	N	Overall, N = 1,743[1]	Group<br>Cases, N = 222[1]	 Control, N = 1,521[1]	p-value[2]
1		436 (25.0%)	47 (21.2%)	389 (25.6%)	
2		952 (54.6%)	135 (60.8%)	817 (53.7%)	
**Required supplementary oxygen**	1,743	983 (56.4%)	121 (54.5%)	862 (56.7%)	0.54
**Mechanical ventilation**	1,743	87 (5.0%)	18 (8.1%)	69 (4.5%)	0.022
**ICU admission**	1,743	341 (19.6%)	47 (21.2%)	294 (19.3%)	0.52
**In-hospital death**	1,743				**0.017**
		3 (0.2%)	0 (0.0%)	3 (0.2%)	
No		1,587 (91.0%)	191 (86.0%)	1,396 (91.8%)	
Yes		153 (8.8%)	31 (14.0%)	122 (8.0%)	
**Outcome of hospital stay**	1,743				0.017
Discharged from hospital		1,587 (91.0%)	191 (86.0%)	1,396 (91.8%)	
Transferred		3 (0.2%)	0 (0.0%)	3 (0.2%)	
Died in hospital		153 (8.8%)	31 (14.0%)	122 (8.0%)	
**Study site**	1,743				**<0.001**
Chaharbakht		0 (0.0%)	0 (0.0%)	0 (0.0%)	
Fars		0 (0.0%)	0 (0.0%)	0 (0.0%)	
Gilan		0 (0.0%)	0 (0.0%)	0 (0.0%)	
Hamedan		0 (0.0%)	0 (0.0%)	0 (0.0%)	
Karak Hospital		364 (20.9%)	37 (16.7%)	327 (21.5%)	
Kermanshah		0 (0.0%)	0 (0.0%)	0 (0.0%)	
King Abdullah Hospital		827 (47.4%)	114 (51.4%)	713 (46.9%)	
Kurdistan		0 (0.0%)	0 (0.0%)	0 (0.0%)	
Mazandaran		0 (0.0%)	0 (0.0%)	0 (0.0%)	
Prince Hamza Hospital		305 (17.5%)	61 (27.5%)	244 (16.0%)	
Shahroud		0 (0.0%)	0 (0.0%)	0 (0.0%)	
Zarka Hospital		247 (14.2%)	10 (4.5%)	237 (15.6%)	

[1]n (%)

[2]Wilcoxon rank sum test; Pearson's Chi-squared test; Fisher's exact test

Table S2.14: Baseline characteristics of SARI patients included in the TND studies in Jordan by vaccination status

Variable	Overall, N = 1,743[1]	Vaccination status Unvaccinated, N = 584[1]	Partial, N = 62[1]	Primary series, N = 957[1]	Booster, N = 140[1]	p-value[2]
**testpositive**						0.20
Cases	222 (12.7%)	81 (13.9%)	8 (12.9%)	109 (11.4%)	24 (17.1%)	
Control	1,521 (87.3%)	503 (86.1%)	54 (87.1%)	848 (88.6%)	116 (82.9%)	
**Age (years)**						**<0.001**
Median (IQR)	58.0 (41.0, 71.0)	60.0 (36.8, 76.0)	54.5 (39.0, 69.0)	55.0 (41.0, 69.0)	64.0 (54.0, 75.0)	
**Age group**						
12-17	71 (4.1%)	69 (11.8%)	0 (0.0%)	2 (0.2%)	0 (0.0%)	
18-44	437 (25.1%)	113 (19.3%)	21 (33.9%)	286 (29.9%)	17 (12.1%)	
45-64	595 (34.1%)	145 (24.8%)	21 (33.9%)	372 (38.9%)	57 (40.7%)	
65+	640 (36.7%)	257 (44.0%)	20 (32.3%)	297 (31.0%)	66 (47.1%)	
**Sex**						**<0.001**
Female	865 (49.6%)	344 (58.9%)	37 (59.7%)	444 (46.4%)	40 (28.6%)	
Male	878 (50.4%)	240 (41.1%)	25 (40.3%)	513 (53.6%)	100 (71.4%)	
**Pregnant**						
No	632 (36.3%)	225 (38.5%)	26 (41.9%)	349 (36.5%)	32 (22.9%)	
Not applicable	1,078 (61.8%)	350 (59.9%)	34 (54.8%)	590 (61.7%)	104 (74.3%)	
Unknown	1 (0.1%)	1 (0.2%)	0 (0.0%)	0 (0.0%)	0 (0.0%)	
Yes	32 (1.8%)	8 (1.4%)	2 (3.2%)	18 (1.9%)	4 (2.9%)	
**Health care worker**	29 (1.7%)	1 (0.2%)	0 (0.0%)	25 (2.6%)	3 (2.1%)	**<0.001**
**Smoking history**						**<0.001**
Never smoked	1,048 (70.1%)	407 (79.8%)	40 (72.7%)	531 (64.8%)	70 (63.1%)	
Current smoker	447 (29.9%)	103 (20.2%)	15 (27.3%)	288 (35.2%)	41 (36.9%)	
(Missing/ Not applicable)	248	74	7	138	29	
**Patient care status**						
	6 (0.3%)	0 (0.0%)	0 (0.0%)	5 (0.5%)	1 (0.7%)	
at home, dependent on home support/care	281 (16.1%)	134 (22.9%)	6 (9.7%)	111 (11.6%)	30 (21.4%)	
at home, not dependent on support/care	1,456 (83.5%)	450 (77.1%)	56 (90.3%)	841 (87.9%)	109 (77.9%)	
**Type of COVID-19 vaccine (all three doses)**						NA
AZD1222	112 (9.7%)	0 (0.0%)	3 (4.8%)	97 (10.1%)	12 (8.6%)	
BBIBP-CorV	446 (38.5%)	0 (0.0%)	14 (22.6%)	372 (38.9%)	60 (42.9%)	
BNT162b2	588 (50.7%)	0 (0.0%)	45 (72.6%)	476 (49.7%)	67 (47.9%)	
Gam-Covid-Vac	5 (0.4%)	0 (0.0%)	0 (0.0%)	4 (0.4%)	1 (0.7%)	
Heterologous	8 (0.7%)	0 (0.0%)	0 (0.0%)	8 (0.8%)	0 (0.0%)	
Other	0 (0.0%)	0 (0.0%)	0 (0.0%)	0 (0.0%)	0 (0.0%)	
Unknown	0 (0.0%)	0 (0.0%)	0 (0.0%)	0 (0.0%)	0 (0.0%)	
(Missing/ Not applicable)	584	584	0	0	0	
**Number of doses (unadjusted)**						

Variable	Overall, N = 1,743[1]	Vaccination status<br>Unvaccinated, N = 584[1]	Partial, N = 62[1]	Primary series, N = 957[1]	Booster, N = 140[1]	p-value[2]
0	584 (33.5%)	584 (100.0%)	0 (0.0%)	0 (0.0%)	0 (0.0%)	
1	61 (3.5%)	0 (0.0%)	61 (98.4%)	0 (0.0%)	0 (0.0%)	
2	958 (55.0%)	0 (0.0%)	1 (1.6%)	957 (100.0%)	0 (0.0%)	
3	140 (8.0%)	0 (0.0%)	0 (0.0%)	0 (0.0%)	140 (100.0%)	
**Days since vaccination and illness onset**						**<0.001**
Median (IQR)	450.0 (343.6, 550.6)	NA (NA, NA)	385.6 (278.5, 475.8)	470.0 (383.0, 572.1)	327.5 (240.8, 388.6)	
(Missing/ Not applicable)	584	584	0	0	0	
**Days since vaccination and illness onset (group)**						**<0.001**
<90	7 (0.6%)	0 (NA%)	0 (0.0%)	4 (0.4%)	3 (2.1%)	
90-<180	30 (2.6%)	0 (NA%)	3 (4.8%)	14 (1.5%)	13 (9.3%)	
>=180	1,122 (96.8%)	0 (NA%)	59 (95.2%)	939 (98.1%)	124 (88.6%)	
(Missing/ Not applicable)	584	584	0	0	0	
**Days between illness onset and test swab**						0.27
Median (IQR)	3.0 (2.0, 4.0)	3.0 (2.0, 4.0)	3.0 (2.0, 4.0)	3.0 (2.0, 4.0)	3.0 (2.0, 4.0)	
**Days between illness onset and test swab (group)**						
<2 days	605 (34.7%)	196 (33.6%)	22 (35.5%)	324 (33.9%)	63 (45.0%)	
>14 days	4 (0.2%)	0 (0.0%)	0 (0.0%)	4 (0.4%)	0 (0.0%)	
2-7 days	1,058 (60.7%)	363 (62.2%)	38 (61.3%)	585 (61.1%)	72 (51.4%)	
7-14 days	76 (4.4%)	25 (4.3%)	2 (3.2%)	44 (4.6%)	5 (3.6%)	
**Days between illness onset and admission**						0.54
Median (IQR)	2.0 (1.0, 3.0)	2.0 (1.0, 3.0)	2.0 (1.0, 3.0)	2.0 (1.0, 3.0)	2.0 (1.0, 3.0)	
**Days between illness onset and admission (group)**						
<2 days	1,258 (72.2%)	428 (73.3%)	43 (69.4%)	684 (71.5%)	103 (73.6%)	
2-7 days	465 (26.7%)	148 (25.3%)	19 (30.6%)	261 (27.3%)	37 (26.4%)	
7-14 days	20 (1.1%)	8 (1.4%)	0 (0.0%)	12 (1.3%)	0 (0.0%)	
**Any underlying health condition**	1,413 (81.1%)	481 (82.4%)	50 (80.6%)	761 (79.5%)	121 (86.4%)	0.19
**Number of comorbidities**						**0.017**
0	355 (20.4%)	116 (19.9%)	13 (21.0%)	206 (21.5%)	20 (14.3%)	
1	436 (25.0%)	159 (27.2%)	20 (32.3%)	233 (24.3%)	24 (17.1%)	

Variable	Overall, N = 1,743[1]	Vaccination status<br>Unvaccinated, N = 584[1]	Partial, N = 62[1]	Primary series, N = 957[1]	Booster, N = 140[1]	p-value[2]
2	952 (54.6%)	309 (52.9%)	29 (46.8%)	518 (54.1%)	96 (68.6%)	
**Required supplementary oxygen**	983 (56.4%)	373 (63.9%)	32 (51.6%)	506 (52.9%)	72 (51.4%)	**<0.001**
**Mechanical ventilation**	87 (5.0%)	34 (5.8%)	1 (1.6%)	44 (4.6%)	8 (5.7%)	0.46
**ICU admission**	341 (19.6%)	123 (21.1%)	13 (21.0%)	173 (18.1%)	32 (22.9%)	0.36
**In-hospital death**						**0.036**
	3 (0.2%)	1 (0.2%)	0 (0.0%)	2 (0.2%)	0 (0.0%)	
No	1,587 (91.0%)	517 (88.5%)	57 (91.9%)	890 (93.0%)	123 (87.9%)	
Yes	153 (8.8%)	66 (11.3%)	5 (8.1%)	65 (6.8%)	17 (12.1%)	
**Outcome of hospital stay**						**0.036**
Discharged from hospital	1,587 (91.0%)	517 (88.5%)	57 (91.9%)	890 (93.0%)	123 (87.9%)	
Transferred	3 (0.2%)	1 (0.2%)	0 (0.0%)	2 (0.2%)	0 (0.0%)	
Died in hospital	153 (8.8%)	66 (11.3%)	5 (8.1%)	65 (6.8%)	17 (12.1%)	
**Study site**						
Chaharbakht	0 (0.0%)	0 (0.0%)	0 (0.0%)	0 (0.0%)	0 (0.0%)	
Fars	0 (0.0%)	0 (0.0%)	0 (0.0%)	0 (0.0%)	0 (0.0%)	
Gilan	0 (0.0%)	0 (0.0%)	0 (0.0%)	0 (0.0%)	0 (0.0%)	
Hamedan	0 (0.0%)	0 (0.0%)	0 (0.0%)	0 (0.0%)	0 (0.0%)	
Karak Hospital	364 (20.9%)	149 (25.5%)	14 (22.6%)	188 (19.6%)	13 (9.3%)	
Kermanshah	0 (0.0%)	0 (0.0%)	0 (0.0%)	0 (0.0%)	0 (0.0%)	
King Abdullah Hosp	827 (47.4%)	256 (43.8%)	32 (51.6%)	471 (49.2%)	68 (48.6%)	
Kurdistan	0 (0.0%)	0 (0.0%)	0 (0.0%)	0 (0.0%)	0 (0.0%)	
Mazandaran	0 (0.0%)	0 (0.0%)	0 (0.0%)	0 (0.0%)	0 (0.0%)	
Prince Hamza Hospital	305 (17.5%)	79 (13.5%)	13 (21.0%)	167 (17.5%)	46 (32.9%)	
Shahroud	0 (0.0%)	0 (0.0%)	0 (0.0%)	0 (0.0%)	0 (0.0%)	
Zarka Hospital	247 (14.2%)	100 (17.1%)	3 (4.8%)	131 (13.7%)	13 (9.3%)	

Pooled

***Table S2.15**: Baseline characteristics of SARI patients included in the TND studies in Jordan by case control group*

Variable	**Overall**, N = 4,017[1]	Group<br>**Cases**, N = 1,670[1]	**Control**, N = 2,347[1]	p-value[2]
**Age (years)**				**<0.001**
Median (IQR)	60.0 (43.0, 74.0)	64.0 (46.0, 76.0)	59.0 (42.0, 72.0)	
**Age group**				**<0.001**
12-17	98 (2.4%)	16 (1.0%)	82 (3.5%)	
18-44	958 (23.8%)	385 (23.1%)	573 (24.4%)	
45-64	1,250 (31.1%)	469 (28.1%)	781 (33.3%)	
65+	1,711 (42.6%)	800 (47.9%)	911 (38.8%)	
**Sex**				0.89
Female	2,102 (52.3%)	876 (52.5%)	1,226 (52.2%)	
Male	1,915 (47.7%)	794 (47.5%)	1,121 (47.8%)	

Variable	Overall, N = 4,017[1]	Group		p-value[2]
		Cases, N = 1,670[1]	Control, N = 2,347[1]	
**Pregnant**				**<0.001**
No	2,834 (70.6%)	1,451 (86.9%)	1,383 (58.9%)	
Not applicable	1,078 (26.8%)	155 (9.3%)	923 (39.3%)	
Unknown	1 (0.0%)	0 (0.0%)	1 (0.0%)	
Yes	104 (2.6%)	64 (3.8%)	40 (1.7%)	
**Health care worker**	42 (1.0%)	11 (0.7%)	31 (1.3%)	**0.042**
**Smoking history**				**<0.001**
Never smoked	3,254 (86.3%)	1,533 (94.0%)	1,721 (80.5%)	
Current smoker	515 (13.7%)	98 (6.0%)	417 (19.5%)	
(Missing/ Not applicable)	248	39	209	
**Patient care status**				**<0.001**
Not asked	2,280 (56.8%)	1,448 (86.7%)	832 (35.4%)	
at home, dependent on home support/care	281 (7.0%)	59 (3.5%)	222 (9.5%)	
at home, not dependent on support/care	1,456 (36.2%)	163 (9.8%)	1,293 (55.1%)	
**Type of COVID-19 vaccine (all three doses)**				NA
AZD1222	371 (13.0%)	181 (14.9%)	190 (11.6%)	
BBIBP-CorV	1,714 (60.1%)	858 (70.8%)	856 (52.2%)	
BNT162b2	588 (20.6%)	65 (5.4%)	523 (31.9%)	
Gam-Covid-Vac	5 (0.2%)	0 (0.0%)	5 (0.3%)	
Heterologous	175 (6.1%)	108 (8.9%)	67 (4.1%)	
Other	0 (0.0%)	0 (0.0%)	0 (0.0%)	
Unknown	0 (0.0%)	0 (0.0%)	0 (0.0%)	
(Missing/ Not applicable)	1,164	458	706	
**Number of vaccine doses**				**<0.001**
0	1,164 (29.0%)	458 (27.4%)	706 (30.1%)	
1	209 (5.2%)	91 (5.4%)	118 (5.0%)	
2	1,858 (46.3%)	682 (40.8%)	1,176 (50.1%)	
3	786 (19.6%)	439 (26.3%)	347 (14.8%)	
**Number of doses (unadjusted)**				**<0.001**
0	1,160 (28.9%)	456 (27.3%)	704 (30.0%)	
1	204 (5.1%)	87 (5.2%)	117 (5.0%)	
2	1,817 (45.2%)	653 (39.1%)	1,164 (49.6%)	
3	836 (20.8%)	474 (28.4%)	362 (15.4%)	
**Days since vaccination and illness onset**				**<0.001**
Median (IQR)	171.1 (81.0, 404.1)	102.1 (65.0, 176.3)	328.0 (118.0, 489.1)	
(Missing/ Not applicable)	1,164	458	706	

Variable	Overall, N = 4,017[1]	Group		p-value[2]
		**Cases**, N = 1,670[1]	**Control**, N = 2,347[1]	
**Days since vaccination and illness onset (group)**				**<0.001**
<90	828 (29.0%)	508 (41.9%)	320 (19.5%)	
90-<180	647 (22.7%)	407 (33.6%)	240 (14.6%)	
>=180	1,378 (48.3%)	297 (24.5%)	1,081 (65.9%)	
(Missing/ Not applicable)	1,164	458	706	
**Days between illness onset and test swab**				**<0.001**
Median (IQR)	4.0 (3.0, 6.0)	5.0 (3.0, 7.0)	3.0 (2.0, 5.0)	
**Days between illness onset and test swab (group)**				**<0.001**
<2 days	899 (22.4%)	260 (15.6%)	639 (27.2%)	
>14 days	4 (0.1%)	0 (0.0%)	4 (0.2%)	
2-7 days	2,623 (65.3%)	1,121 (67.1%)	1,502 (64.0%)	
7-14 days	491 (12.2%)	289 (17.3%)	202 (8.6%)	
**Days between illness onset and admission**				**<0.001**
Median (IQR)	3.0 (2.0, 5.0)	4.0 (3.0, 6.0)	2.0 (2.0, 4.0)	
**Days between illness onset and admission (group)**				**<0.001**
<2 days	1,624 (40.4%)	359 (21.5%)	1,265 (53.9%)	
2-7 days	2,130 (53.0%)	1,141 (68.3%)	989 (42.1%)	
7-14 days	263 (6.5%)	170 (10.2%)	93 (4.0%)	
**Any underlying health condition**	2,471 (61.5%)	867 (51.9%)	1,604 (68.3%)	**<0.001**
**Number of comorbidities**				**<0.001**
0	1,571 (39.1%)	806 (48.3%)	765 (32.6%)	
1	984 (24.5%)	407 (24.4%)	577 (24.6%)	
2	1,462 (36.4%)	457 (27.4%)	1,005 (42.8%)	
**Required supplementary oxygen**	2,291 (57.0%)	976 (58.4%)	1,315 (56.0%)	0.13
**Mechanical ventilation**	186 (4.6%)	90 (5.4%)	96 (4.1%)	0.054
**ICU admission**	784 (19.5%)	335 (20.1%)	449 (19.1%)	0.46
**In-hospital death**				0.15
	3 (0.1%)	0 (0.0%)	3 (0.1%)	
No	3,713 (92.4%)	1,533 (91.8%)	2,180 (92.9%)	
Yes	301 (7.5%)	137 (8.2%)	164 (7.0%)	
**Outcome of hospital stay**				0.15
Discharged from hospital	3,713 (92.4%)	1,533 (91.8%)	2,180 (92.9%)	
Transferred	3 (0.1%)	0 (0.0%)	3 (0.1%)	
Died in hospital	301 (7.5%)	137 (8.2%)	164 (7.0%)	

Variable	Overall, N = 4,017[1]	Group		p-value[2]
		**Cases**, N = 1,670[1]	**Control**, N = 2,347[1]	
**Study site**				**<0.001**
Chaharbakht	415 (10.3%)	280 (16.8%)	135 (5.8%)	
Fars	184 (4.6%)	105 (6.3%)	79 (3.4%)	
Gilan	365 (9.1%)	218 (13.1%)	147 (6.3%)	
Hamedan	275 (6.8%)	153 (9.2%)	122 (5.2%)	
Karak Hospital	364 (9.1%)	37 (2.2%)	327 (13.9%)	
Kermanshah	122 (3.0%)	77 (4.6%)	45 (1.9%)	
King Abdullah Hospital	827 (20.6%)	114 (6.8%)	713 (30.4%)	
Kurdistan	149 (3.7%)	80 (4.8%)	69 (2.9%)	
Mazandaran	549 (13.7%)	376 (22.5%)	173 (7.4%)	
Prince Hamza Hospital	305 (7.6%)	61 (3.7%)	244 (10.4%)	
Shahroud	215 (5.4%)	159 (9.5%)	56 (2.4%)	
Zarka Hospital	247 (6.1%)	10 (0.6%)	237 (10.1%)	
**Study country**				**<0.001**
Iran	2,274 (56.6%)	1,448 (86.7%)	826 (35.2%)	
Jordan	1,743 (43.4%)	222 (13.3%)	1,521 (64.8%)	

5.2 VE results separate for ICU admission and death

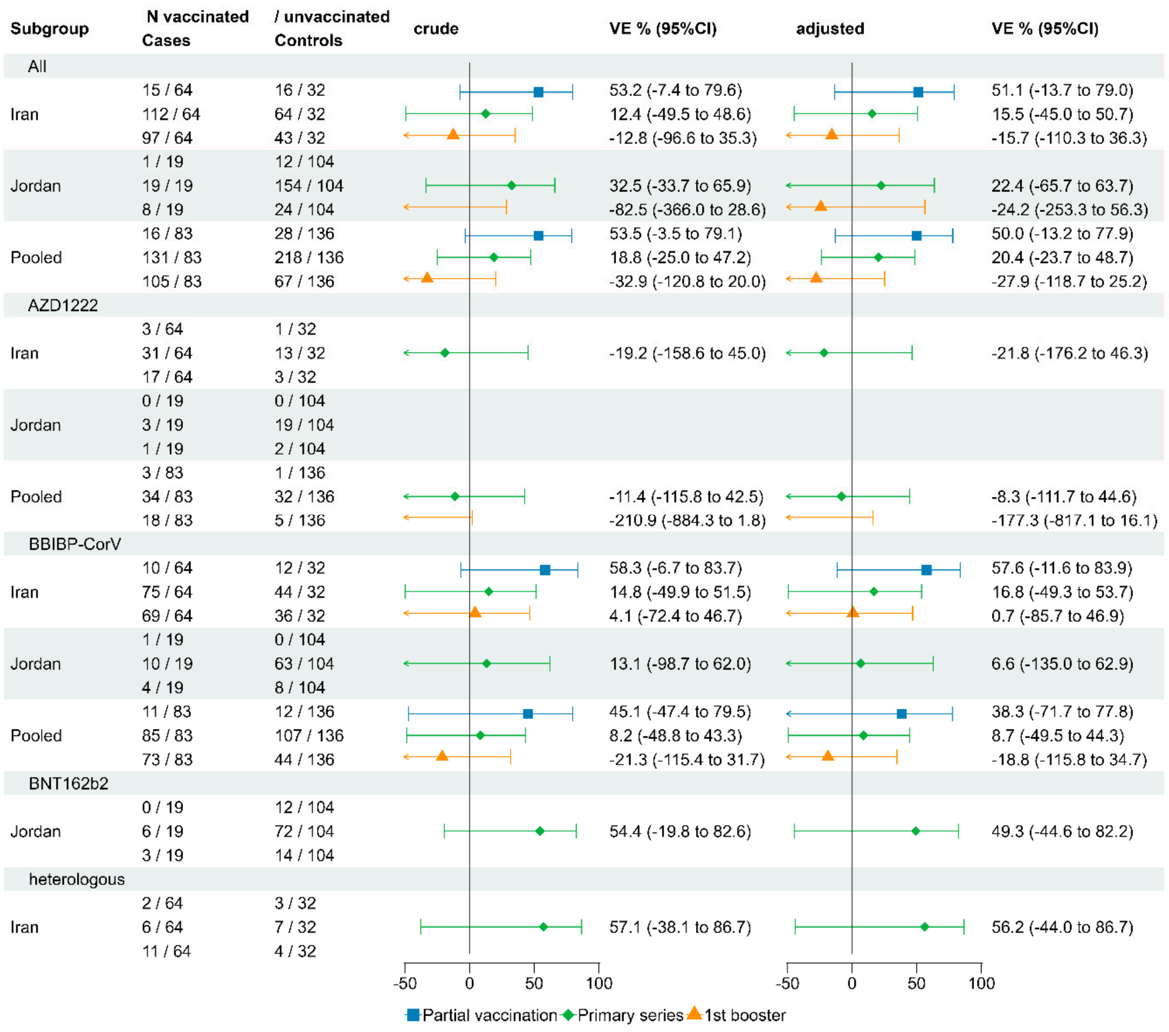

Subgroup	N vaccinated Cases	/ unvaccinated Controls	crude VE % (95%CI)	adjusted VE % (95%CI)
**All**				
Iran	15 / 64	16 / 32	53.2 (-7.4 to 79.6)	51.1 (-13.7 to 79.0)
	112 / 64	64 / 32	12.4 (-49.5 to 48.6)	15.5 (-45.0 to 50.7)
	97 / 64	43 / 32	-12.8 (-96.6 to 35.3)	-15.7 (-110.3 to 36.3)
Jordan	1 / 19	12 / 104		
	19 / 19	154 / 104	32.5 (-33.7 to 65.9)	22.4 (-65.7 to 63.7)
	8 / 19	24 / 104	-82.5 (-366.0 to 28.6)	-24.2 (-253.3 to 56.3)
Pooled	16 / 83	28 / 136	53.5 (-3.5 to 79.1)	50.0 (-13.2 to 77.9)
	131 / 83	218 / 136	18.8 (-25.0 to 47.2)	20.4 (-23.7 to 48.7)
	105 / 83	67 / 136	-32.9 (-120.8 to 20.0)	-27.9 (-118.7 to 25.2)
**AZD1222**				
Iran	3 / 64	1 / 32		
	31 / 64	13 / 32	-19.2 (-158.6 to 45.0)	-21.8 (-176.2 to 46.3)
	17 / 64	3 / 32		
Jordan	0 / 19	0 / 104		
	3 / 19	19 / 104		
	1 / 19	2 / 104		
Pooled	3 / 83	1 / 136		
	34 / 83	32 / 136	-11.4 (-115.8 to 42.5)	-8.3 (-111.7 to 44.6)
	18 / 83	5 / 136	-210.9 (-884.3 to 1.8)	-177.3 (-817.1 to 16.1)
**BBIBP-CorV**				
Iran	10 / 64	12 / 32	58.3 (-6.7 to 83.7)	57.6 (-11.6 to 83.9)
	75 / 64	44 / 32	14.8 (-49.9 to 51.5)	16.8 (-49.3 to 53.7)
	69 / 64	36 / 32	4.1 (-72.4 to 46.7)	0.7 (-85.7 to 46.9)
Jordan	1 / 19	0 / 104		
	10 / 19	63 / 104	13.1 (-98.7 to 62.0)	6.6 (-135.0 to 62.9)
	4 / 19	8 / 104		
Pooled	11 / 83	12 / 136	45.1 (-47.4 to 79.5)	38.3 (-71.7 to 77.8)
	85 / 83	107 / 136	8.2 (-48.8 to 43.3)	8.7 (-49.5 to 44.3)
	73 / 83	44 / 136	-21.3 (-115.4 to 31.7)	-18.8 (-115.8 to 34.7)
**BNT162b2**				
Jordan	0 / 19	12 / 104		
	6 / 19	72 / 104	54.4 (-19.8 to 82.6)	49.3 (-44.6 to 82.2)
	3 / 19	14 / 104		
**heterologous**				
Iran	2 / 64	3 / 32		
	6 / 64	7 / 32	57.1 (-38.1 to 86.7)	56.2 (-44.0 to 86.7)
	11 / 64	4 / 32		

***Figure S2.8**: Absolute VE against ICU admission among SARI patients in the TND studies. Blank VEs indicate insufficient data to be computed.*

Subgroup	N vaccinated Cases	/ unvaccinated Controls	crude VE % (95%CI)	adjusted VE % (95%CI)
**All**				
Iran	10 / 30	5 / 13	-122.2 (-1217.2 to 62.5)	-143.0 (-2069.7 to 72.8)
	34 / 30	10 / 13	-52.7 (-324.3 to 45.0)	-33.8 (-316.6 to 57.0)
	32 / 30	14 / 13	-33.1 (-275.9 to 52.8)	-15.7 (-276.8 to 64.5)
Jordan	1 / 15	4 / 51		
	12 / 15	53 / 51	28.1 (-72.6 to 70.0)	22.3 (-110.4 to 71.3)
	3 / 15	14 / 51		
Pooled	11 / 45	9 / 64	-61.4 (-465.8 to 53.9)	-47.0 (-440.4 to 60.0)
	46 / 45	63 / 64	3.7 (-86.1 to 50.1)	2.6 (-96.2 to 51.6)
	35 / 45	28 / 64	0.0 (-126.0 to 55.8)	25.8 (-77.9 to 69.1)
**AZD1222**				
Iran	1 / 30	0 / 13		
	9 / 30	2 / 13		
	8 / 30	1 / 13		
Jordan	0 / 15	0 / 51		
	1 / 15	14 / 51		
	0 / 15	1 / 51		
Pooled	1 / 45	0 / 64		
	10 / 45	16 / 64	45.2 (-75.8 to 82.9)	55.6 (-54.0 to 87.2)
	8 / 45	2 / 64		
**BBIBP-CorV**				
Iran	9 / 30	3 / 13		
	22 / 30	6 / 13	-67.3 (-440.6 to 48.2)	-36.0 (-386.7 to 62.0)
	21 / 30	12 / 13	2.0 (-191.5 to 67.1)	17.8 (-180.7 to 75.9)
Jordan	1 / 15	0 / 51		
	6 / 15	21 / 51	2.1 (-197.1 to 67.8)	-4.2 (-260.1 to 69.8)
	3 / 15	5 / 51		
Pooled	10 / 45	3 / 64		
	28 / 45	27 / 64	-32.7 (-192.8 to 39.8)	-19.4 (-173.7 to 47.9)
	24 / 45	17 / 64	-18.9 (-202.3 to 53.3)	13.6 (-129.3 to 67.4)
**BNT162b2**				
Jordan	0 / 15	4 / 51		
	5 / 15	18 / 51	13.0 (-182.9 to 73.2)	-3.8 (-288.4 to 72.2)
	0 / 15	8 / 51		
**heterologous**				
Iran	0 / 30	2 / 13		
	3 / 30	2 / 13		
	3 / 30	1 / 13		

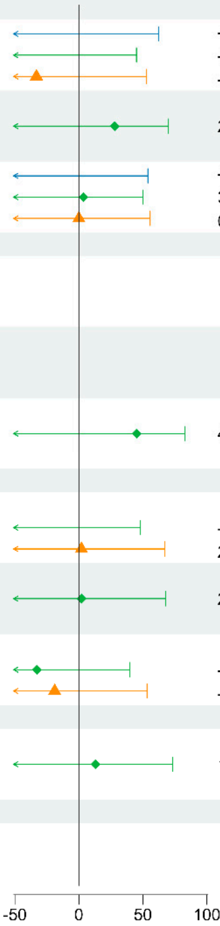

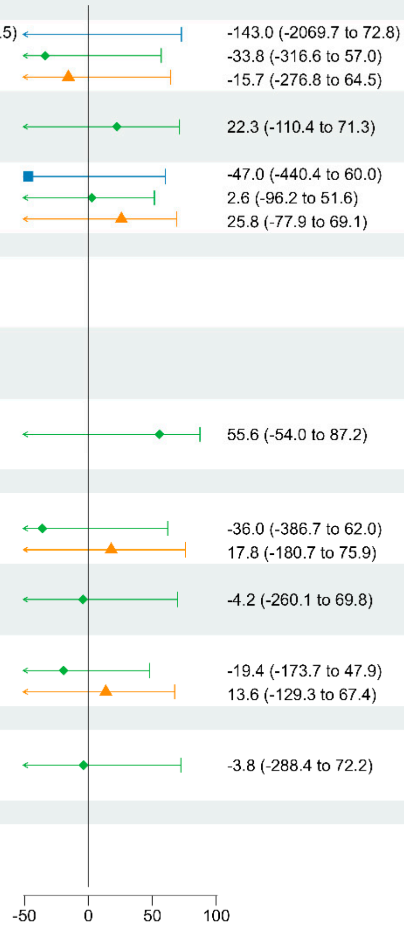

Partial vaccination ◆ Primary series ▲ 1st booster

***Figure S2.9**: Absolute VE against death among SARI patients in the TND studies. Blank VEs indicate insufficient data to be computed.*

5.3 Subgroup analysis results in TND studies

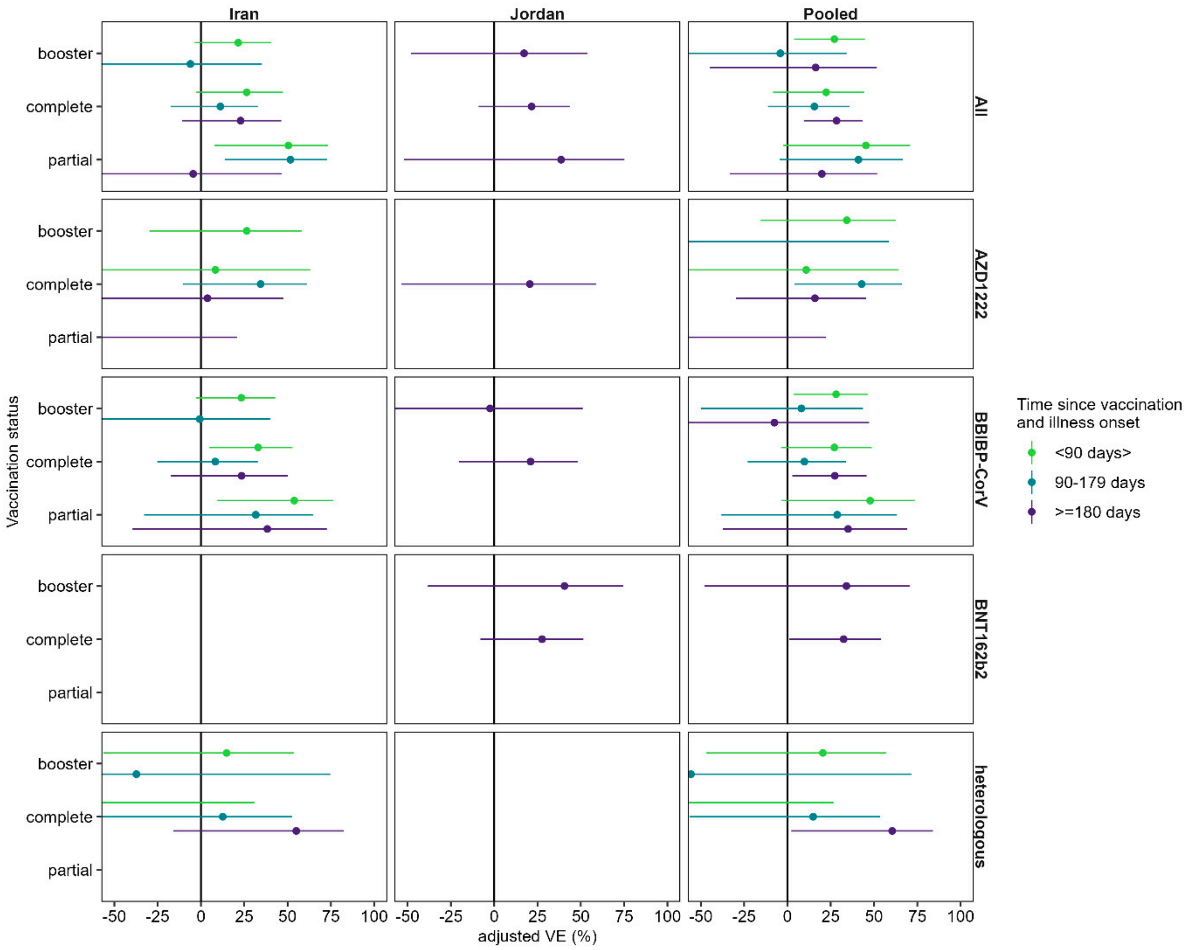

***Figure S2.10**: Adjusted VE against hospitalization by time since vaccination using pooled data from the two TND studies in Iran and Jordan.*
Heterologous is including "other" mixed vaccines.

***Table S2.16:** Adjusted VE against hospitalization by time since vaccination using pooled data from the TND studies*

Vaccine type	Vaccination status	country	<90 days	90-179 days	>=180 days
**All**	partial	Iran	50.5 (7.8 to 73.4)	51.7 (13.7 to 72.9)	-4.5 (-104.9 to 46.7)
		Jordan	NA	NA	38.6 (-52.0 to 75.2)
		Pooled	45.3 (-2.5 to 70.8)	41.0 (-4.5 to 66.7)	19.9 (-33.2 to 51.8)
	**primary series**	Iran	26.4 (-2.9 to 47.4)	11.2 (-17.4 to 32.8)	22.9 (-10.8 to 46.4)
		Jordan	NA	NA	21.6 (-9.0 to 43.7)
		**Pooled**	22.4 (-8.3 to 44.4)	15.6 (-11.2 to 35.9)	**28.4 (9.5 to 43.3)**
	booster	Iran	21.5 (-3.7 to 40.6)	-6.1 (-73.7 to 35.2)	NA
		Jordan	NA	NA	17.3 (-48.0 to 53.7)
		Pooled	27.1 (3.8 to 44.8)	-4.1 (-64.7 to 34.2)	16.3 (-44.9 to 51.7)
AZD1222	partial	Iran	NA	NA	-268.3 (-1612.0 to 20.8)
		Pooled	NA	NA	-166.3 (-811.1 to 22.1)
		Iran	8.4 (-127.8 to 63.1)	34.4 (-10.4 to 61.1)	3.8 (-76.7 to 47.6)

**Vaccine type**	**Vaccination status**	**country**	**<90 days**	**90-179 days**	**>=180 days**
	primary series	Jordan	NA	NA	20.6 (-53.7 to 59.0)
		**Pooled**	10.9 (-122.5 to 64.3)	**42.9 (4.0 to 66.0)**	15.9 (-29.7 to 45.5)
	booster	Iran	26.4 (-29.8 to 58.3)	NA	NA
		Pooled	34.3 (-15.5 to 62.6)	-398.5 (-5891.3 to 58.5)	NA
**BBIBP-CorV**	partial	Iran	53.9 (9.2 to 76.5)	31.7 (-32.8 to 64.8)	38.3 (-39.5 to 72.7)
		Pooled	47.7 (-3.5 to 73.6)	28.7 (-38.1 to 63.2)	35.0 (-37.4 to 69.3)
	**primary series**	**Iran**	**33.0 (4.7 to 52.9)**	8.3 (-25.3 to 32.8)	23.5 (-17.3 to 50.1)
		Jordan	NA	NA	21.0 (-20.3 to 48.2)
		**Pooled**	27.1 (-3.7 to 48.7)	9.8 (-22.9 to 33.8)	**27.4 (2.8 to 45.7)**
	**booster**	Iran	23.3 (-2.9 to 42.9)	-0.7 (-68.6 to 39.9)	NA
		Jordan	NA	NA	-2.4 (-114.6 to 51.2)
		**Pooled**	**28.1 (3.5 to 46.4)**	8.1 (-50.0 to 43.7)	-7.5 (-118.9 to 47.2)
BNT162b2	primary series	Jordan	NA	NA	27.6 (-8.0 to 51.5)
	booster	Jordan	NA	NA	40.6 (-38.4 to 74.5)
**Heterologous/ Other**	primary series	Iran	-218.9 (-1376.3 to 31.1)	12.6 (-61.3 to 52.6)	55.0 (-16.0 to 82.6)
	**primary series**	**Pooled**	-242.6 (-1499.5 to 26.6)	14.9 (-56.5 to 53.7)	**60.5 (2.2 to 84.0)**
	booster	Iran	14.8 (-56.2 to 53.5)	-37.3 (-644.3 to 74.7)	NA
	booster	Pooled	20.5 (-46.8 to 56.9)	-55.7 (-750.7 to 71.5)	NA

NA could not be computed due to insufficient data.

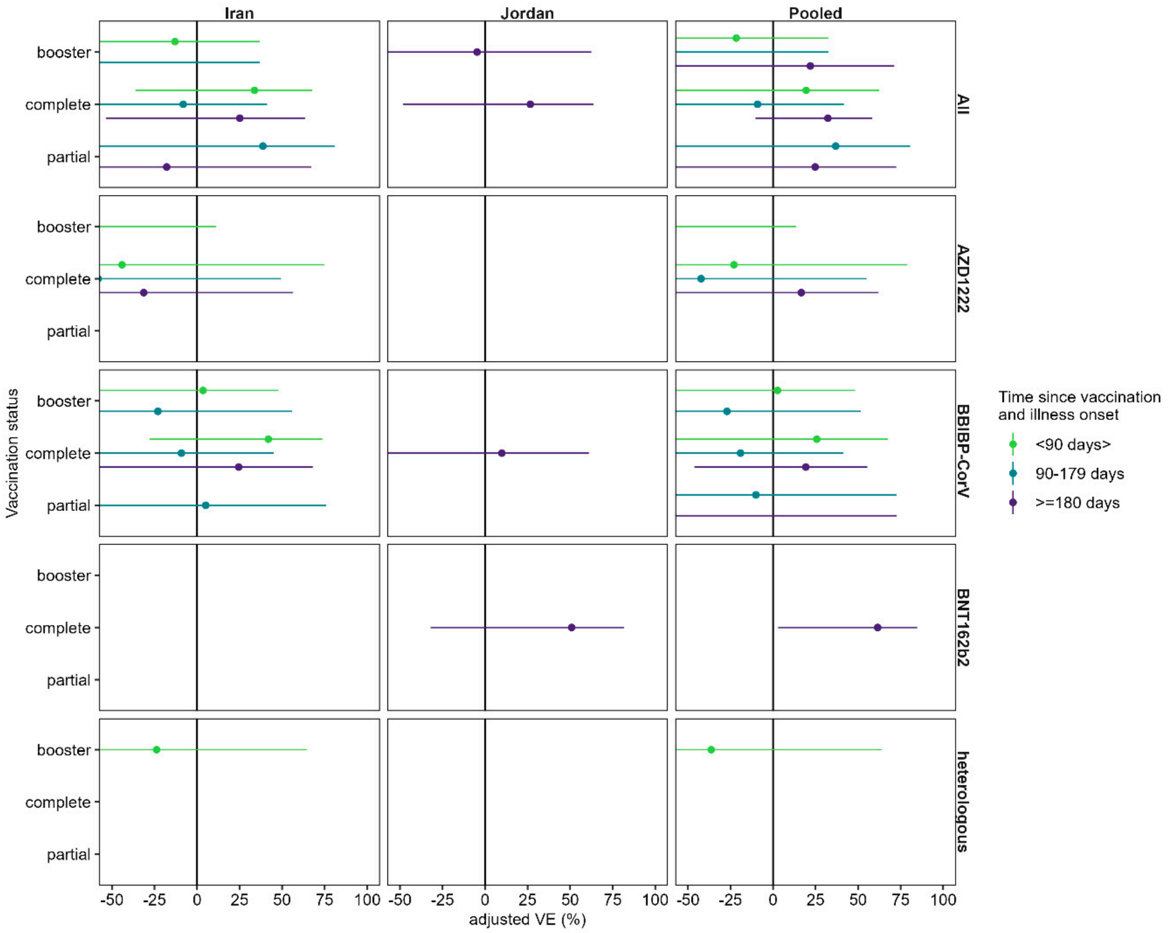

***Figure S2.11**: Adjusted VE against ICU admission or death (in-hospital-mortality), by time since vaccination using pooled data from the two TND studies in Iran and Jordan.*
Heterologous is including "other" mixed vaccines.

***Table S2.17**: VE against ICU admission/death by time since vaccination using pooled data from the TND studies*

Vaccine type	Vaccination status	country	<90 days>	90-179 days	>=180 days
All	partial	Iran	NA	38.7 (-96.6 to 80.9)	-17.8 (-323.8 to 67.2)
		Pooled	NA	36.8 (-105.9 to 80.6)	24.7 (-105.4 to 72.4)
	primary series	Iran	33.8 (-36.3 to 67.8)	-8.1 (-98.8 to 41.2)	25.1 (-53.5 to 63.4)
		Jordan	NA	NA	26.6 (-48.3 to 63.7)
		Pooled	19.4 (-72.3 to 62.3)	-9.1 (-103.9 to 41.6)	32.2 (-10.3 to 58.4)
	booster	Iran	-13.0 (-102.4 to 37.0)	-63.8 (-325.4 to 36.9)	NA
		Jordan	NA	NA	-4.7 (-192.1 to 62.5)
		Pooled	-21.6 (-119.6 to 32.7)	-64.3 (-300.5 to 32.6)	21.9 (-112.2 to 71.3)
AZD1222	primary series	Iran	-44.1 (-727.8 to 74.9)	-58.1 (-393.7 to 49.4)	-31.3 (-295.5 to 56.4)
		Pooled	-23.0 (-621.7 to 79.0)	-42.3 (-348.9 to 54.9)	16.6 (-83.0 to 62.0)
	booster	Iran	-250.5 (-1284.1 to 11.2)	NA	NA
		Pooled	-234.8 (-1199.0 to 13.7)	NA	NA
BBIBP-CorV	partial	Iran	NA	5.1 (-271.9 to 75.8)	NA
		Pooled	NA	-10.1 (-340.3 to 72.5)	-85.5 (-1159.4 to 72.7)
	primary series	Iran	41.9 (-28.0 to 73.6)	-9.2 (-117.3 to 45.1)	24.5 (-78.1 to 68.0)
		Jordan	NA	NA	9.9 (-108.2 to 61.0)

Vaccine type	Vaccination status	country	<90 days>	90-179 days	>=180 days
		Pooled	25.7 (-70.3 to 67.6)	-19.1 (-142.1 to 41.4)	19.2 (-46.2 to 55.4)
	booster	Iran	3.5 (-78.8 to 47.9)	-23.0 (-242.9 to 55.9)	NA
		Pooled	2.6 (-83.4 to 48.3)	-27.1 (-233.7 to 51.6)	NA
**BNT162b2**	**primary series**	Jordan	NA	NA	50.9 (-32.0 to 81.7)
		**Pooled**	NA	NA	**61.5 (3.0 to 84.7)**
Other	booster	Iran	-23.8 (-335.1 to 64.8)	NA	NA

NA could not be computed due to insufficient data.

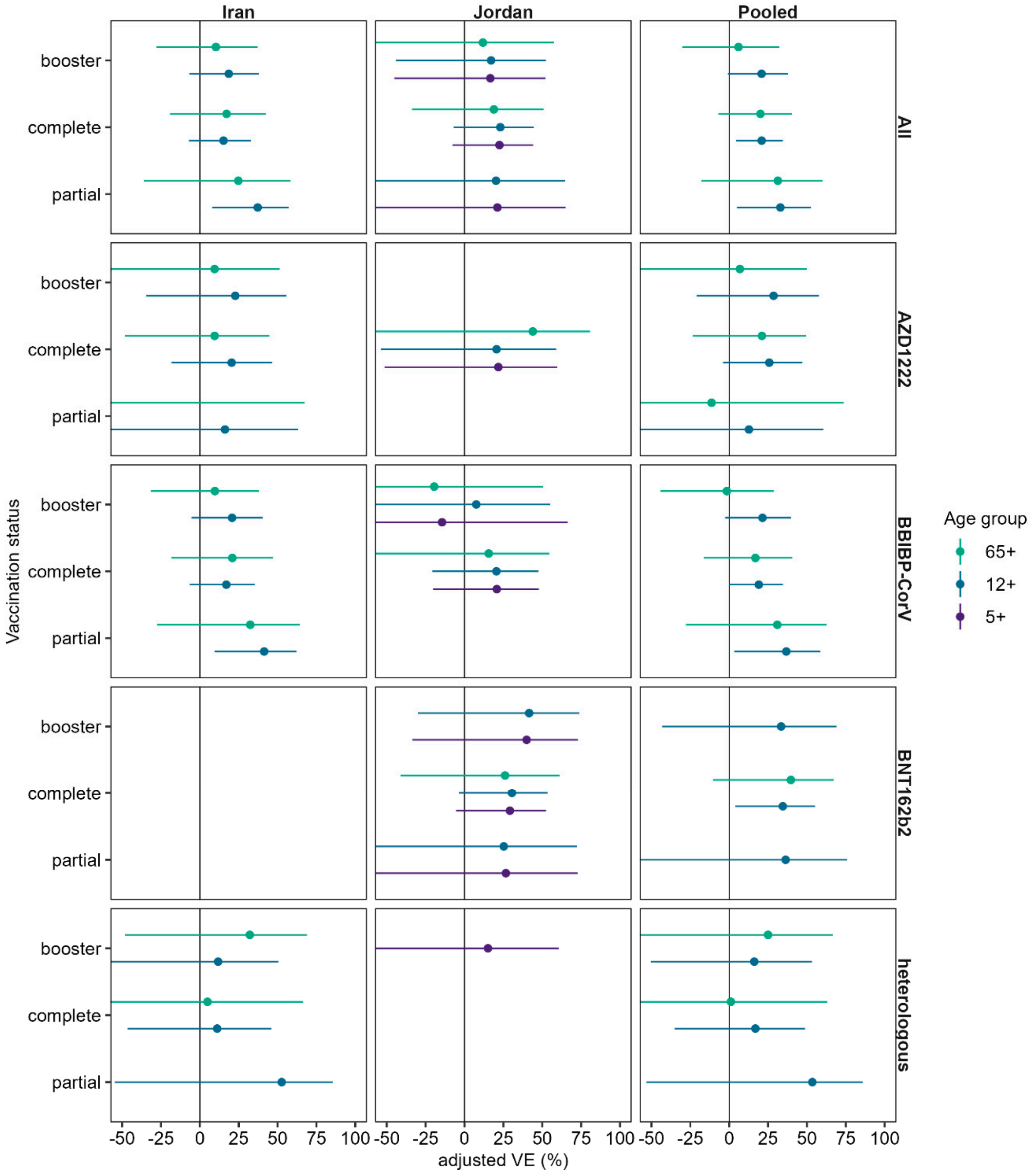

***Figure S2.12**: Adjusted VE against hospitalization by age group , all above 11 years, and age 65 years and older, using pooled data from the two TND studies in Iran and Jordan. Age group 5-11 only applicable to Jordan. Heterologous is including "other" mixed vaccines.*

Table S2.18: *Adjusted VE against hospitalization by age group , all above 11 years, and age 65 years and older, using pooled data from the two TND studies in Iran and Jordan. Age group 5-11 only applicable to Jordan.*

Vaccine type	Vaccination status	country	Age 12+	Age 65+	Age 5+ (Jordan only)
All	partial	Iran	37.2 (7.9 to 57.2)	24.8 (-36.0 to 58.4)	NA
		Jordan	20.3 (-79.6 to 64.6)	NA	21.2 (-77.5 to 65.0)
		Pooled	32.9 (5.0 to 52.7)	31.3 (-17.9 to 60.0)	NA
	primary series	Iran	15.3 (-7.0 to 32.9)	17.2 (-19.1 to 42.5)	NA
		Jordan	23.0 (-6.9 to 44.6)	18.9 (-33.7 to 50.8)	22.5 (-7.7 to 44.2)
		Pooled	20.9 (4.5 to 34.5)	20.1 (-7.0 to 40.4)	NA
	booster	Iran	18.6 (-6.7 to 37.9)	10.4 (-27.9 to 37.2)	NA
		Jordan	17.1 (-44.2 to 52.3)	11.8 (-83.0 to 57.5)	16.6 (-45.0 to 52.1)
		Pooled	20.8 (-0.9 to 37.9)	6.0 (-30.2 to 32.2)	NA
AZD1222	partial	Iran	16.2 (-90.9 to 63.2)	-64.1 (-726.9 to 67.4)	NA
		Pooled	12.7 (-93.7 to 60.7)	-11.4 (-368.8 to 73.5)	NA
	primary series	Iran	20.5 (-18.2 to 46.5)	9.5 (-48.3 to 44.8)	NA
		Jordan	20.6 (-53.7 to 59.0)	43.9 (-63.0 to 80.7)	21.8 (-51.5 to 59.6)
		Pooled	25.8 (-4.0 to 47.1)	21.0 (-23.4 to 49.5)	NA
	booster	Iran	22.8 (-34.5 to 55.7)	9.5 (-68.3 to 51.3)	NA
		Pooled	28.5 (-20.9 to 57.7)	6.9 (-73.7 to 50.1)	NA
BBIBP-CorV	partial	Iran	41.4 (9.4 to 62.1)	32.5 (-27.5 to 64.2)	NA
		Pooled	36.7 (3.2 to 58.6)	30.9 (-27.8 to 62.7)	NA
	primary series	Iran	17.0 (-6.5 to 35.4)	20.9 (-18.3 to 47.1)	NA
		Jordan	20.5 (-20.7 to 47.6)	15.6 (-56.7 to 54.5)	20.8 (-20.2 to 47.8)
		Pooled	19.1 (-0.1 to 34.6)	16.9 (-16.3 to 40.6)	NA
	booster	Iran	20.8 (-5.4 to 40.4)	9.7 (-31.5 to 38.0)	NA
		Jordan	7.6 (-89.8 to 55.0)	-19.5 (-188.9 to 50.6)	-14.4 (-289.9 to 66.4)
		Pooled	21.4 (-2.5 to 39.8)	-1.5 (-44.3 to 28.6)	NA
BNT162b2	partial	Jordan	25.3 (-101.5 to 72.3)	NA	26.6 (-97.7 to 72.8)
	primary series	Jordan	30.5 (-3.6 to 53.4)	26.1 (-41.1 to 61.3)	29.2 (-5.4 to 52.5)
	booster	Jordan	41.6 (-30.0 to 73.7)	NA	40.0 (-33.5 to 73.0)
Other	partial	Iran	52.7 (-54.8 to 85.5)	NA	NA
	primary series	Iran	11.2 (-46.4 to 46.1)	4.9 (-169.7 to 66.5)	NA
	booster	Iran	11.8 (-57.3 to 50.5)	32.2 (-48.1 to 68.9)	NA

NA could not be computed due to insufficient data.

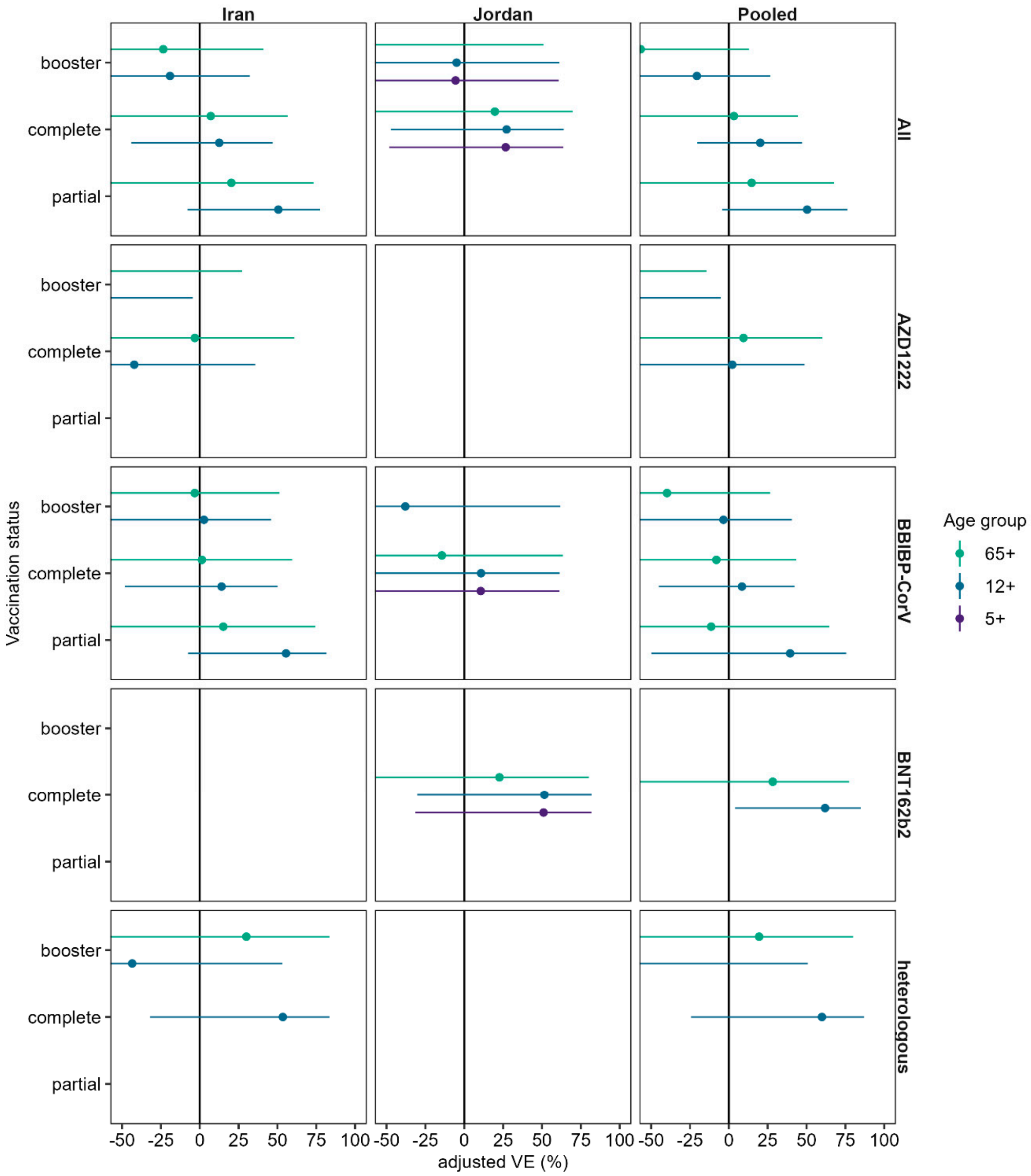

Figure S2.13: *Adjusted VE against ICU admission and or death (in-hospital mortality), by age group , all above 11 years, and age 65 years and older, using pooled data from the two TND studies in Iran and Jordan. Age group 5-11 only applicable to Jordan.*
Heterologous is including "other" mixed vaccines.

Table S2.19: *Adjusted VE against ICU admission and or death (in-hospital mortality), by age group , all above 11 years, and age 65 years and older, using pooled data from the two TND studies in Iran and Jordan. Age group 5-11 only applicable to Jordan.*

Vaccine type	Vaccination status	country	Age 12+	Age 65+	Age 5+ (Jordan only)
All	partial	Iran	50.6 (-7.8 to 77.4)	20.4 (-136.4 to 73.2)	NA

Vaccine type	Vaccination status	country	Age 12+	Age 65+	Age 5+ (Jordan only)
	partial	Pooled	50.4 (-4.3 to 76.4)	14.7 (-125.4 to 67.7)	NA
	primary series	Iran	12.6 (-44.0 to 46.9)	7.1 (-98.8 to 56.6)	NA
	primary series	Jordan	27.2 (-47.1 to 64.0)	19.7 (-113.0 to 69.7)	26.6 (-48.3 to 63.7)
	primary series	Pooled	20.2 (-20.3 to 47.1)	3.2 (-68.5 to 44.5)	NA
	booster	Iran	-19.1 (-109.1 to 32.2)	-23.5 (-158.9 to 41.1)	NA
	booster	Jordan	-4.9 (-183.5 to 61.2)	-62.0 (-434.5 to 50.9)	-5.6 (-184.6 to 60.8)
	booster	Pooled	-20.6 (-98.1 to 26.6)	-56.7 (-181.9 to 12.9)	NA
AZD1222	primary series	Iran	-42.1 (-214.4 to 35.7)	-3.2 (-172.3 to 60.9)	NA
	primary series	Pooled	2.2 (-86.4 to 48.7)	9.4 (-106.2 to 60.2)	NA
	booster	Iran	-305.3 (-1473.5 to -4.4)	-192.4 (-1077.3 to 27.4)	NA
	booster	Pooled	-231.5 (-943.9 to -5.3)	-281.7 (-1173.7 to -14.4)	NA
BBIBP-CorV	partial	Iran	55.5 (-7.4 to 81.6)	15.2 (-179.6 to 74.3)	NA
	partial	Pooled	39.5 (-49.9 to 75.5)	-11.4 (-250.9 to 64.6)	NA
	primary series	Iran	14.1 (-48.2 to 50.2)	1.4 (-140.0 to 59.5)	NA
	primary series	Jordan	10.8 (-106.2 to 61.4)	-14.4 (-258.1 to 63.4)	10.6 (-106.7 to 61.3)
	primary series	Pooled	8.4 (-45.0 to 42.2)	-8.0 (-106.5 to 43.5)	NA
	booster	Iran	2.7 (-74.8 to 45.9)	-3.2 (-118.7 to 51.3)	NA
	booster	Jordan	-37.9 (-398.2 to 61.8)	NA	NA
	booster	Pooled	-3.5 (-80.1 to 40.6)	-39.8 (-165.7 to 26.5)	NA
BNT162b2	primary series	Jordan	51.6 (-30.2 to 82.0)	22.6 (-201.6 to 80.1)	51.0 (-31.5 to 81.8)
Other	primary series	Iran	53.5 (-32.0 to 83.6)	NA	NA
	booster	Iran	-43.4 (-339.9 to 53.2)	30.0 (-199.3 to 83.6)	NA
