# Supplementary material for: COVID-19 Vaccine Effectiveness Studies against Symptomatic and Severe Outcomes during the Omicron Period in Four Countries in the Eastern Mediterranean Region
Source: Vaccines (Basel). 2024 Aug 10;12(8):906. doi: 10.3390/vaccines12080906 (PMC11360574; doi:10.3390/vaccines12080906)
Supplement: Supplementary file 1 [file vaccines-12-00906-s001.zip › File S3 - Lessons learned table.pdf]

COVID-19 vaccine effectiveness against symptomatic and severe outcomes during the Omicron period in four countries in the Eastern Mediterranean Region

Table S3.1: Lessons learned during the analysis of COVID-19 vaccine effectiveness studies in Eastern Mediterranean Region

	Category	Challenge	Example	Solutions adopted by the WHO / lessons learned
	STUDY CONCEPTUALIZATION AND PLANNING			
1	**Timing of study**	VE effectiveness in a pandemic situation is likely to change over time as a result of the depletion of susceptible individuals in a population, the evolution of the pathogen and waning immunity.	Studies revealed lower-than-expected VE rates and even negative VE outcomes.<br>The time that elapsed between vaccination and onset of illness or start of follow up in cohort studies was for the majority of study participants longer than 6 months.<br><br>In Jordan even longer than a year.<br><br>In Pakistan only four unvaccinated health workers (0.3% of the total cohort included in the analysis) were enrolled.	1.1. VE studies should be planned early during a pandemic with participant enrolment as soon as possible after vaccination becomes available.<br>1.2. Pandemic preparedness should also include preparedness to plan and conduct VE studies<br>1.3. In the selection of the study population, the epidemiological context, seroprevalence and vaccination rates should be carefully reviewed.
2	**Adherence to protocols**	Adaptation of VE guidelines from existing protocols, such as those for seasonal flu, proved inadequate for the unique challenges posed by COVID-19.	The four studies utilized the same WHO VE protocols, and adapted those to their specific COVID-19 contexts in countries.<br><br>Some deviations existed in the assumptions made for the sample size calculation, even for studies using the same design.	2.1. Challenge to obtain full adherence to guidelines and protocols when conducting complicated observational vaccine studies during emergencies should be acknowledged and adaptations at the country level minimized as much as possible.

			Other logistical challenges during implementation included global shortage of tests and kits. Public holidays were also mentioned as a reason for missed follow ups and tests in the cohort studies.<br><br>Implementation challenges are also addressed in a previous publication by Kheirandish et al 2023[1]	2.2. Contingency plans for study logistics should be made more robust for VE studies conducted during a pandemic, this may include more exhaustive risk assessment, timeline buffer and backups or alternative service providers.
**3**	**Study platform or surveillance systems**	When studies are conducted for the first time in a country, surveillance systems or national data collection platforms might not yet exist.<br><br>In such a case, reaching sufficiently large sample sizes can take substantial amount of effort.	Most of the four studies were implemented for the first time with limited availability of large nationwide platforms that can be leveraged for COVID-19 VE studies.<br><br>Jordan's study did build on an existing influenza SARI surveillance system in four sentinel sites. And in Iran's study a national registration system registering COVID-19 vaccines and all SARI cases hospitalized was used.	3.1. Leveraging existing platforms would facilitate better preparedness for future pandemics, minimizing technical implementation issues and protocol deviations.

[1] 1. Kheirandish M, Karimian Z, Fahmy K, Rashidian A, Hajjeh R. Capacity-building for conducting COVID-19 vaccine effectiveness studies to enhance evidence-informed vaccination policymaking in the Eastern Mediterranean Region. East Mediterr Health J. 2023;29: 562–569. doi:10.26719/emhj.23.094

**4**	**Sample size**	Insufficient sample size limits the statistical power to obtain precise VE estimates. It also limits the capacity to perform subgroup analyses as well as the applicability of the results across the entire vaccinated population.	In all four studies there were some inconclusive VE estimates with very wide confidence intervals.<br><br>While implementation and epidemiological challenges contributed (see timing of studies), sample size calculation might also have contributed to it. The sample size calculation was based on a single strata/subgroup and did not factor in the wide spectrum of vaccines used in the population, or other characteristics such as previous infection status, occupational risk of exposure, and variants.	4.1. More factors should be considered in sample size calculation such as vaccine type/brands and seroprevalence rates (previous infection status) (see 1).<br><br>4.2. Pre-existing or implementing new VE platform to allow reaching higher sample sizes more easily (see 3).
**5**	**Follow up in cohort studies**	Avoiding missing data in observational studies completely is nearly impossible, however the amount and location of missing information can greatly impact statistical analysis and validity of the results.	Partial and complete lost to follow ups occurred in the two cohort studies, and in all of the studies some information about potential confounding factors were either partly or completely missing. Notably, for some studies, national holidays and Ramadan impacted the testing of symptomatic participants – potentially introducing a bias.	5.1. Instructions to prevent loss of essential information in case of partial lost to follow-up should be more detailed and specific.<br><br>5.2. Communication with study participants at the enrolment, should also address how best to collect information from that individual in case of unavailability during follow-up.

				5.3. Incentives for optimizing follow-up and collection of specimens for laboratory confirmation might need to be improved to reduce study drop-outs.
	DATA MANAGEMENT, STUDY ANALYSIS AND INTERPRETATION			
**6**	**Data management, storage and cleaning**	Data management, proper storage and systematic cleaning procedures are crucial for any epidemiological and clinical study conducted. The challenges increases when multi-centre studies are conducted that require alignment for regional or pooled analysis.	The use of REDCap (an electronic central data entry platform) facilitated the use of standardised procedures on data collection and data cleaning to reduce bias and divergences in data handling between studies, a critical consideration for pooled analysis.<br><br>In one of the four countries, data cleaning was performed in parallel to cleaning on the REDCap platform.	6.1. Adherence to method guidelines and clear description of methods in statistical analysis plans should be promoted.<br><br>6.1. Pragmatical standardized procedures for data collection and details of statistical analysis should be provided.
**7**	**Reproducibility and version tracking of analysis**	Conducting multi-center studies during a pandemic poses challenges to reproducibility and version tracking due to rapid data collection, evolving protocols, decentralized operations, increasing the risk of inconsistencies, data errors, and protocol deviations that may compromise the integrity and comparability of analyses.	Across the four studies, data cleaning was tracked via log files on REDCap and statistical programming software were used by all study teams. However, the quality and completeness of the analysis scripts was not standardized and some data cleaning was also performed using coding software (outside of REDCap).	7.1. Reproducibility of analysis results should be made an objective early on, and the use of version-controlled data analysis strongly encouraged.

**8**	**Statistical analysis**	Statistical analysis guidelines have to be generic enough to ensure applicability across a wide range of contexts, with the expectation of further specification to be done by the local research teams.	Many questions were raised on the individual / national analyses performed by the four countries' study teams and challenges in comparison of VE estimates from two independent analysts performed.<br><br>For instance, how to calculate VE for secondary outcomes from the TND case controls studies using logistic regression. The criteria to use for selection of covariates also differed as did the estimation of crude estimates (with or without adjusting for study site).	8.1. Practical examples should be provided for the statistical analysis of CVE studies with recommended choices when multiple options exist.<br>8.2. Comparison of VE estimates generated by two independent groups should be encouraged as it is helpful to identify and resolve discrepancies.
**9**	**Pooling**	Gaps in the adherence to protocols can impact sample size, data quality, the comparison of results or the pooling across studies.	Two different study designs (cohort and TND) were used across the four studies, limiting the possibility of pooling data across all four studies. Even among studies of the same study design, heterogeneity resulted in pooling not providing additional benefit.	9.1. The same study design and standardized protocols across different study research teams/projects should be adopted to allow for pooled analysis<br>9.2. Study timing and implementation should be harmonized, and observance of protocols ensured.
	PRESENTATION AND INTERPRETATION OF RESULTS			
**10**	**Presentation and interpretation of results**	Correct interpretation of results is often complex due interaction between multiple factors, such as epidemiological context, study limitations, and residual confounding.	Estimated VE in the studies was lower than expected and sometimes even negative, posing a challenge for their interpretation and use for informing vaccination policies.	10.1. Templates including examples for correct presentation should be provided.<br>10.2. Assistance in correct interpretation of results should be made available during and after the analysis.
	STUDY COORDINATION AND TRAINING			
**11**	**Coordination**	Multi-center studies are challenging as they involve many different	The WHO-EMRO provided regular technical support and facilitated communication and	11.1. Technical support should be provided to ensure alignment

		institutions across different countries and epidemiological situations in countries. In addition, the level of expertise of research teams and availability of resources likely vary.	coordination of the study planning, data collection and analysis.	between studies and best possible adherence to protocols especially when results are feeding into the same platform to inform policy decisions.
**12**	**Capacity strengthening**	Global VE guidelines, while helpful, are theoretical and cannot provide the same value and learning experience as an exchange across PIs facing similar challenges and questions.	Some PIs were conducting a VE study on a national level for the first time, hence requiring additional technical support from experts and opportunities for exchange with their peers.	12.1. Experts should be involved from the very beginning of the study and ensure continuity of technical assistance across the whole study path.
				12.2. Meetings and workshops should be promoted as they facilitate coordination and alignment across studies by allowing study PIs to have an exchange with peers and experts.
