# Supplementary material for: COVID-19 Vaccine Effectiveness Studies against Symptomatic and Severe Outcomes during the Omicron Period in Four Countries in the Eastern Mediterranean Region
Source: Vaccines (Basel). 2024 Aug 10;12(8):906. doi: 10.3390/vaccines12080906 (PMC11360574; doi:10.3390/vaccines12080906)
Supplement: Supplementary file 1 [file vaccines-12-00906-s001.zip › File S4 - Consortium of Authors.pdf]

COVID-19 vaccine effectiveness against symptomatic and severe outcomes during the Omicron period in four countries in the Eastern Mediterranean Region

Supplementary File S4: 'Consortium of Authors' from participating countries in the Eastern Mediterranean Regional COVID-19 Vaccine Effectiveness Study

Egypt's National COVID-19 Vaccine Effectiveness Study (Cohort in HWs)

1. Areej Rushdi, Medical Microbiology and Immunology Department, Faculty of Medicine (Girls), Al-Azhar University, Cairo, Egypt
2. Aya Ghamry, Medical Microbiology and Immunology Department, Faculty of Medicine (Girls), Al-Azhar University, Cairo, Egypt
3. Arwa Kamhawy, Medical Microbiology and Immunology Department, Faculty of Medicine (Girls), Al-Azhar University, Cairo, Egypt
4. Asmaa M. El-Nasser, Medical Microbiology and Immunology Department, Faculty of Medicine (Girls), Al-Azhar University, Cairo, Egypt
5. Samy Zaky, Hepatology-Gastroenterology and Infectious Diseases Department, Al-Zahraa University Hospital, Al-Azhar University Cairo, Egypt
6. Sumyaa H. El Shazly, Hepatology-Gastroenterology and Infectious Diseases Department, Al-Zahraa University Hospital, Al-Azhar University Cairo, Egypt
7. Eman Elshemy, Hepatology-Gastroenterology and Infectious Diseases Department, Al-Zahraa University Hospital, Al-Azhar University, Cairo, Egypt
8. Alshaima Eid, Hepatology-Gastroenterology and Infectious Diseases Department, Al-Zahraa University Hospital, Al-Azhar University, Cairo, Egypt
9. Neamat Abdelmageed, Hepatology-Gastroenterology and Infectious Diseases Department, Al-Zahraa University Hospital, Al-Azhar University Cairo, Egypt
10. Shayma Mohammed, Clinical Pathology Department, Al-Zahraa University Hospital, Al-Azhar University Cairo, Egypt
11. Alshayma A. Abdel Alim, Clinical Pathology Department, Al-Zahraa University Hospital, Al-Azhar University, Cairo, Egypt
12. Amgad A. Elzahaby, Hepato-Gastroenterology and Infectious Diseases Department, Al-Hussein Hospital, Al-Azhar University Cairo, Egypt
13. Ahmed E. Ahmed , Hepato-Gastroenterology and Infectious Diseases Department, Al-Hussein Hospital, Al-Azhar University Cairo, Egypt
14. Ahmed S. Kadah, Dermatology and Andrology Department, Al-Hussein Hospital, Al-Azhar University Cairo, Egypt
15. Mohamed A. Shaheen, Clinical Pathology Department, Al-Hussein Hospital, Al-Azhar University Cairo, Egypt
16. Shaban Salah El Azhary, Hepatology-Gastroenterology, and Infectious Diseases Department, Bab-AlShareia Hospital, Al-Azhar University, Cairo, Egypt
17. Abdou M. Elshafei, Hepatology-Gastroenterology and Infectious Diseases Department, Bab-AlShareia Hospital, Al-Azhar University, Cairo, Egypt

18. Ibrahim M. Bauomy, Clinical Pathology Department, Bab-AlShareia Hospital Al-Azhar University, Cairo, Egypt
19. Mohamed M. Hegazy, Hepatology-Gastroenterology and Infectious Diseases Department, Bab-AlShareia Hospital Al-Azhar University, Cairo, Egypt
20. Walaa M. Omar Ashry, Medical Microbiology and Immunology Department**,** Damietta Faculty of Medicine (Girls), Al-Azhar University, Damietta, Egypt
21. Fathiya El-Raey Hepatology-Gastroenterology and Infectious Diseases Department, Damietta Faculty of Medicine, Al-Azhar University, Damietta, Egypt
22. Atef W. Elrifai, Chest Diseases Department**,** Damietta Faculty of Medicine, Al-Azhar University, Damietta, Egypt
23. Khaled A. Eid, Hepatology-Gastroenterology and Infectious Diseases Department, Al-Azhar University, Assuit, Egypt
24. Amro M. Hassan, Hepatology-Gastroenterology and Infectious Diseases Department, Al-Azhar University, Assuit, Egypt
25. Emad Abdelrazzak, Hepatology-Gastroenterology and Infectious Diseases Department, Al-Azhar University, Assuit, Egypt
26. Mustafa A. Haridy, Hepatology-Gastroenterology, and Infectious Diseases Department, Al-Azhar University, Assuit, Egypt
27. Somaia I. Salama, Community Medicine Research Department, National Research Centre, Cairo, Egypt
28. Ghada A. Elshaarawy, Community Medicine Research Department, National Research Centre, Cairo, Egypt
29. Doaa E. Ahmed, Community Medicine Research Department, National Research Centre, Cairo, Egypt
30. Hazem M. El Hariri Community Medicine Research Department, National Research Centre, Cairo, Egypt
31. Sherif E. Eldeeb, Community Medicine Research Department, National Research Centre, Cairo, Egypt

<u>Iran's National COVID-19 Vaccine Effectiveness Study (TND in SARI):</u>

1. Ghobad Moradi, Research Institute for Health Development, Kurdistan University of Medical Sciences, Sanandaj, Iran
2. Mohammadreza Naghipour, Gastrointestinal and Liver Diseases Research Center, Guilan University of Medical Sciences, Rasht, Iran
3. Fatemeh Torkaman Asadi, Infectious Disease Research Center, Hamadan University of Medical Sciences, Hamadan, Iran
4. Ali Ahmadi, Research Center in Health Modeling, Shahrekord University of Medical Sciences, Shahrekord, Iran
5. Alireza Mirahmadizadeh, Non-Communicable Diseases Research Center, Shiraz University of Medical Sciences, Shiraz, Iran

<u>Jordan's National COVID-19 Vaccine Effectiveness Study (TND in SARI):</u>

1. Reem Qudisat, Jordan Ministry of Health, Irbid, Jordan
2. Enas Bataineh, Jordan Ministry of Health, Irbid, Jordan
3. Shirin Jaradat, Jordan Ministry of Health, Irbid, Jordan

4. Saja Almustafa, Jordan Ministry of Health, Irbid, Jordan
5. Ali Banni Issa, Jordan Ministry of Health, Irbid, Jordan
6. Abeer Melhem, Jordan Ministry of Health, Amman, Jordan
7. Ahmad Murad, Jordan Ministry of Health, Amman, Jordan
8. Tareq Ibdah, Jordan Ministry of Health, Amman, Jordan
9. Saber Nassar, Jordan Ministry of Health, Amman, Jordan
10. Mona Abu Qaddum, Jordan Ministry of Health, Amman, Jordan
11. Marwan Alkhatib, Jordan Ministry of Health, Amman, Jordan
12. Lina Alamer, Jordan Ministry of Health, Zarqa, Jordan
13. Bissan Mahmoud, Jordan Ministry of Health, Zarqa, Jordan
14. Fatima Marakchi, Jordan Ministry of Health, Zarqa, Jordan
15. Lubna Hittini, Jordan Ministry of Health, Zarqa, Jordan
16. Harbieh Asi, Jordan Ministry of Health, Zarqa, Jordan
17. Fawaz Shawabkeh, Jordan Ministry of Health, Karak, Jordan
18. Itedal Aljaradat, Jordan Ministry of Health, Karak, Jordan
19. Rokaya Alnawaiseh, Jordan Ministry of Health, Karak, Jordan
20. Kouloud Allotaibat, Jordan Ministry of Health, Karak, Jordan
21. Suhaila Khalil, Jordan Ministry of Health, Amman, Jordan
22. Sawsan Abukharmeh, Jordan Ministry of Health, Amman, Jordan
23. Bahaaaldeen Alawaideh, Jordan Ministry of Health, Amman, Jordan
24. Iqbal Ghanem, Jordan Ministry of Health, Amman, Jordan
25. Huda Mustafa, Jordan Ministry of Health, Amman, Jordan
26. Sara Bobali, Jordan Ministry of Health, Amman, Jordan
27. Haneen AlHasni, Jordan Ministry of Health, Amman, Jordan
28. Nemat Tbakhi, Jordan Ministry of Health, Amman, Jordan
29. Layla Ghaffari, Jordan Ministry of Health, Amman, Jordan
